# Supplementary material for: Facile Synthesis of Tricyclic 1,2,4-Oxadiazolines-Fused Tetrahydro-Isoquinolines from Oxime Chlorides with 3,4-Dihydroisoquinoline Imines
Source: Molecules. 2022 May 10;27(10):3064. doi: 10.3390/molecules27103064 (PMC9145198; doi:10.3390/molecules27103064)
Supplement: Supplementary file 1 [file molecules-27-03064-s001.zip › molecules-1708110-supplementary.pdf]

Facile Synthesis of Tricyclic 1,2,4-Oxadiazolines-Fused Tetrahydro-Isoquinolines from Oxime Chlorides with 3,4-Dihydroisoquinoline Imines

Kaikai Wang [1,2], Yanli Li [3], Wei Zhang [1], Rongxiang Chen [1,*], Xueji Ma [1,2,*], Mingyue Wang [1] and Nan Zhou [1]

[1] School of Pharmacy, Xinxiang University, Xinxiang 453000, China; wangkaikai@xxu.edu.cn (K.W.); xinxiangzhangwei@126.com (W.Z.); wmyoutstanding@163.com (M.W.); zhounan5180708@163.com (N.Z.)
[2] Key Laboratory of Nano-Carbon Modified Film Technology Engineering of Henan Province, Xinxiang 453000, China
[3] Medical College, Xinxiang University, Xinxiang 453000, China; liyanli2005@126.com
* Correspondence: chenrx@xxu.edu.cn (R.C.); mxj022@163.com (X.M.)

Supporting Information

Table of Contents

1. General methods

2. General procedure for synthesis of 1,2,4-oxadiazolo[5,4-*a*]isoquinoline compounds

3. Crystal data and structural refinement for 3j

4. NMR spectra

1. General methods

The hydroximoyl chloride **1** [1–3] and isolable 2,4,6-trimethylbenzonitrile oxide **1a'** [4,5] were prepared according to the literature procedures. The cyclic imines **2** were prepared according to the literature procedures [6–8].

1. Schäfer, R.J.B.; Monaco, M.R.; Li, M.; Tirla, A.; Rivera-Fuentes, P.; Wennemers, H. The Bioorthogonal Isonitrile–Chlorooxime Ligation. *J. Am. Chem. Soc.* **2019**, *141*, 18644–18648.
2. Ong, M.J.H.; Hewitt, R.J. Synthesis of 1,4,2-Oxathiazoles via Norrish Type II Generation of Thiocarbonyls. *ChemistrySelect* **2019**, *4*, 10532–10535.
3. Vo, Q.V.; Trenerry, C.; Rochfort, S.; Wadeson, J.; Leyton, C.; Hughes, A.B. Synthesis and anti-inflammatory activity of aromatic glucosinolates. *Bioorg. Med. Chem.* **2013**, *21*, 5945–5954.
4. Zhao, G.; Liang, L.; Wen, C.H.E.; Tong, R. In Situ Generation of Nitrile Oxides from NaCl–Oxone Oxidation of Various Ald-oximes and Their 1,3-Dipolar Cycloaddition. *Org. Lett.* **2019**, *21*, 315–319.
5. Altintas, O.; Glassner, M.; Rodriguez-Emmenegger, C.; Welle, A.; Trouillet, V.; Barner-Kowollik, C. Macromolecular Surface Design: Photopatterning of Functional Stable Nitrile Oxides. *Angew. Chem. Int. Ed.* **2015**, *54*, 5777–5783.
6. Mbere, J.M.; Bremner, J.B.; Skelton, B.W.; White, A.H. Synthesis of new benzo[*b*]thieno fused ring systems via transition metal-mediated cyclisations. *Tetrahedron* **2011**, *67*, 6895–6900.
7. Jakubec, P.; Helliwell, M.; Dixon, D.J. Cyclic Imine Nitro-Mannich/Lactamization Cascades: A Direct Stereoselective Synthesis of Multicyclic Piperidinone Derivatives. *Org. Lett.* **2008**, *10*, 4267–4270.
8. Elliott, M.C.; Williams, E. Synthesis and reactions of partially reduced biisoquinolines. *Org. Biomol. Chem.* **2003**, *1*, 3038–3047.

2. General procedure for synthesis of 1,2,4-oxadiazolo[5,4-*a*]isoquinoline compounds

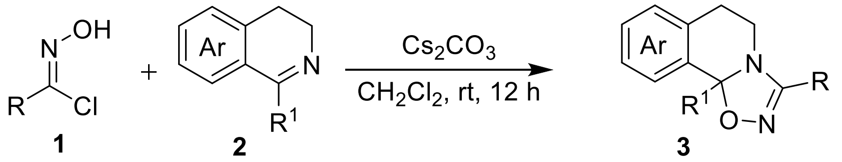

Cs_2CO_3 (0.22 mmol) was added to a solution of oxime chlorides **1** (0.22 mmol), cyclic imines **2** (0.2 mmol) in CH_2Cl_2 (1 mL). The solution was stirred at rt for 12 h. After completion, product **3** was obtained by flash chromatography on silica gel (petroleum ether/ethyl acetate = 15:1 to 10:1).

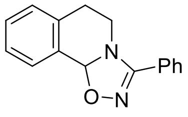

3a

3-Phenyl-6,10b-dihydro-5*H*-[1,2,4]oxadiazolo[5,4-*a*]isoquinoline 3a, 47.5 mg, 95% yield, white solid; ^{1}H NMR (400 MHz, $CDCl_3$) δ 7.70 (d, *J* = 7.6 Hz, 2H), 7.50–7.43 (m, 4H), 7.32–7.24 (m, 2H), 7.12 (d, *J* = 5.6 Hz, 1H), 6.51 (s, 1H), 3.48 (dt, *J* = 12.8, 4.4 Hz, 1H), 3.37–3.31 (m, 1H), 2.78 (ddd, *J* = 15.2, 10.0, 4.4 Hz, 1H), 2.63 (dt, *J* = 15.6, 3.6 Hz, 1H) ppm. ^{13}C NMR (100 MHz, $CDCl_3$) δ 159.6, 135.8, 131.7, 130.9, 128.9, 128.4, 128.3, 128.2, 127.2, 125.7, 92.0, 43.4, 28.2 ppm. ESI-HRMS: $C_{16}H_{14}N_2O+H^+$ 251.1179, found 251.1176.

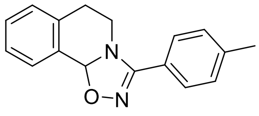

3b

3-(*p*-Tolyl)-6,10b-dihydro-5*H*-[1,2,4]oxadiazolo[5,4-*a*]isoquinoline 3b, 48.0 mg, 91% yield, white solid; ^{1}H NMR (400 MHz, $CDCl_3$) δ 7.59 (d, *J* = 8.0 Hz, 2H), 7.45 (d, *J* = 5.6 Hz, 1H), 7.34–7.31 (m, 2H), 7.27 (d, *J* = 8.4 Hz, 2H), 7.13 (d, *J* = 5.6 Hz, 1H), 6.51 (s, 1H), 3.49 (dt, *J* = 12.8, 4.4 Hz, 1H), 3.37–3.30 (m, 1H), 2.78 (ddd, *J* = 15.2, 10.4, 4.4 Hz, 1H), 2.63 (d, *J* = 15.6 Hz, 1H), 2.41 (s, 3H) ppm. ^{13}C NMR (100 MHz, $CDCl_3$) δ 159.6, 141.2, 135.9, 131.8, 129.6, 128.9, 128.4, 128.3, 128.2, 127.2, 122.7, 91.8, 43.4, 28.3, 21.5 ppm. ESI-HRMS: $C_{17}H_{16}N_2O+H^+$ 265.1335, found 265.1332.

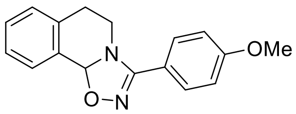

3c

3-(4-Methoxyphenyl)-6,10b-dihydro-5*H*-[1,2,4]oxadiazolo[5,4-*a*]isoquinoline 3c, 50.4 mg, 90% yield, white solid; ^{1}H NMR (400 MHz, $CDCl_3$) δ 7.65 (d, *J* = 8.4 Hz, 2H), 7.46–7.44 (m, 1H), 7.34–7.26 (m, 2H), 7.14–7.12 (m, 1H), 6.97 (d, *J* = 8.4 Hz, 2H), 6.46 (s, 1H),

3.85 (s, 3H), 3.46 (dt, *J* = 12.8, 4.4 Hz, 1H), 3.36–3.29 (m, 1H), 2.83–2.75 (m, 1H), 2.65 (dt, *J* = 15.6, 3.6 Hz, 1H) ppm. ^{13}C NMR (100 MHz, $CDCl_3$) δ 161.7, 159.5, 136.0, 131.7, 129.9, 128.9, 128.34, 128.32, 127.1, 117.9, 114.4, 91.7, 55.4, 43.6, 28.4 ppm. ESI-HRMS: $C_{17}H_{16}N_2O_2+H^+$ 281.1285, found 281.1281.

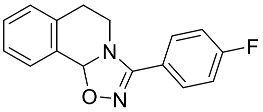

3d

3-(4-Fluorophenyl)-6,10b-dihydro-5*H*-[1,2,4]oxadiazolo[5,4-*a*]isoquinoline 3d, 50.9 mg, 95% yield, white solid; ^{1}H NMR (400 MHz, $CDCl_3$) δ 7.73–7.69 (m, 2H), 7.45 (d, *J* = 4.4 Hz, 1H), 7.34–7.32 (m, 2H), 7.18–7.14 (m, 3H), 6.50 (s, 1H), 3.47–3.30 (m, 2H), 2.82 -2.64 (m, 2H) ppm. ^{13}C NMR (100 MHz, $CDCl_3$) δ 164.2 (d, *J* = 250.0 Hz), 158.9, 135.8, 131.4, 130.36 (d, *J* = 8.6 Hz), 129.0, 128.4, 128.3, 127.2, 121.8 (d, *J* = 3.3 Hz), 116.2 (d, *J* = 21.8 Hz), 92.0, 43.5, 28.3 ppm. ESI-HRMS: $C_{16}H_{13}FN_2O+H^+$ 269.1085, found 269.1083.

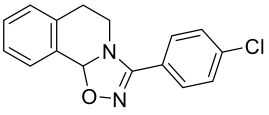

3e

3-(4-Chlorophenyl)-6,10b-dihydro-5*H*-[1,2,4]oxadiazolo[5,4-*a*]isoquinoline 3e, 53.4 mg, 94% yield, white solid; ^{1}H NMR (400 MHz, $CDCl_3$) δ 7.66 (d, *J* = 8.4 Hz, 2H), 7.44 (d, *J* = 7.6 Hz, 3H), 7.35–7.30 (m, 2H), 7.15 (d, *J* = 4.8 Hz, 1H), 6.50 (s, 1H), 3.43 (dt, *J* = 12.8, 4.4 Hz, 1H), 3.37–3.30 (m, 1H), 2.79 (ddd, *J* = 15.2, 10.0, 4.4 Hz, 1H), 2.66 (dt, *J* = 15.6, 3.6 Hz, 1H) ppm. ^{13}C NMR (100 MHz, $CDCl_3$) δ 158.9, 137.0, 135.8, 131.3, 129.5, 129.3, 129.0, 128.4, 128.3, 127.2, 124.2, 92.1, 43.5, 28.3 ppm. ESI-HRMS: $C_{16}H_{13}ClN_2O+H^+$ 285.0789, found 285.0786.

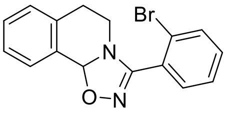

3f

3-(2-Bromophenyl)-6,10b-dihydro-5*H*-[1,2,4]oxadiazolo[5,4-*a*]isoquinoline 3f, 60.4 mg, 92% yield, white solid; ^{1}H NMR (400 MHz, $CDCl_3$) δ 7.69 (d, *J* = 8.0 Hz, 1H), 7.50 (dd, *J* = 7.6, 1.6 Hz, 1H), 7.45 (d, *J* = 6.8 Hz, 1H), 7.42–7.45 (m, 2H), 7.33–7.28 (m, 2H), 7.10 (d, *J* = 6.8 Hz, 1H), 6.72 (s, 1H), 3.40–3.28 (m, 2H), 2.90–2.81 (m, 1H), 2.58 (d, *J* = 16.0 Hz, 1H) ppm. ^{13}C NMR (100 MHz, $CDCl_3$) δ 157.4, 134.9, 133.8, 133.0, 131.97, 131.91, 128.7, 128.6, 127.6, 127.3, 126.8, 122.9, 91.6, 41.8, 27.9 ppm. ESI-HRMS: $C_{16}H_{13}BrN_2O+H^+$ 329.0284 (^{79}Br) and 331.0264 (^{81}Br), found 329.0283 and 331.0261.

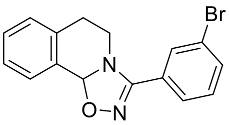

3g

3-(3-Bromophenyl)-6,10b-dihydro-5*H*-[1,2,4]oxadiazolo[5,4-*a*]isoquinoline 3g, 61.7 mg, 94% yield, white solid; ^{1}H NMR (400 MHz, $CDCl_3$) δ 7.87 (s, 1H), 7.66–7.61 (m, 2H), 7.46–7.44 (m, 1H), 7.36–7.31 (m, 3H), 7.16–7.14 (m, 1H), 6.51 (s, 1H), 3.45 (dt, *J* = 12.8, 4.4 Hz, 1H), 3.38–3.31 (m, 1H), 2.80 (ddd, *J* = 15.2, 10.0, 4.4 Hz, 1H), 2.67 (dt, *J* = 16.0, 3.6 Hz, 1H) ppm. ^{13}C NMR (100 MHz, $CDCl_3$) δ 158.5, 135.7, 133.9, 131.2, 131.1, 130.5, 129.1, 128.4, 128.3, 127.7, 127.3, 126.8, 122.9, 92.2, 43.6, 28.3 ppm. ESI-HRMS: $C_{16}H_{13}BrN_2O+H^+$ 329.0284 (^{79}Br) and 331.0264 (^{81}Br), found 329.0282 and 331.0260.

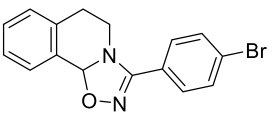

3h

3-(4-Bromophenyl)-6,10b-dihydro-5*H*-[1,2,4]oxadiazolo[5,4-*a*]isoquinoline 3h, 62.3mg, 95% yield, white solid; ^{1}H NMR (400 MHz, $CDCl_3$) δ 7.61–7.57 (m, 4H), 7.44 (d, *J* = 4.4 Hz, 1H), 7.32 (dd, *J* = 4.4, 2.8 Hz, 2H), 7.14 (d, *J* = 4.4 Hz, 1H), 6.49 (s, 1H), 3.45–3.39 (m, 1H), 3.36–3.30 (m, 1H), 2.82–2.74 (m, 1H), 2.66 (d, *J* = 16.0 Hz, 1H) ppm. ^{13}C NMR (100 MHz, $CDCl_3$) δ 158.9, 135.7, 132.2, 131.3, 129.7, 129.1, 128.4, 128.3, 127.2, 125.3, 124.7, 92.2, 43.6, 28.3 ppm. ESI-HRMS: $C_{16}H_{13}BrN_2O+H^+$ 329.0284 (^{79}Br) and 331.0264 (^{81}Br), found 329.0281 and 331.0260.

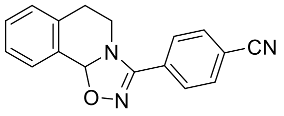

3i

4-(6,10b-Dihydro-5*H*-[1,2,4]oxadiazolo[5,4-*a*]isoquinolin-3-yl)benzonitrile 3i, 52.8 mg, 96% yield, white solid; ^{1}H NMR (400 MHz, $CDCl_3$) δ 7.85 (d, *J* = 8.0 Hz, 2H), 7.77 (d, *J* = 8.4 Hz, 2H), 7.47–7.45 (m, 1H), 7.35 (dd, *J* = 5.6, 3.6 Hz, 2H), 7.18–7.15 (m, 1H), 6.53 (s, 1H), 3.43–3.33 (m, 2H), 2.84–2.67 (m, 2H) ppm. ^{13}C NMR (100 MHz, $CDCl_3$) δ 158.5, 135.6, 132.7, 130.8, 130.2, 129.3, 128.7, 128.4, 128.3, 127.4, 118.1, 114.4, 92.7, 43.7, 28.3 ppm. ESI-HRMS: $C_{17}H_{13}N_3O+H^+$ 276.1131, found 276.1130.

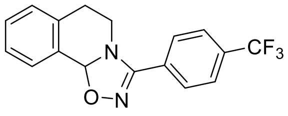

3j

3-(4-(Trifluoromethyl)phenyl)-6,10b-dihydro-5*H*-[1,2,4]oxadiazolo[5,4-*a*]isoquinoline 3j, 61.1 mg, 96% yield, white solid; ^{1}H NMR (400 MHz, $CDCl_3$) δ 7.85 (d, *J* = 8.4 Hz, 2H), 7.73 (d, *J* = 8.0 Hz, 2H), 7.47–7.45 (m, 1H), 7.36–7.31 (m, 2H), 7.16–7.14 (m, 1H), 6.54 (s, 1H), 3.46–3.33 (m, 2H), 2.79 (ddd, *J* = 15.2, 9.6, 4.4 Hz, 1H), 2.68 (dt, *J* = 16.0, 4.0 Hz, 1H) ppm. ^{13}C NMR (100 MHz, $CDCl_3$) δ 158.7, 135.7, 132.7 (q, *J* = 32.5 Hz), 131.2, 129.4, 129.2, 128.6, 128.4, 128.3, 127.3, 125.9 (q, *J* = 3.7 Hz), 123.7 (q, *J* = 268.8 Hz), 92.5, 43.6, 28.3 ppm. ESI-HRMS: $C_{17}H_{13}F_3N_2O+H^+$ 319.1053, found 319.1051.

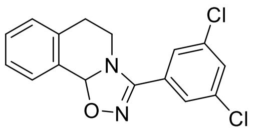

3k

3-(3,5-Dichlorophenyl)-6,10b-dihydro-5*H*-[1,2,4]oxadiazolo[5,4-*a*]isoquinoline 3k, 59.8 mg, 94% yield, white solid; ^{1}H NMR (400 MHz, $CDCl_3$) δ 7.61 (s, 2H), 7.48 (s, 1H), 7.44 (d, *J* = 4.4 Hz, 1H), 7.33 (dd, *J* = 7.2, 4.0 Hz, 2H), 7.16 (d, *J* = 3.6 Hz, 1H), 6.49 (s, 1H), 3.43–3.31 (m, 2H), 2.84–2.77 (m, 1H), 2.74–2.69 (m, 1H) ppm. ^{13}C NMR (100 MHz, $CDCl_3$) δ 157.9, 135.73, 135.67, 130.84, 130.82, 129.2, 128.7, 128.4, 128.3, 127.3, 126.5, 92.6, 43.7, 28.3 ppm. ESI-HRMS: $C_{16}H_{12}Cl_2N_2O+H^+$ 319.0399, found 319.0397.

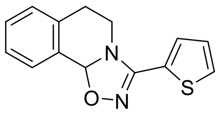

3l

3-(Thiophen-2-yl)-6,10b-dihydro-5*H*-[1,2,4]oxadiazolo[5,4-*a*]isoquinoline 3l, 46.1 mg, 90% yield, white solid; ^{1}H NMR (400 MHz, $CDCl_3$) δ 7.45–7.43 (m, 1H), 7.33 (dd, *J* = 8.4, 4.0 Hz, 2H), 7.26 (d, *J* = 4.4 Hz, 2H), 7.17–7.15 (m, 1H), 6.96 (d, *J* = 3.6 Hz, 1H), 6.42 (s, 1H), 3.60–3.55 (m, 1H), 3.42–3.35 (m, 1H), 2.90–2.83 (m, 1H), 2.76–2.70 (m, 1H) ppm. ^{13}C NMR (100 MHz, $CDCl_3$) δ 154.5, 135.8, 133.9, 130.8, 129.2, 128.7, 128.3, 127.2, 126.9, 125.6, 92.2, 44.1, 28.4 ppm. ESI-HRMS: $C_{14}H_{12}N_2OS+H^+$ 257.0743, found 257.0738.

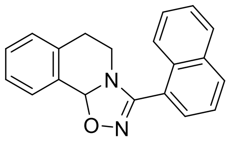

3m

3-(Naphthalen-1-yl)-6,10b-dihydro-5*H*-[1,2,4]oxadiazolo[5,4-*a*]isoquinoline 3m, 54.6 mg, 91% yield, white solid; ^{1}H NMR (400 MHz, $CDCl_3$) δ 8.39 (d, *J* = 8.8 Hz, 1H), 7.98 (d, *J* = 8.0 Hz, 1H), 7.91–7.89 (m, 1H), 7.73 (d, *J* = 6.8 Hz, 1H), 7.56–7.52(m, 4H), 7.37–7.29 (m, 2H), 7.07 (d, *J* = 7.2 Hz, 1H), 6.79 (s, 1H), 3.32–3.28 (m, 2H), 2.78–2.70 (m, 1H), 2.49 (d, *J* = 16.0 Hz, 1H) ppm. ^{13}C NMR (100 MHz, $CDCl_3$) δ 158.0, 135.1, 133.6, 133.1, 131.24, 131.19, 128.7, 128.6, 128.53, 128.46, 127.8, 127.34, 127.32, 126.5, 125.2, 122.5, 91.6, 42.3, 28.0 ppm. ESI-HRMS: $C_{20}H_{16}N_2O+H^+$ 301.1335, found 301.1332.

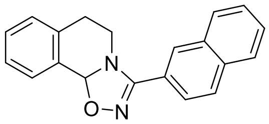

3n

3-(Naphthalen-2-yl)-6,10b-dihydro-5*H*-[1,2,4]oxadiazolo[5,4-*a*]isoquinoline 3n, 54.0 mg, 90% yield, white solid; ^{1}H NMR (400 MHz, $CDCl_3$) δ 8.21 (s, 1H), 7.93–7.87 (m, 3H), 7.79 (d, *J* = 8.4 Hz, 1H), 7.58–7.52 (m, 3H), 7.36–7.30 (m, 2H), 7.14 (d, *J* = 6.4 Hz, 1H), 6.56 (s,

1H), 3.55 (dt, J = 12.8, 4.4 Hz, 1H), 3.44–3.38 (m, 1H), 2.85–2.78 (m, 1H), 2.66 (d, J = 15.6 Hz, 1H) ppm. ^{13}C NMR (100 MHz, $CDCl_3$) δ 159.8, 135.9, 134.4, 132.9, 131.5, 129.0, 128.8, 128.54, 128.46, 128.4, 128.3, 127.8, 127.5, 127.2, 126.8, 124.8, 123.0, 92.1, 43.7, 28.3 ppm. ESI-HRMS: $C_{20}H_{16}N_2O$+H^+ 301.1335, found 301.1334.

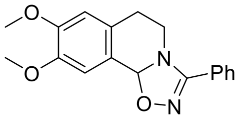

3o

8,9-Dimethoxy-3-phenyl-6,10b-dihydro-5*H*-[1,2,4]oxadiazolo[5,4-*a*]isoquinoline 3o, 58.9 mg, 95% yield, white solid; ^{1}H NMR (400 MHz, $CDCl_3$) δ 7.67 (d, J = 6.4 Hz, 2H), 7.46 (d, J = 6.8 Hz, 3H), 6.92 (s, 1H), 6.59 (s, 1H), 6.50 (s, 1H), 3.92 (s, 3H), 3.86 (s, 3H), 3.51 (d, J = 13.2 Hz, 1H), 3.34 (t, J = 8.8 Hz, 1H), 2.73–2.66 (m, 1H), 2.53 (d, J = 15.6 Hz, 1H) ppm. ^{13}C NMR (100 MHz, $CDCl_3$) δ 159.4, 149.3, 148.2, 130.8, 128.9, 128.2, 128.0, 125.6, 123.9, 111.0, 110.3, 91.9, 56.0, 55.9, 43.2, 27.6 ppm. ESI-HRMS: $C_{18}H_{18}N_2O_3$+H^+ 311.1390, found 311.1386.

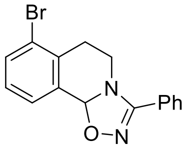

3p

7-Bromo-3-phenyl-6,10b-dihydro-5*H*-[1,2,4]oxadiazolo[5,4-*a*]isoquinoline 3p, 59.0 mg, 90% yield, white solid; ^{1}H NMR (400 MHz, $CDCl_3$) δ 7.66 (d, J = 7.2 Hz, 2H), 7.57 (d, J = 7.6 Hz, 1H), 7.52–7.45 (m, 3H), 7.42 (d, J = 7.6 Hz, 1H), 7.21 (t, J = 8.0 Hz, 1H), 6.52 (s, 1H), 3.59 (dt, J = 13.6, 4.4 Hz, 1H), 3.39–3.33 (m, 1H), 2.80 (d, J = 16.8 Hz, 1H), 2.67–2.59 (m, 1H) ppm. ^{13}C NMR (100 MHz, $CDCl_3$) δ 159.1, 135.2, 134.9, 132.8, 131.0, 129.0, 128.4, 128.2, 127.2, 125.2, 124.4, 91.6, 42.5, 27.9 ppm. ESI-HRMS: $C_{16}H_{13}BrN_2O$+H^+ 329.0284 (^{79}Br) and 331.0264 (^{81}Br), found 329.0281 and 331.0260.

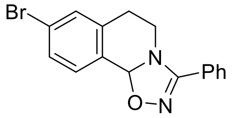

3q

8-Bromo-3-phenyl-6,10b-dihydro-5*H*-[1,2,4]oxadiazolo[5,4-*a*]isoquinoline 3q, 59.7 mg, 91% yield, white solid; ^{1}H NMR (400 MHz, $CDCl_3$) δ 7.69–7.67 (m, 2H), 7.53–7.45 (m, 4H), 7.33–7.30 (m, 2H), 6.48 (s, 1H), 3.49 (dt, J = 13.2, 4.4 Hz, 1H), 3.37–3.30 (m, 1H), 2.75 (ddd, J = 15.6, 10.4, 4.4 Hz, 1H), 2.60 (dt, J = 15.6, 3.6 Hz, 1H) ppm. ^{13}C NMR (100 MHz, $CDCl_3$) δ 159.5, 138.0, 131.3, 131.0, 130.9, 130.4, 129.8, 129.0, 128.2, 125.3, 123.0, 91.4, 43.0, 27.9 ppm. ESI-HRMS: $C_{16}H_{13}BrN_2O$+H^+ 329.0284 (^{79}Br) and 331.0264 (^{81}Br), found 2329.0280 and 331.0258.

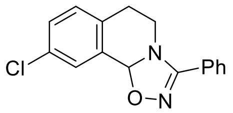

3r

9-Chloro-3-phenyl-6,10b-dihydro-5*H*-[1,2,4]oxadiazolo[5,4-*a*]isoquinoline 3r, 50.0 mg, 88% yield, white solid; ^{1}H NMR (400 MHz, $CDCl_3$) δ 7.68 (d, J = 7.6 Hz, 2H), 7.52–7.45 (m, 4H), 7.29–7.26 (m, 1H), 7.06 (d, J = 8.0 Hz, 1H), 6.48 (s, 1H), 3.51 (dt, J = 13.2, 4.4 Hz, 1H), 3.39–3.32 (m, 1H), 2.72 (ddd, J = 15.2, 10.0, 4.4 Hz, 1H), 2.60 (dt, J = 15.2, 3.6 Hz, 1H) ppm. ^{13}C NMR (100 MHz, $CDCl_3$) δ 159.4, 134.0, 133.8, 132.9, 131.0, 129.8, 129.03, 129.0, 128.3, 128.1, 125.4, 91.3, 43.1, 27.6 ppm. ESI-HRMS: $C_{16}H_{13}ClN_2O$+H^+ 285.0789, found 285.0786.

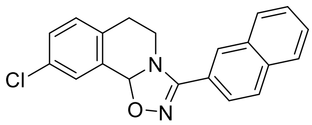

3s

9-Chloro-3-(naphthalen-2-yl)-6,10b-dihydro-5*H*-[1,2,4]oxadiazolo[5,4-*a*]isoquinoline 3s, 60.8 mg, 91% yield, white solid; ^{1}H NMR (400 MHz, $CDCl_3$) δ 8.18 (s, 1H), 7.93–7.87 (m, 3H), 7.75 (d, J = 8.4 Hz, 1H), 7.59–7.53 (m, 2H), 7.47 (s, 1H), 7.28 (d, J = 8.4 Hz, 1H), 7.06 (d, J = 8.0 Hz, 1H), 6.51 (s, 1H), 3.56 (dt, J = 12.8, 4.4 Hz, 1H), 3.44–3.37 (m, 1H), 2.74 (ddd, J = 15.2, 10.0, 4.4 Hz, 1H), 2.64–2.59 (m, 1H) ppm. ^{13}C NMR (100 MHz, $CDCl_3$) δ 159.6, 134.4, 134.1, 133.6, 132.9, 132.8, 129.8, 129.1, 128.9, 128.53, 128.47, 128.1, 127.9, 127.6, 126.9, 124.7, 122.7, 91.5, 43.3, 27.6 ppm. ESI-HRMS: $C_{20}H_{15}ClN_2O$+H^+ 335.0946, found 335.0941.

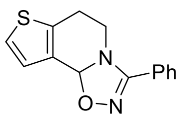
3t

3-Phenyl-6,9b-dihydro-5*H*-[1,2,4]oxadiazolo[4,5-*a*]thieno[3,2-*c*]pyridine 3t, 47.6 mg, 93% yield, white solid; ^{1}H NMR (400 MHz, $CDCl_3$) δ 7.61 (d, *J* = 7.2 Hz, 2H), 7.51–7.44 (m, 3H), 7.19 (d, *J* = 5.2 Hz, 1H), 7.07 (d, *J* = 5.2 Hz, 1H), 6.63 (s, 1H), 3.69 (dt, *J* = 13.6, 3.6 Hz, 1H), 3.47–3.40 (m, 1H), 2.75–2.63 (m, 2H) ppm. ^{13}C NMR (100 MHz, $CDCl_3$) δ 158.7, 137.1, 132.2, 130.8, 128.9, 128.2, 125.5, 125.4, 124.1, 90.2, 42.6, 24.0 ppm. ESI-HRMS: $C_{14}H_{12}N_2OS+H^+$ 257.0743, found 257.0740.

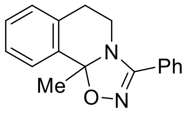
3u

10b-Methyl-3-phenyl-6,10b-dihydro-5*H*-[1,2,4]oxadiazolo[5,4-*a*]isoquinoline 3u, 50.7 mg, 96% yield, white solid; ^{1}H NMR (400 MHz, $CDCl_3$) δ 7.62 (d, *J* = 7.2 Hz, 2H), 7.51 (d, *J* = 7.6 Hz, 1H), 7.46–7.41 (m, 3H), 7.30 (t, *J* = 7.2 Hz, 1H), 7.25–7.21 (m, 1H), 7.03 (d, *J* = 7.6 Hz, 1H), 3.49–3.40 (m, 2H), 2.73–2.56 (m, 2H), 1.89 (s, 3H) ppm. ^{13}C NMR (100 MHz, $CDCl_3$) δ 158.4, 137.3, 133.6, 130.6, 128.8, 128.2, 128.1, 127.2, 126.4, 126.0, 97.2, 42.2, 27.95, 27.88 ppm. ESI-HRMS: $C_{17}H_{16}N_2O+H^+$ 265.1335, found 265.1333.

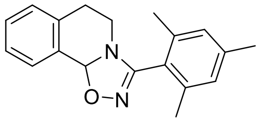
3v

3-Mesityl-6,10b-dihydro-5*H*-[1,2,4]oxadiazolo[5,4-*a*]isoquinoline 3v, 54.3 mg, 93% yield, white solid; ^{1}H NMR (400 MHz, $CDCl_3$) δ 7.50–7.48 (m, 1H), 7.35–7.27 (m, 2H), 7.10 (d, *J* = 7.2 Hz, 1H), 6.93 (s, 2H), 6.65 (s, 1H), 3.30 (ddd, *J* = 13.6, 11.2, 4.4 Hz, 1H), 3.17 (ddd, *J* = 12.8, 5.6, 2.8 Hz, 1H), 2.78 (ddd, *J* = 16.0, 10.8, 5.6 Hz, 1H), 2.60 (dt, *J* = 16.0, 3.6 Hz, 1H), 2.37 (s, 3H), 2.31 (s, 3H), 2.21 (s, 3H) ppm. ^{13}C NMR (100 MHz, $CDCl_3$) δ 156.3, 139.9, 138.5, 138.1, 134.4, 134.2, 128.8, 128.61, 128.59, 127.3, 127.2, 121.1, 90.1, 40.6, 28.0, 21.2, 20.3, 19.7 ppm. ESI-HRMS: $C_{19}H_{20}N_2O+H^+$ 293.1648, found 293.1647.

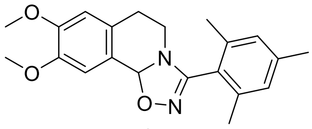
3w

3-Mesityl-8,9-dimethoxy-6,10b-dihydro-5*H*-[1,2,4]oxadiazolo[5,4-*a*]isoquinoline 3w, 66.9 mg, 95% yield, white solid; ^{1}H NMR (400 MHz, $CDCl_3$) δ 6.95 (s, 1H), 6.92 (s, 2H), 6.61 (s, 1H), 6.54 (s, 1H), 3.92 (s, 3H), 3.86 (s, 3H), 3.29 (ddd, *J* = 13.6, 11.2, 4.4 Hz, 1H), 3.18 (ddd, *J* = 16.0, 5.6, 2.4 Hz, 1H), 2.69 (ddd, *J* = 16.4, 11.6, 6.0 Hz, 1H), 2.47 (dd, *J* = 12.8, 3.2 Hz, 1H), 2.35 (s, 3H), 2.30 (s, 3H), 2.22 (s, 3H) ppm. ^{13}C NMR (100 MHz, $CDCl_3$) δ 156.2, 149.2, 148.3, 139.8, 138.5, 138.0, 128.9, 128.7, 126.5, 126.3, 121.0, 111.0, 109.4, 90.1, 56.0, 55.9, 40.5, 27.3, 21.2, 20.3, 19.6 ppm. ESI-HRMS: $C_{21}H_{24}N_2O_3+H^+$ 353.1860, found 353.1858.

3. Crystal data and structural refinement for 3j

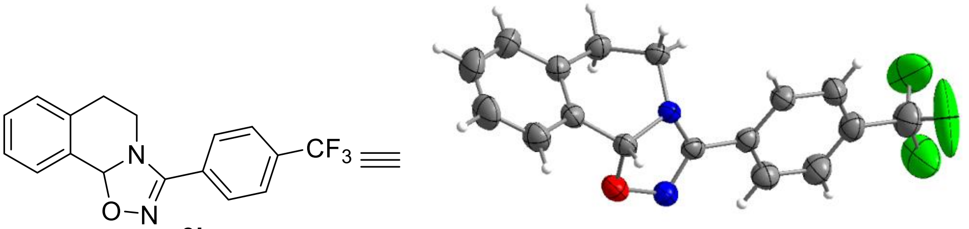

3j

CCDC 2160406

Identification code	3j
Empirical formula	$C_{17}H_{13}F_3N_2O$
Formula weight	318.29
Temperature/K	296
Crystal system	monoclinic
Space group	P 21/c
a/Å	15.121(3)
b/Å	8.7521(18)
c/Å	11.671(2)
α/°	90
β/°	110.785(3)
γ/°	90
Volume/Å^3	1444.0(5)
Z	4
ρ_{calc}g/cm^3	1.464
μ/mm^{-1}	0.119
F(000)	656.0
Crystal size/mm^3	0.26 × 0.25 × 0.2
Radiation	MoKα (λ = 0.71073)
2Θ range for data collection/°	2.737 to 24.998
Index ranges	−17 ≤ h ≤ 17, −8 ≤ k ≤ 10, −13 ≤ l ≤ 13
Reflections collected	7110
Independent reflections	1918 [R_{int} = 0.0240]
Data/restraints/parameters	2535 / 3 / 237
Goodness-of-fit on F^2	0.992
Final R indexes [I >= 2σ (I)]	R_1 = 0.0377, wR_2 = 0.1075
R indices (all data)	R_1 = 0.0511, wR_2 = 0.1160
Largest diff. peak and hole/ 1−sigma level	0.155 /−0.133/0.031

4. NMR spectra

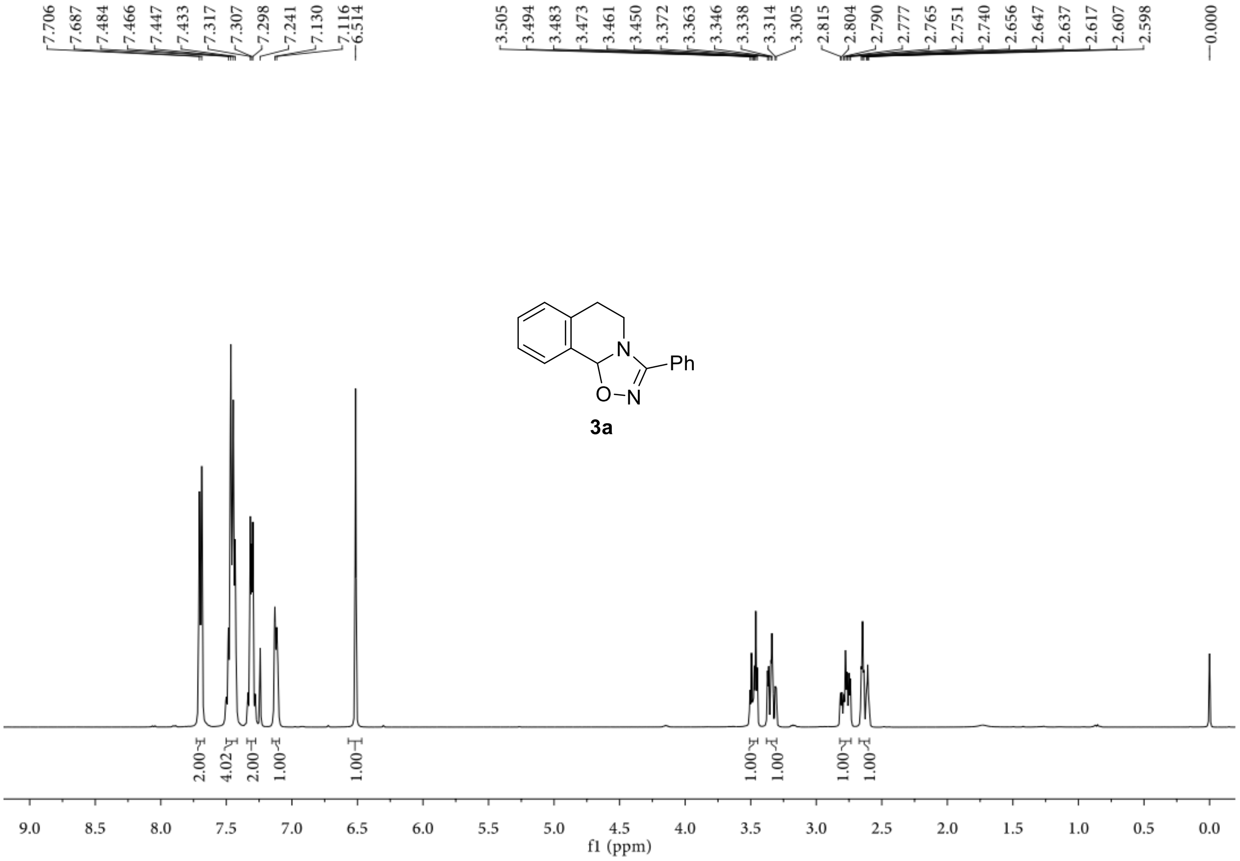

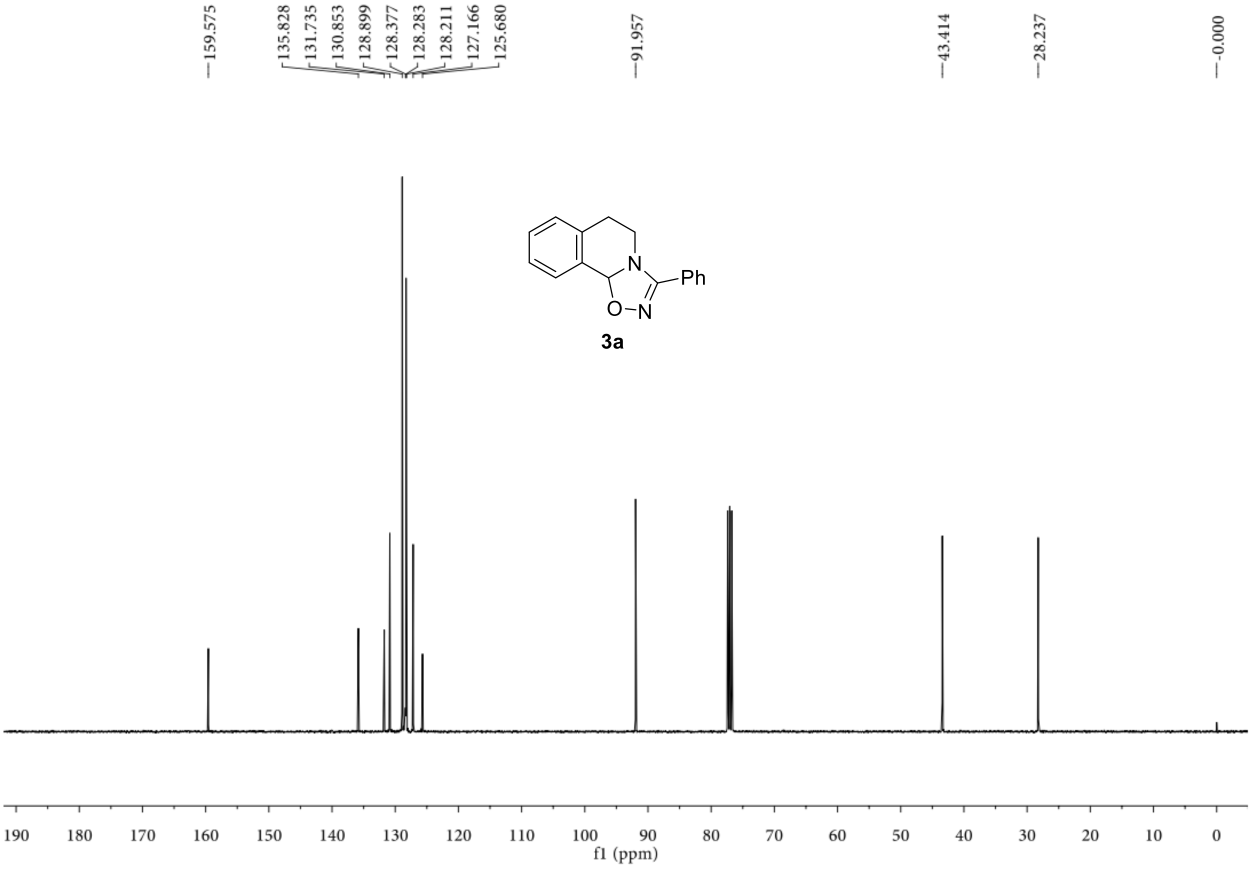

^{13}C-NMR DEPT 135°

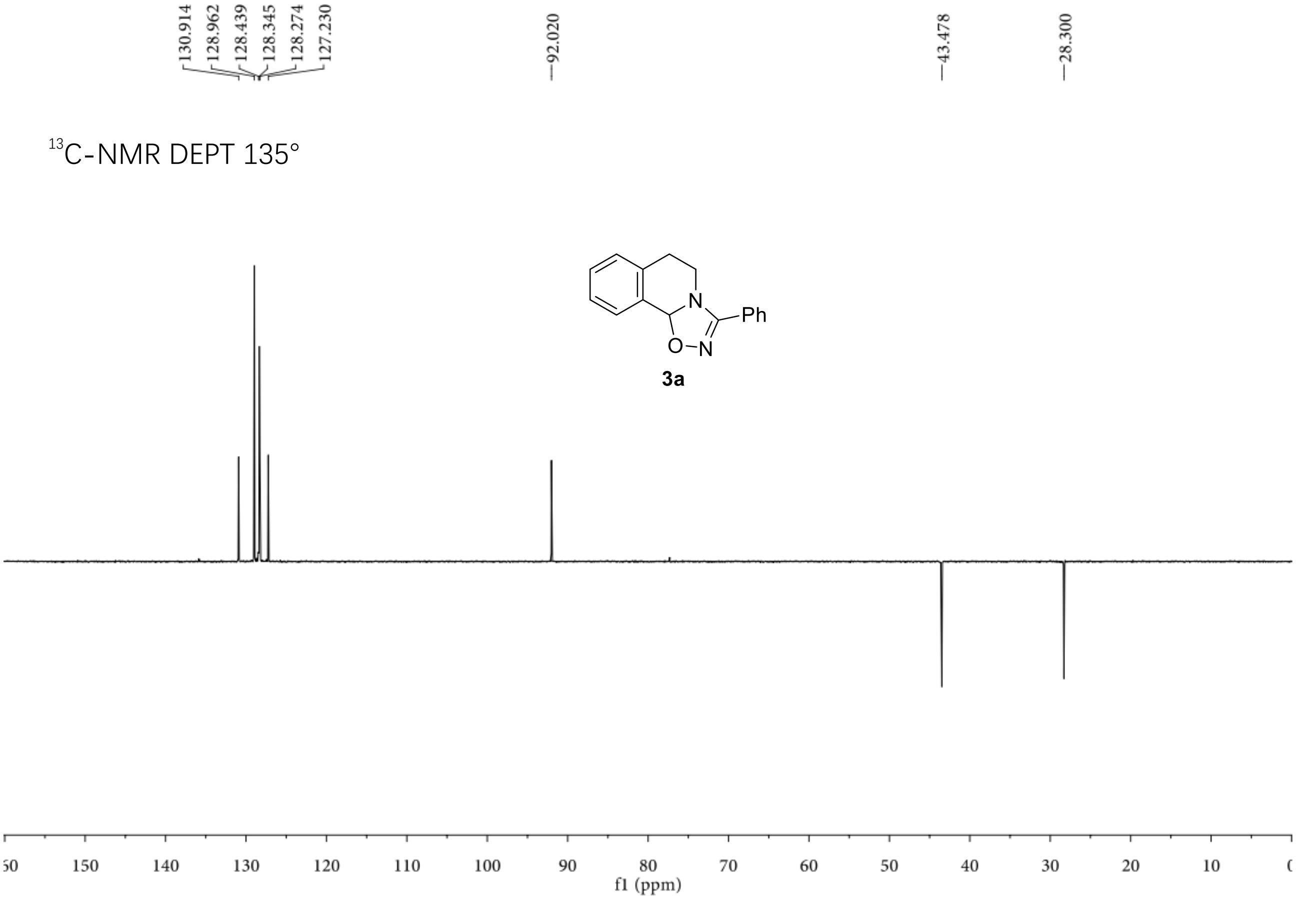

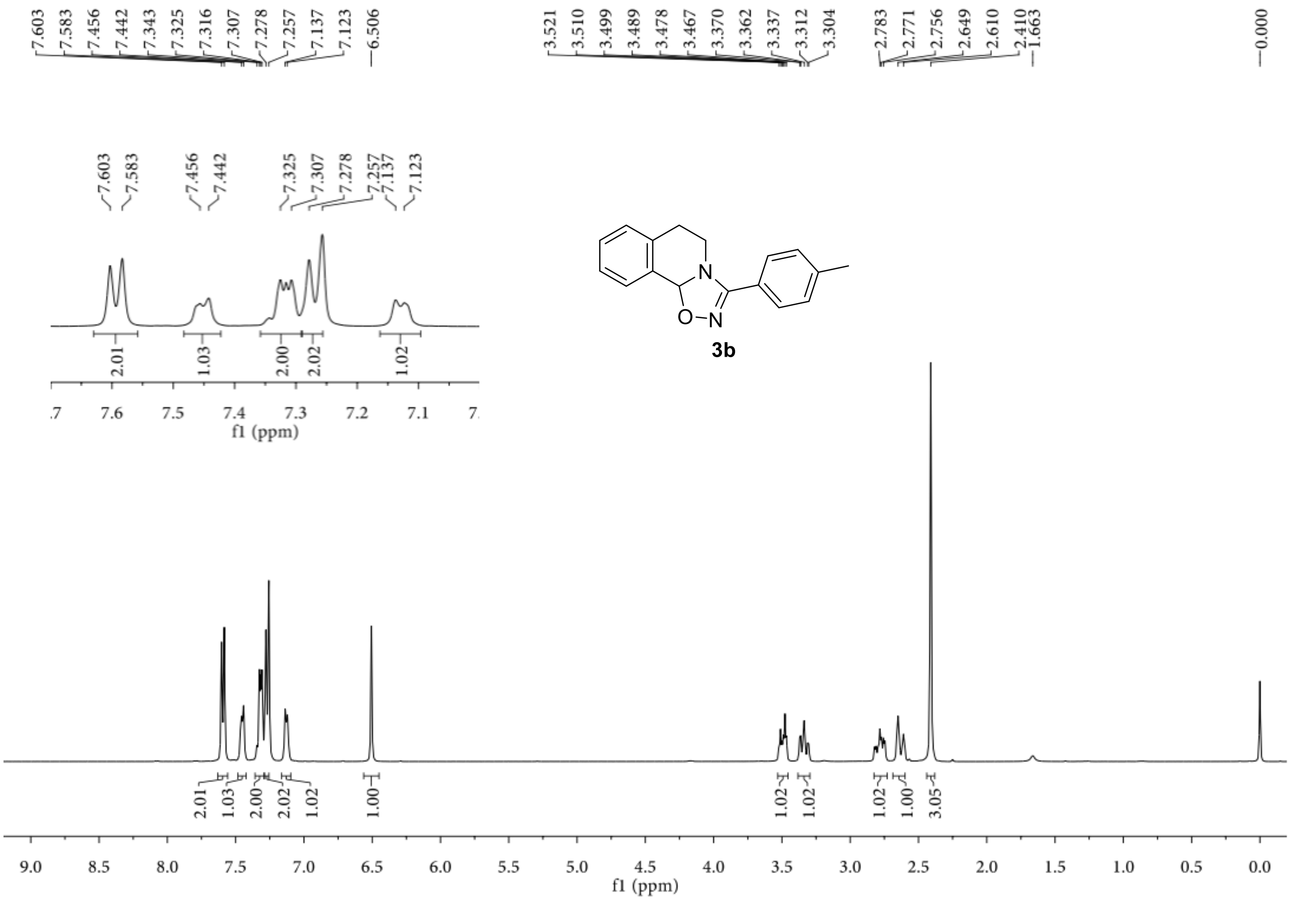
7.603 7.583 7.456 7.442 7.343 7.325 7.316 7.307 7.278 7.257 7.137 7.123
6.506
3.521 3.510 3.499 3.489 3.478 3.467 3.370 3.362 3.337 3.312 3.304
2.783 2.771 2.756 2.649 2.610 2.410 1.663
0.000
3b
2.01 1.03 2.00 2.02 1.02 1.00 1.02 1.02 1.02 1.00 3.05
f1 (ppm)

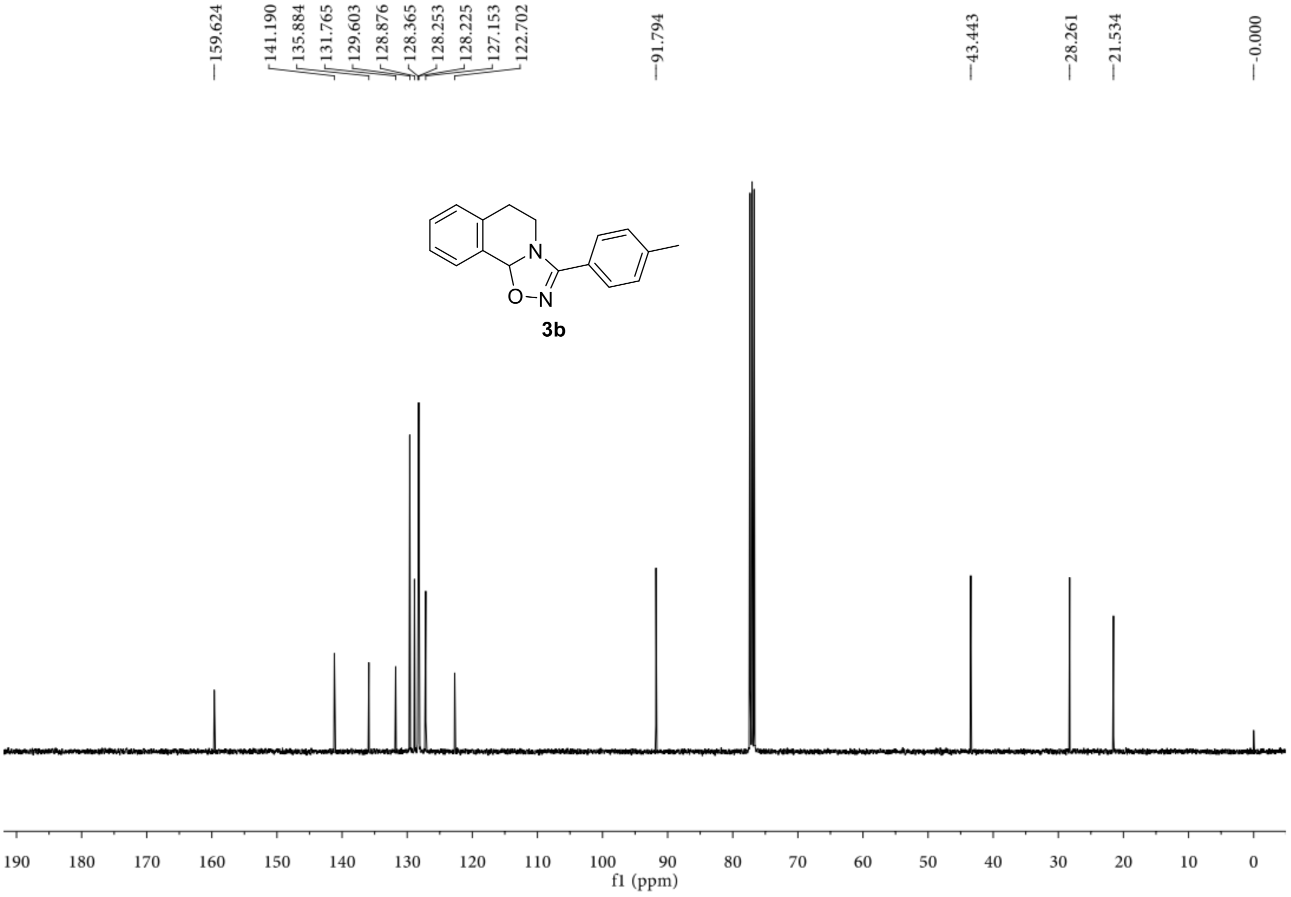
159.624
141.190 135.884 131.765 129.603 128.876 128.365 128.253 128.225 127.153 122.702
91.794
43.443
28.261
21.534
-0.000
3b
f1 (ppm)

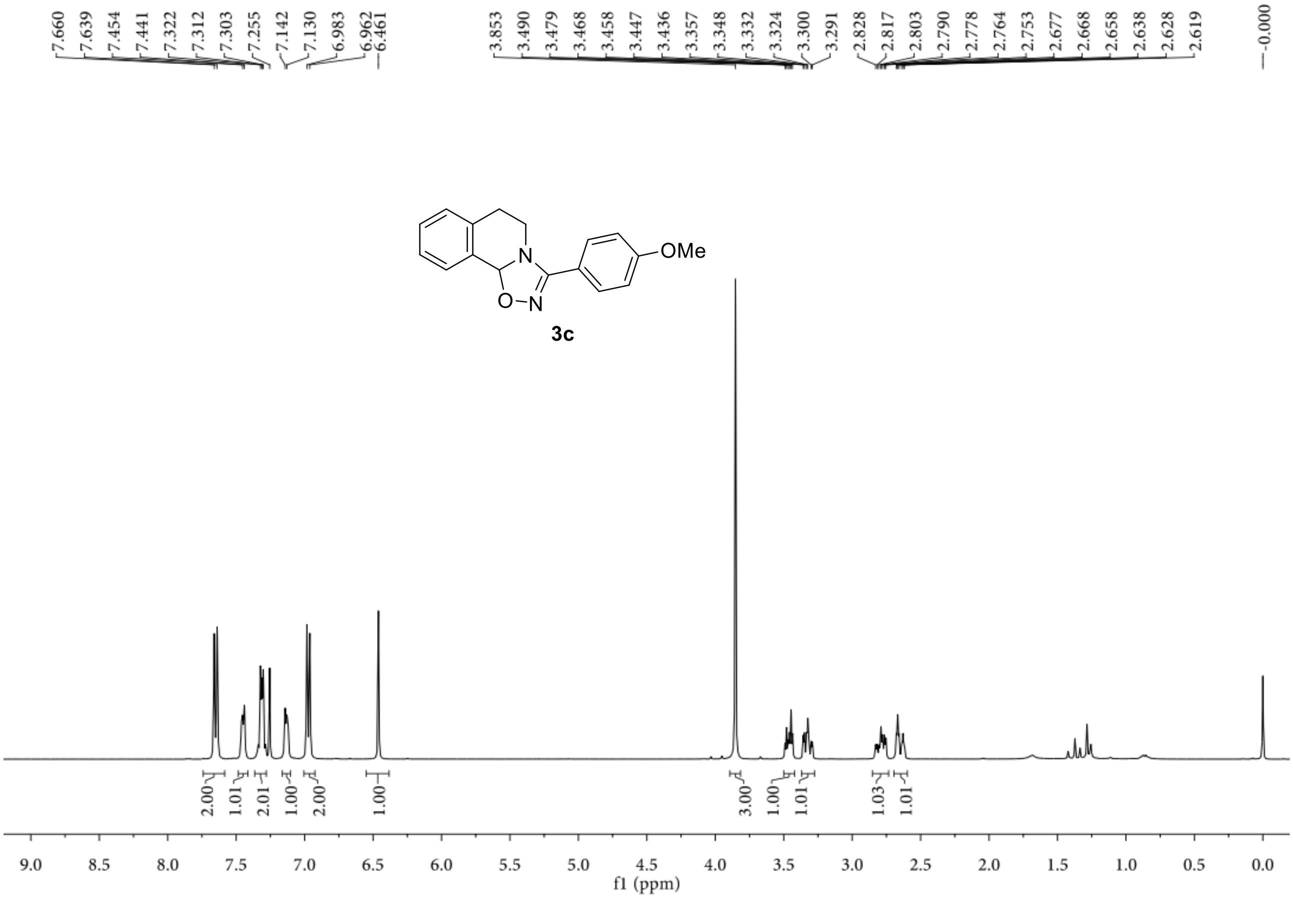
7.660
7.639
7.454
7.441
7.322
7.312
7.303
7.255
7.142
7.130
6.983
6.962
6.461
3.853
3.490
3.479
3.468
3.458
3.447
3.436
3.357
3.348
3.332
3.324
3.300
3.291
2.828
2.817
2.803
2.790
2.778
2.764
2.753
2.677
2.668
2.658
2.638
2.628
2.619
-0.000
OMe
N
O-N
3c
2.00
1.01
2.01
1.00
2.00
1.00
3.00
1.00
1.01
1.03
1.01
9.0 8.5 8.0 7.5 7.0 6.5 6.0 5.5 5.0 4.5 4.0 3.5 3.0 2.5 2.0 1.5 1.0 0.5 0.0
f1 (ppm)

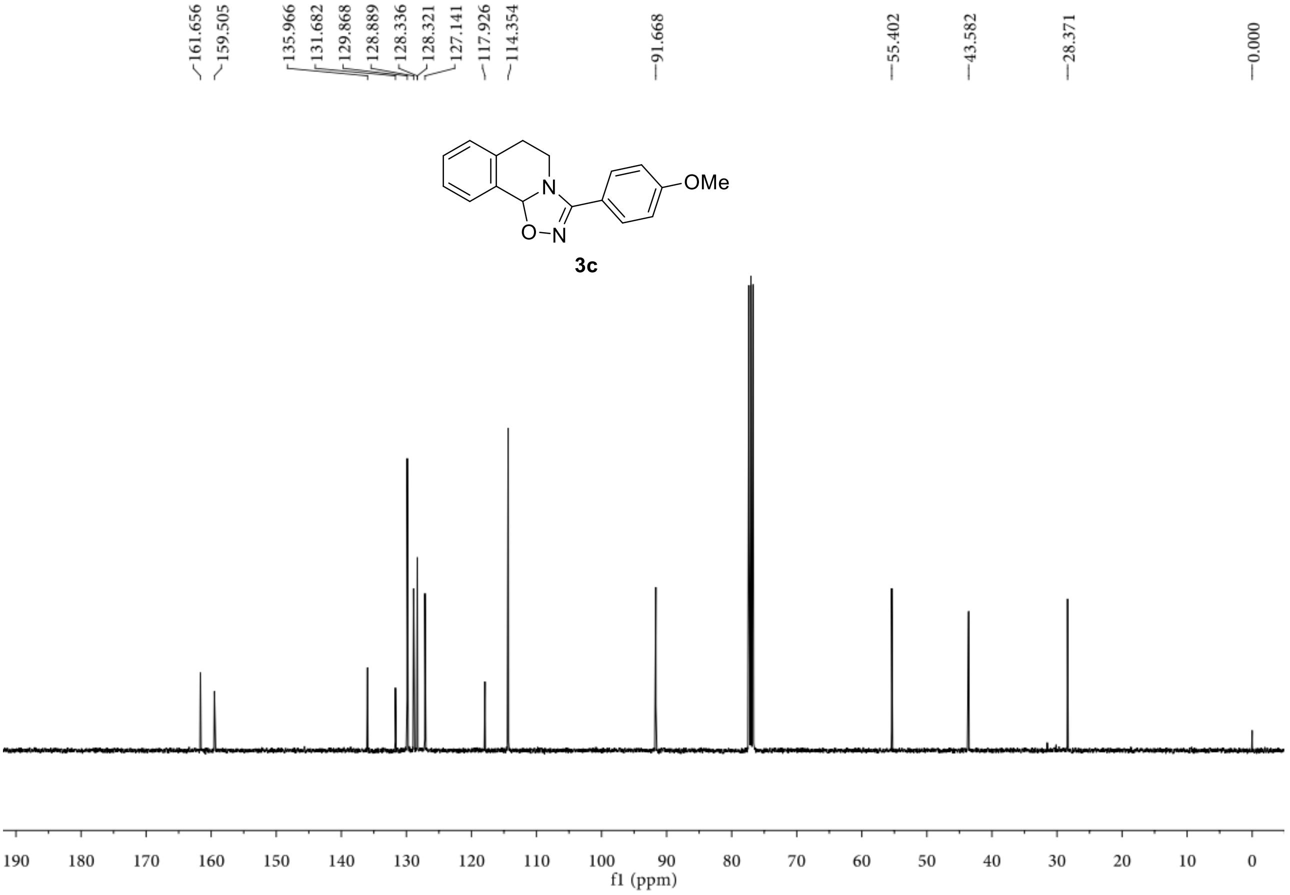
161.656
159.505
135.966
131.682
129.868
128.889
128.336
128.321
127.141
117.926
114.354
91.668
55.402
43.582
28.371
0.000
OMe
N
O-N
3c
190 180 170 160 150 140 130 120 110 100 90 80 70 60 50 40 30 20 10 0
f1 (ppm)

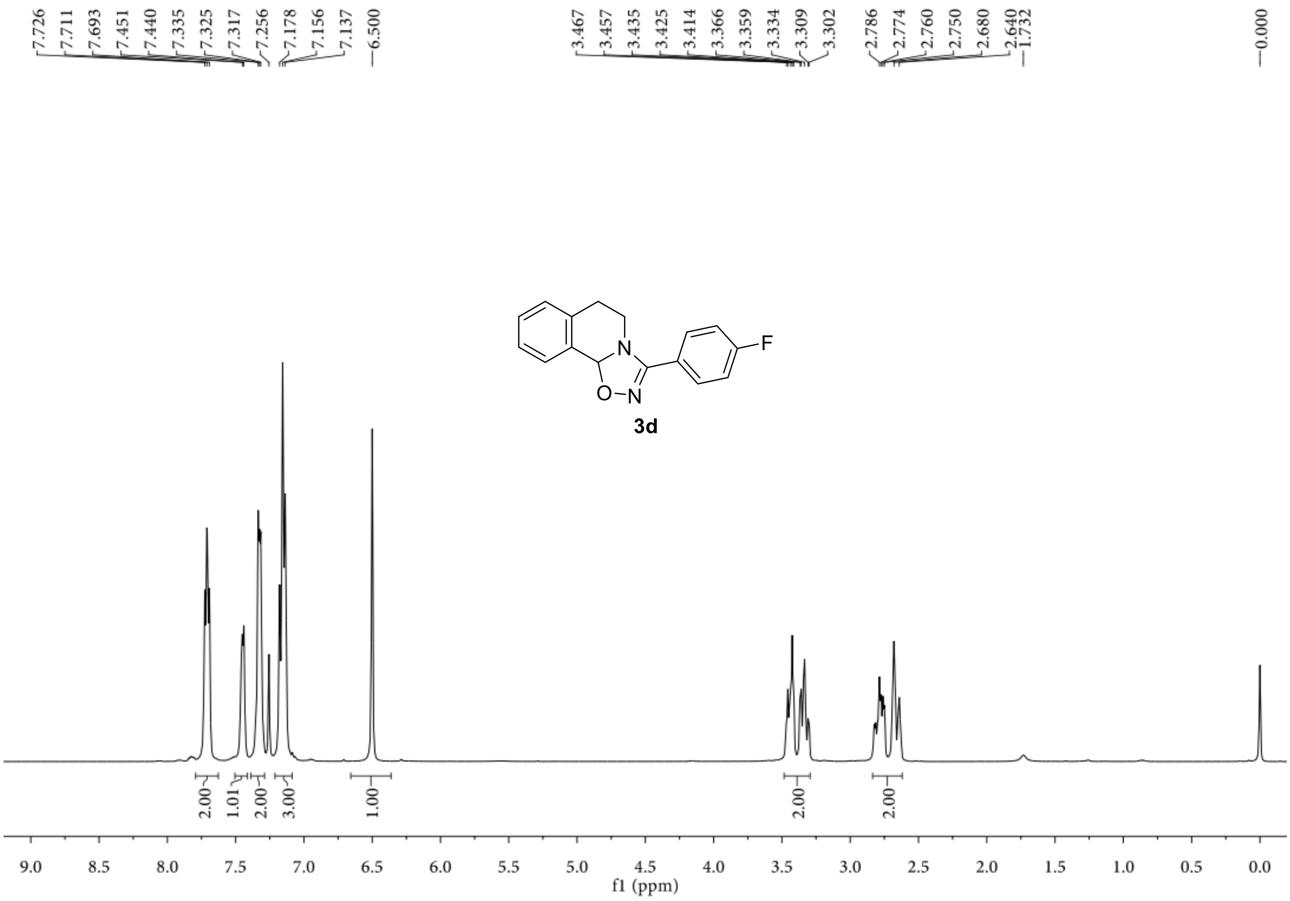
7.726
7.711
7.693
7.451
7.440
7.335
7.325
7.317
7.256
7.178
7.156
7.137
6.500
3.467
3.457
3.435
3.425
3.414
3.366
3.359
3.334
3.309
3.302
2.786
2.774
2.760
2.750
2.680
2.640
1.732
0.000
F
N
O
N
3d
2.00
1.01
2.00
3.00
1.00
2.00
2.00
f1 (ppm)

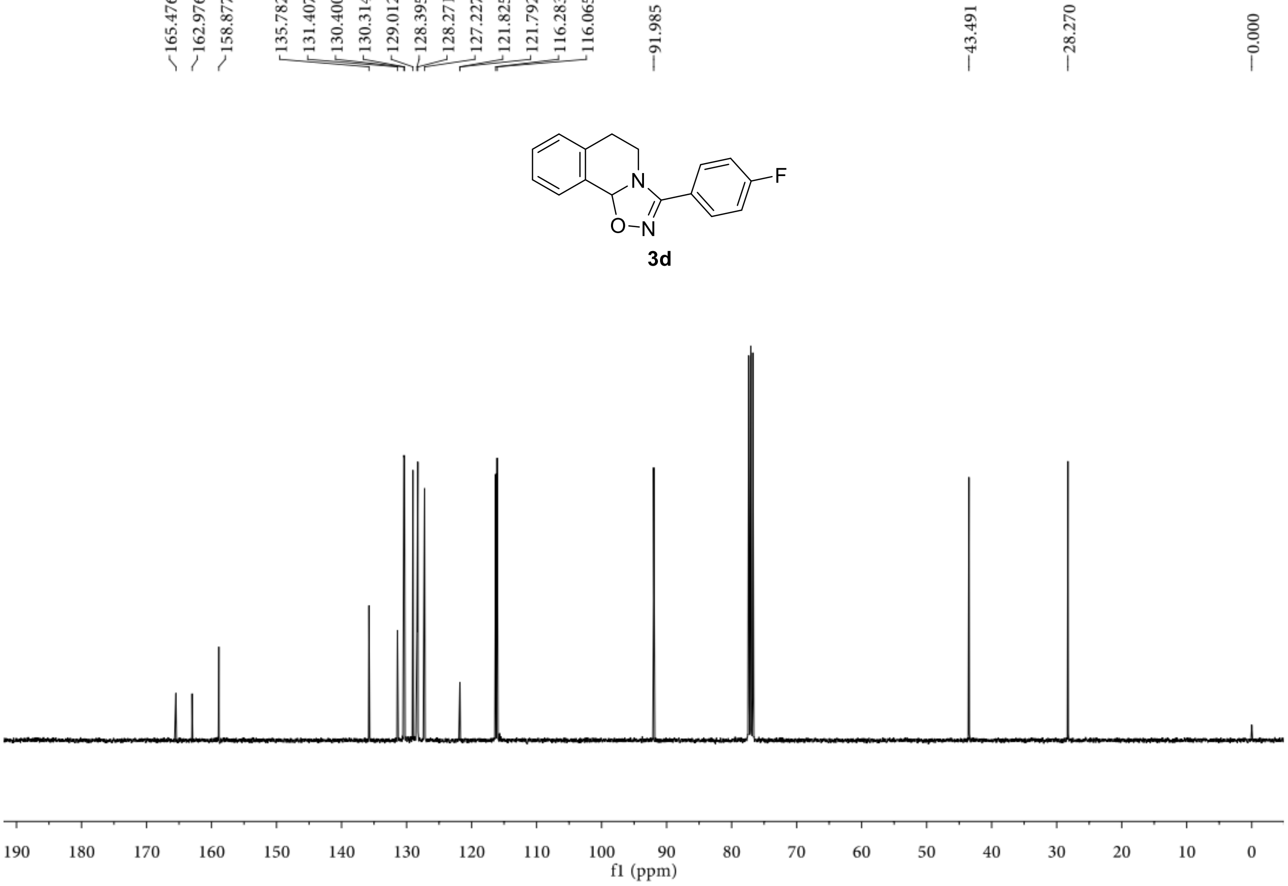
165.476
162.976
158.877
135.782
131.407
130.400
130.314
129.012
128.395
128.271
127.227
121.825
121.792
116.283
116.065
91.985
43.491
28.270
0.000
F
N
O
N
3d
f1 (ppm)

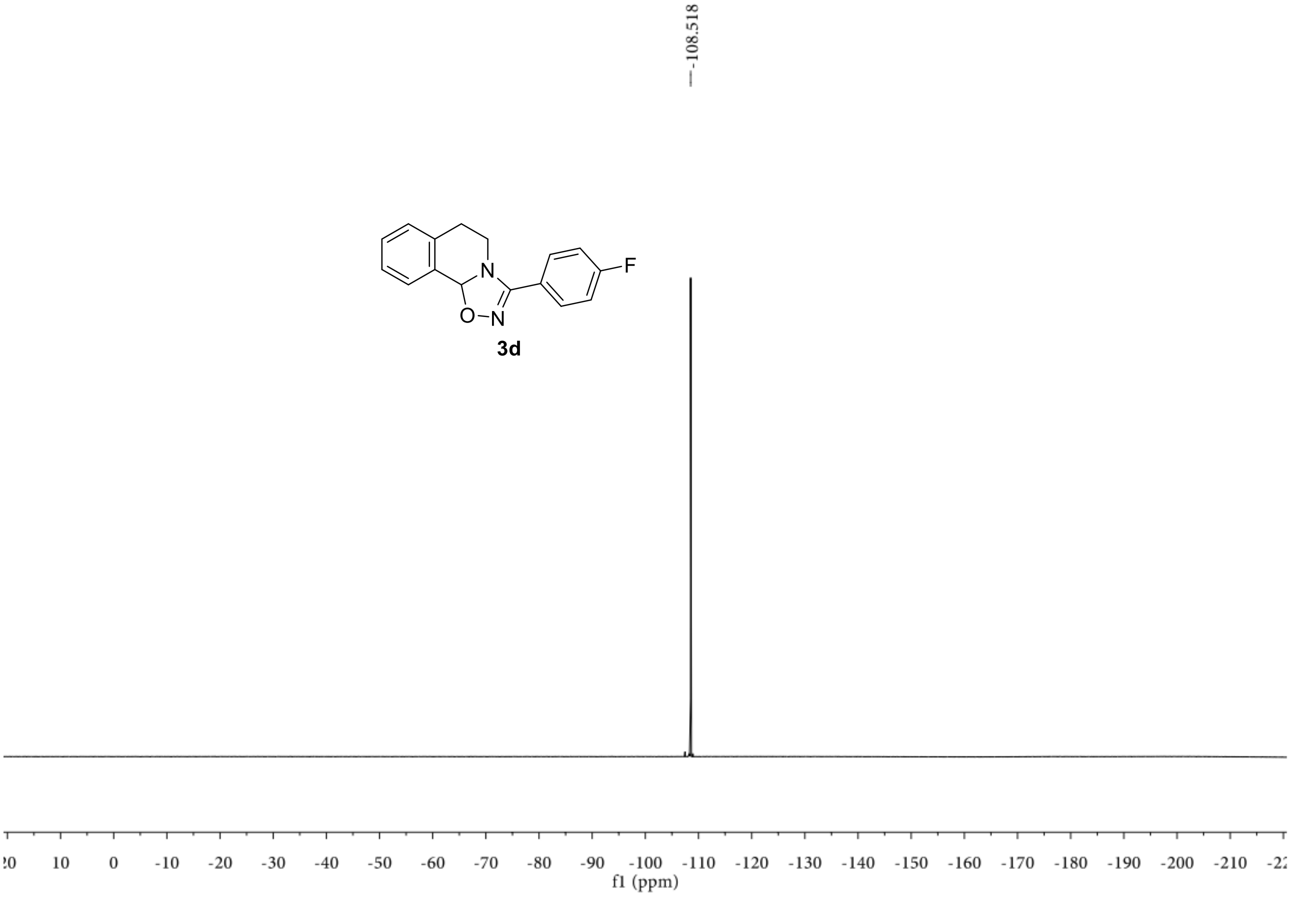

-108.518
N
F
O
N
3d
20 10 0 -10 -20 -30 -40 -50 -60 -70 -80 -90 -100 -110 -120 -130 -140 -150 -160 -170 -180 -190 -200 -210 -22
f1 (ppm)

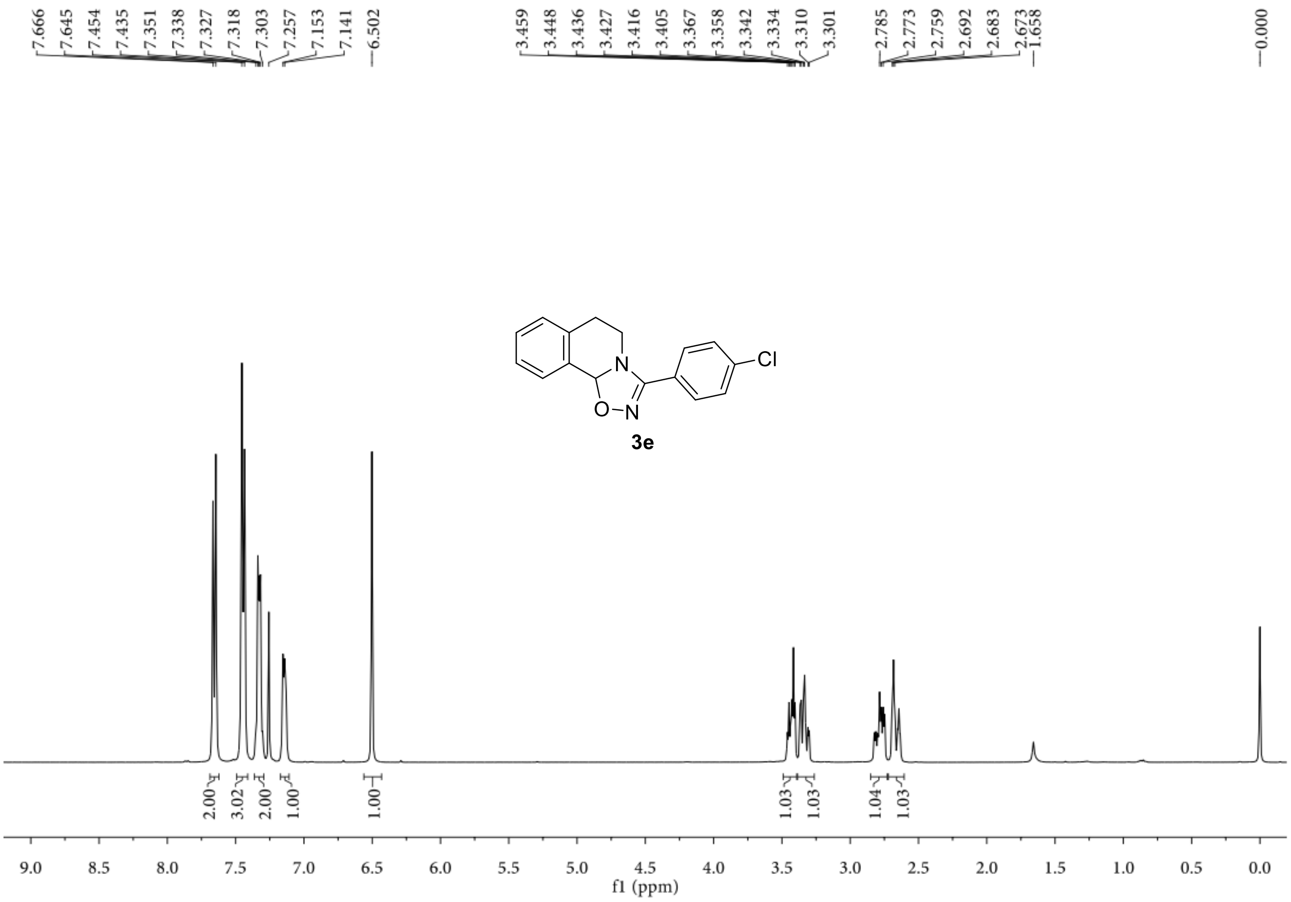

7.666
7.645
7.454
7.435
7.351
7.338
7.327
7.318
7.303
7.257
7.153
7.141
6.502
3.459
3.448
3.436
3.427
3.416
3.405
3.367
3.358
3.342
3.334
3.310
3.301
2.785
2.773
2.759
2.692
2.683
2.673
1.658
0.000
Cl
N
O
N
3e
2.00
3.02
2.00
1.00
1.00
1.03
1.03
1.04
1.03
9.0
8.5
8.0
7.5
7.0
6.5
6.0
5.5
5.0
4.5
4.0
3.5
3.0
2.5
2.0
1.5
1.0
0.5
0.0
f1 (ppm)

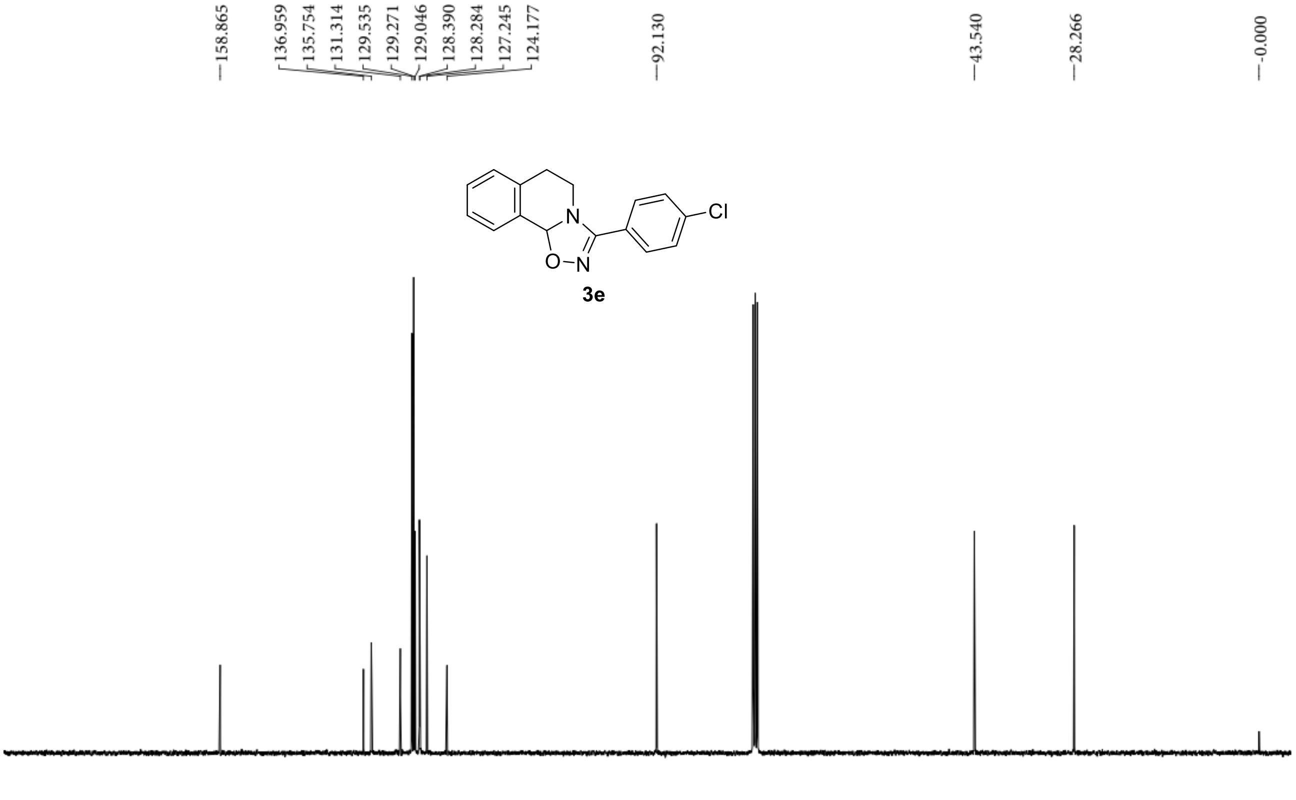

158.865
136.959
135.754
131.314
129.535
129.271
129.046
128.390
128.284
127.245
124.177
92.130
43.540
28.266
-0.000
Cl
N
O
N
3e
190
180
170
160
150
140
130
120
110
100
90
80
70
60
50
40
30
20
10
0
f1 (ppm)

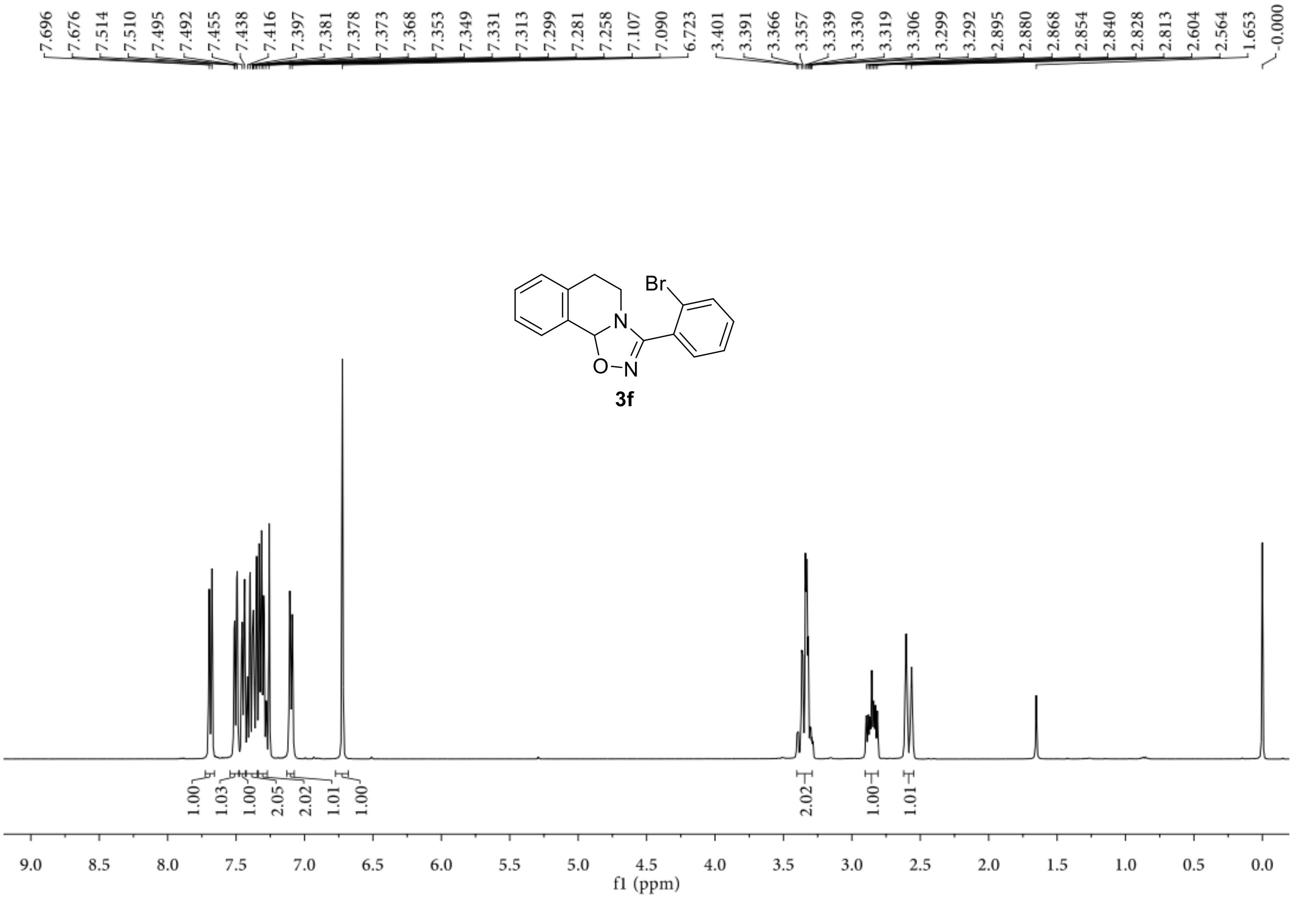
7.696 7.676 7.514 7.510 7.495 7.492 7.455 7.438 7.416 7.397 7.381 7.378 7.373 7.368 7.353 7.349 7.331 7.313 7.299 7.281 7.258 7.107 7.090 6.723
3.401 3.391 3.366 3.357 3.339 3.330 3.319 3.306 3.299 3.292 2.895 2.880 2.868 2.854 2.840 2.828 2.813 2.604 2.564 1.653 -0.000
Br
N
O
N
3f
1.00 1.03 1.00 2.05 2.02 1.01 1.00
2.02 1.00 1.01
9.0 8.5 8.0 7.5 7.0 6.5 6.0 5.5 5.0 4.5 4.0 3.5 3.0 2.5 2.0 1.5 1.0 0.5 0.0
f1 (ppm)

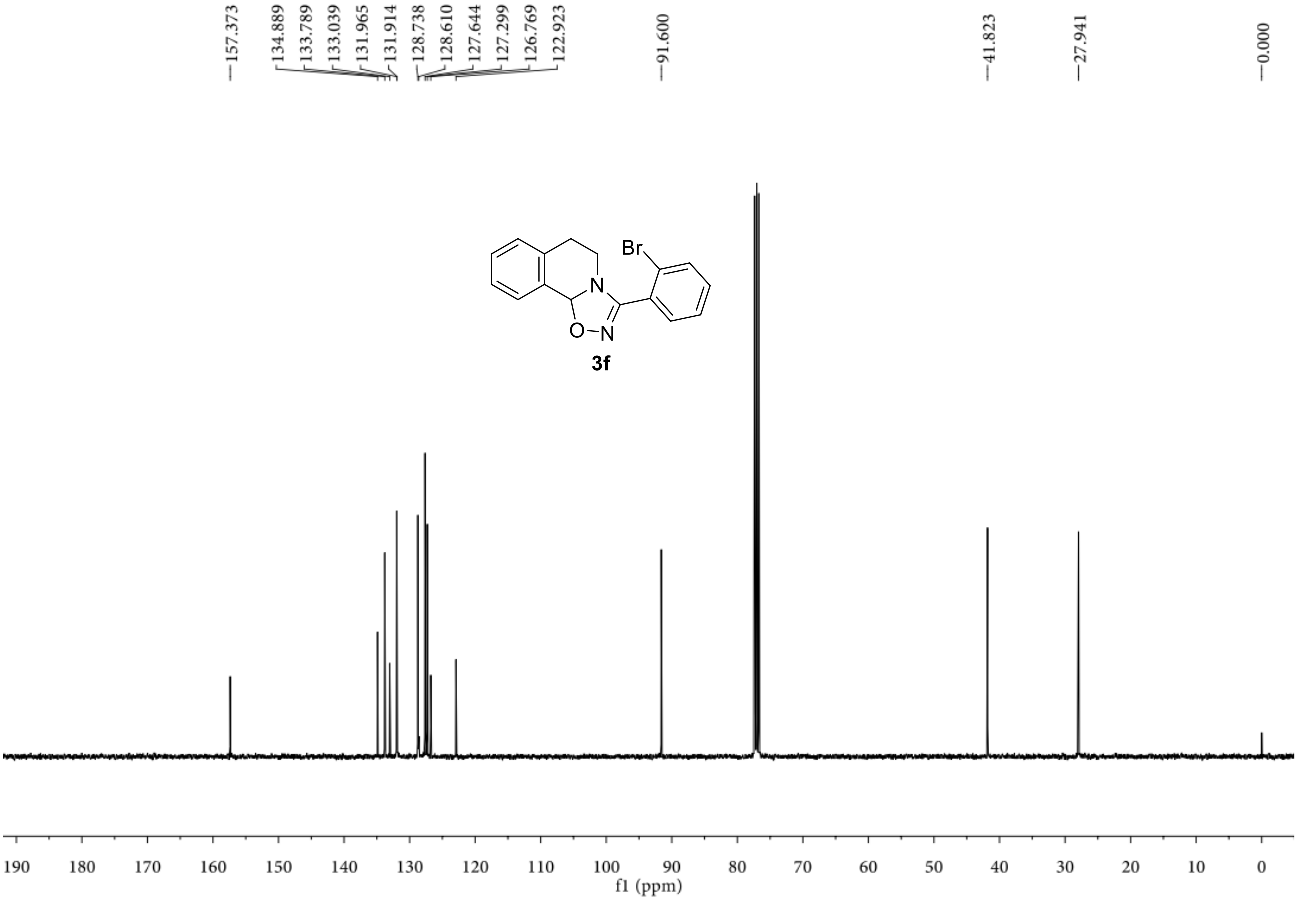
157.373
134.889 133.789 133.039 131.965 131.914 128.738 128.610 127.644 127.299 126.769 122.923
91.600
41.823
27.941
0.000
Br
N
O
N
3f
190 180 170 160 150 140 130 120 110 100 90 80 70 60 50 40 30 20 10 0
f1 (ppm)

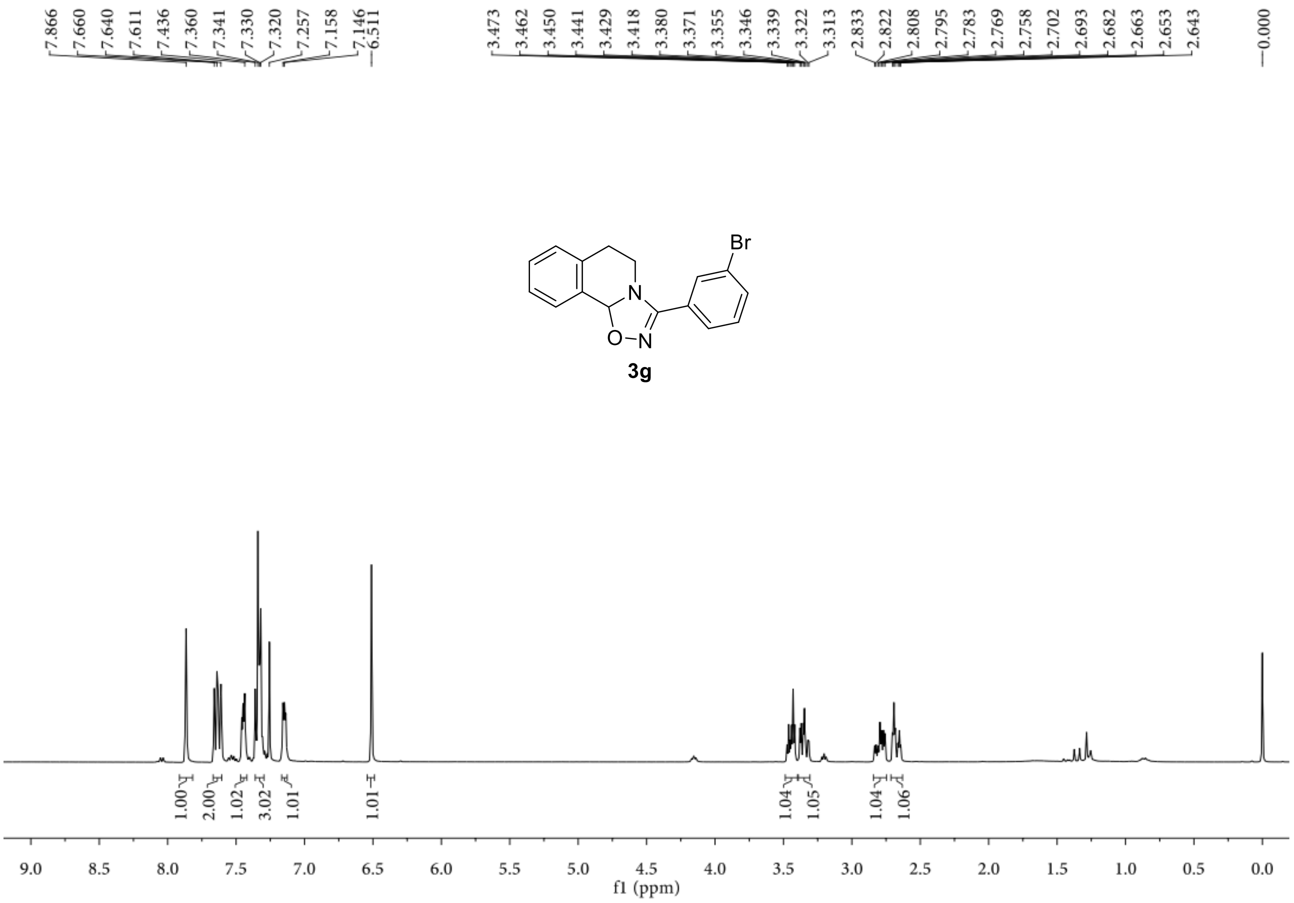
7.866
7.660
7.640
7.611
7.436
7.360
7.341
7.330
7.320
7.257
7.158
7.146
6.511
3.473
3.462
3.450
3.441
3.429
3.418
3.380
3.371
3.355
3.346
3.339
3.322
3.313
2.833
2.822
2.808
2.795
2.783
2.769
2.758
2.702
2.693
2.682
2.663
2.653
2.643
0.000
Br
N
O
N
3g
1.00
2.00
1.02
3.02
1.01
1.01
1.04
1.05
1.04
1.06
9.0 8.5 8.0 7.5 7.0 6.5 6.0 5.5 5.0 4.5 4.0 3.5 3.0 2.5 2.0 1.5 1.0 0.5 0.0
f1 (ppm)

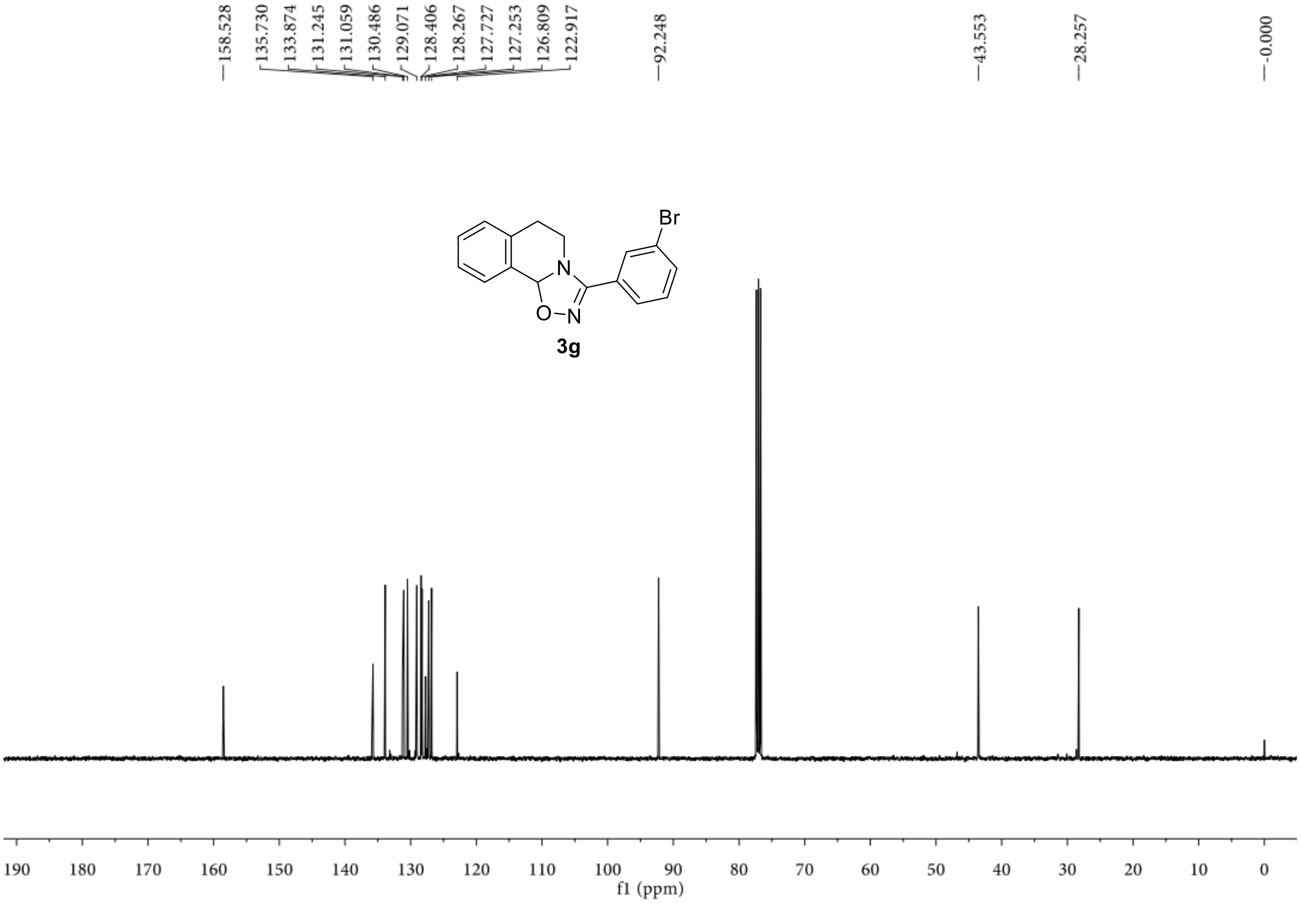
158.528
135.730
133.874
131.245
131.059
130.486
129.071
128.406
128.267
127.727
127.253
126.809
122.917
92.248
43.553
28.257
-0.000
Br
N
O
N
3g
190 180 170 160 150 140 130 120 110 100 90 80 70 60 50 40 30 20 10 0
f1 (ppm)

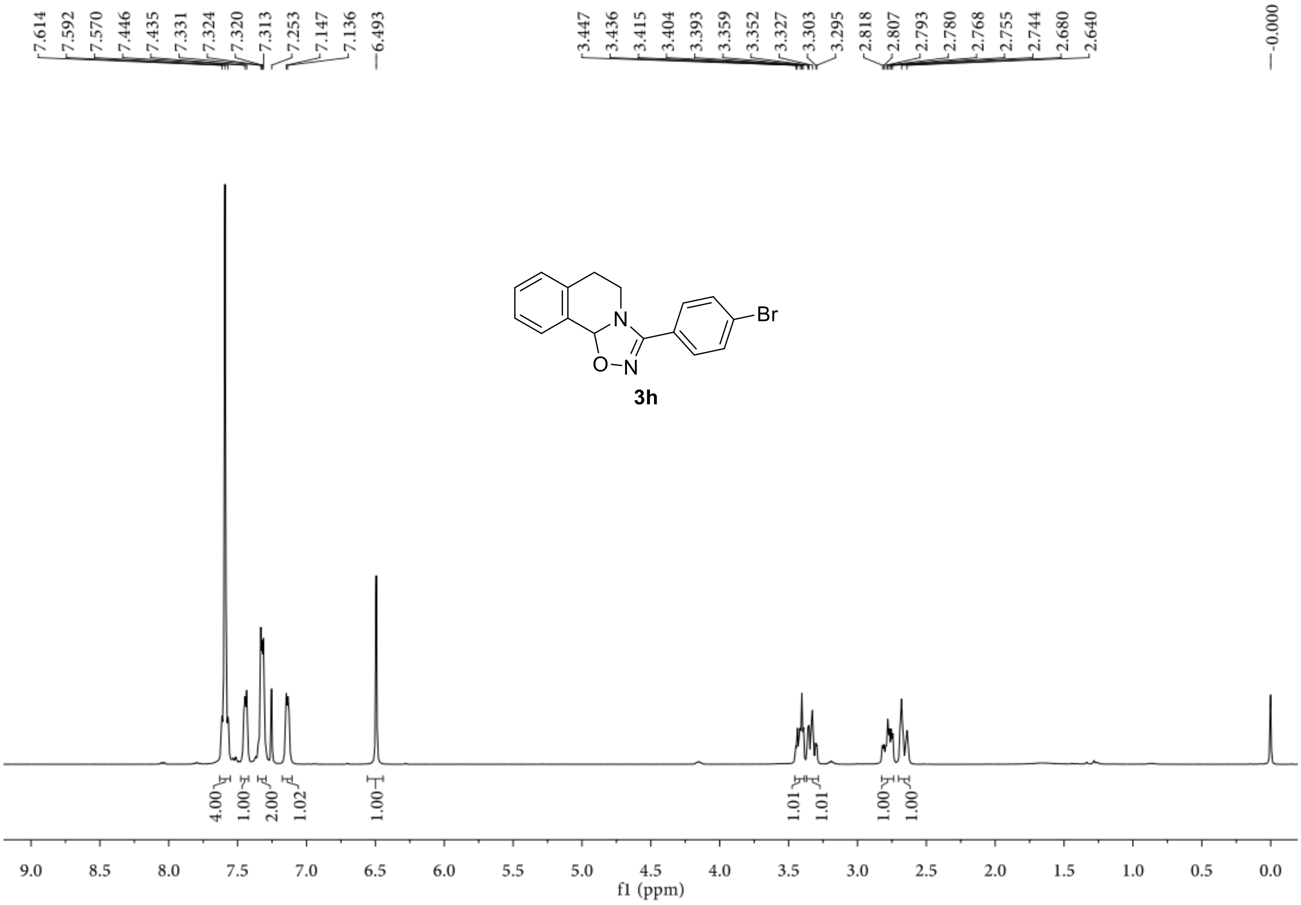
7.614
7.592
7.570
7.446
7.435
7.331
7.324
7.320
7.313
7.253
7.147
7.136
6.493
3.447
3.436
3.415
3.404
3.393
3.359
3.352
3.327
3.303
3.295
2.818
2.807
2.793
2.780
2.768
2.755
2.744
2.680
2.640
-0.000
Br
N
O
N
3h
4.00
1.00
2.00
1.02
1.00
1.01
1.01
1.00
1.00
9.0 8.5 8.0 7.5 7.0 6.5 6.0 5.5 5.0 4.5 4.0 3.5 3.0 2.5 2.0 1.5 1.0 0.5 0.0
f1 (ppm)

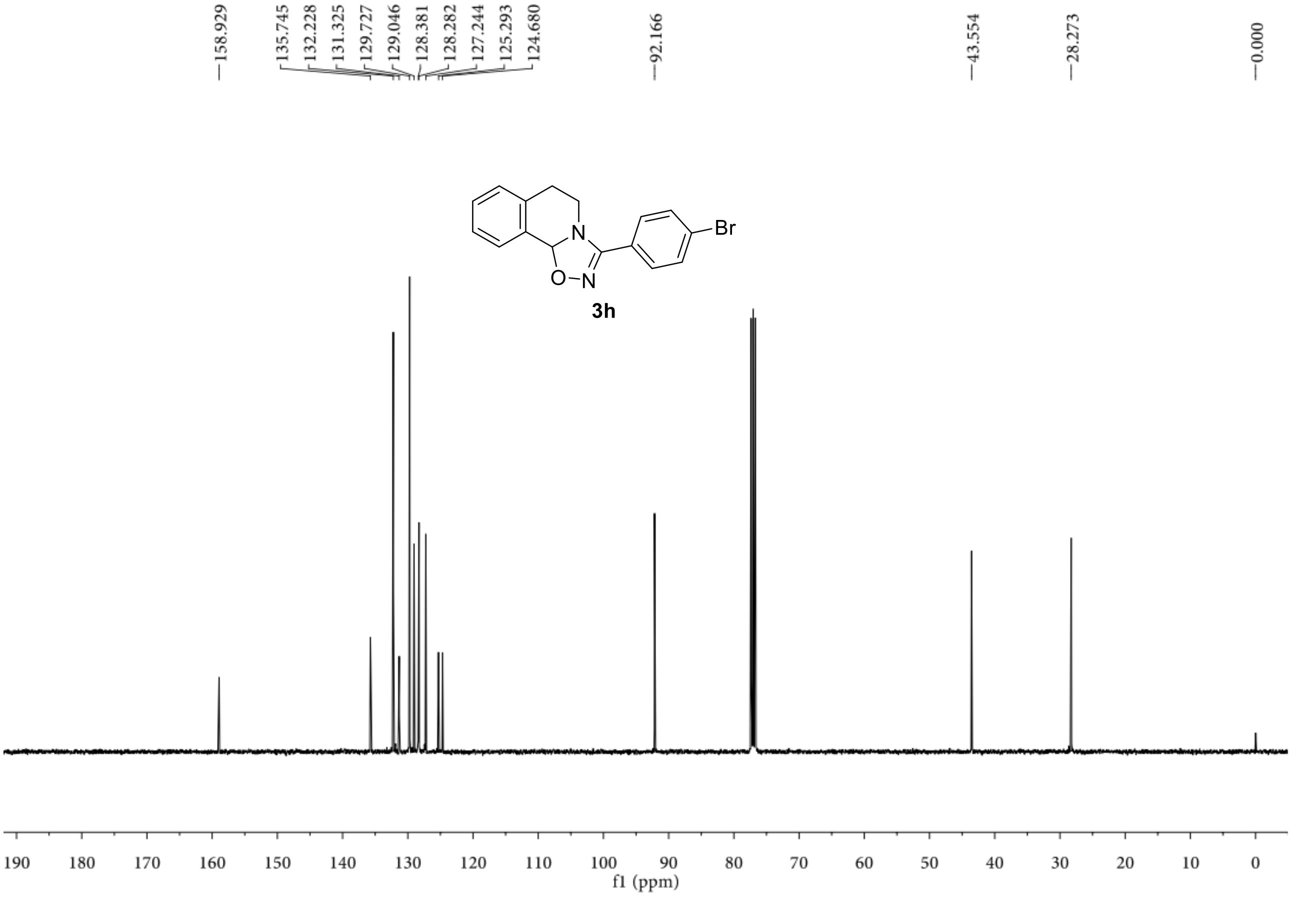
158.929
135.745
132.228
131.325
129.727
129.046
128.381
128.282
127.244
125.293
124.680
92.166
43.554
28.273
-0.000
Br
N
O
N
3h
190 180 170 160 150 140 130 120 110 100 90 80 70 60 50 40 30 20 10 0
f1 (ppm)

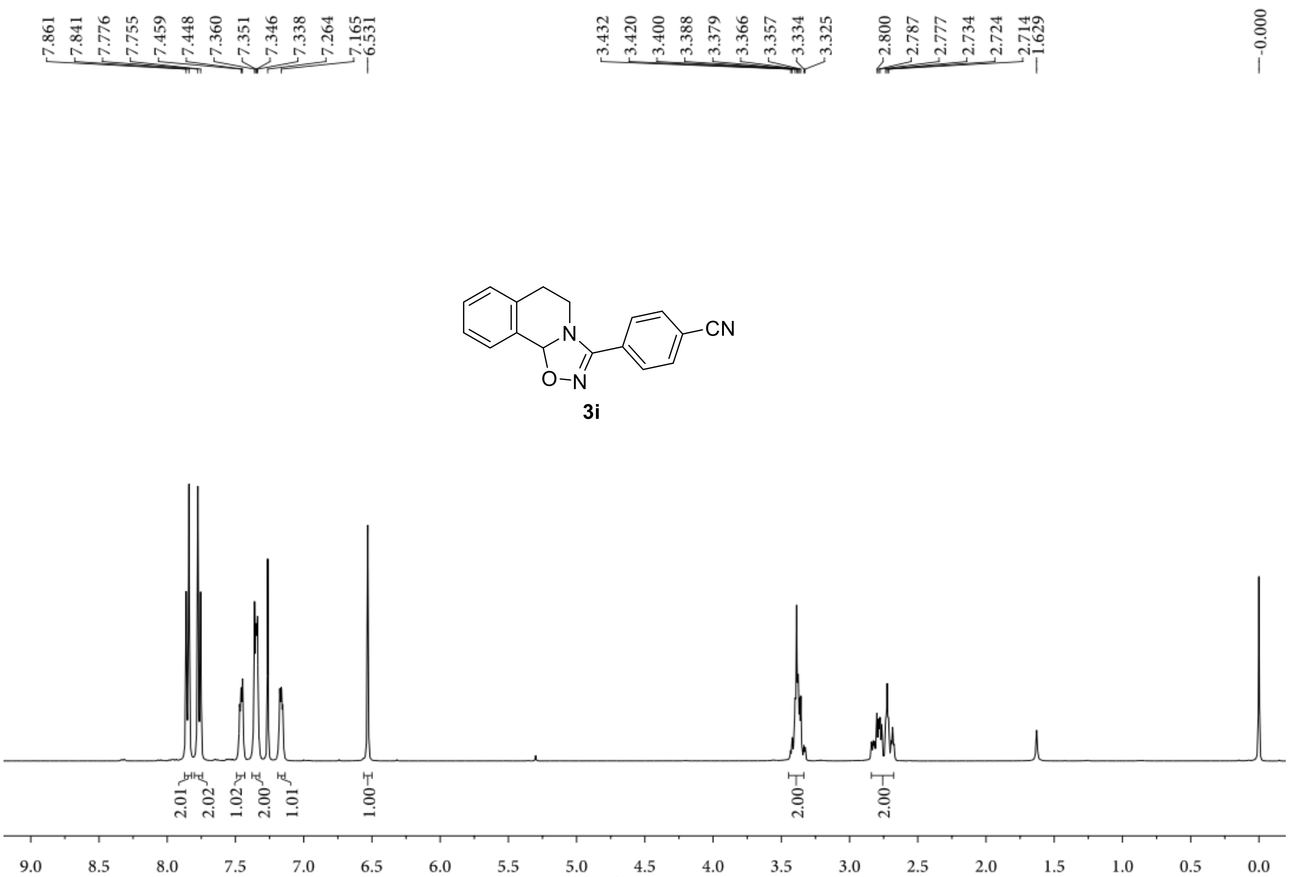
7.861
7.841
7.776
7.755
7.459
7.448
7.360
7.351
7.346
7.338
7.264
7.165
6.531
3.432
3.420
3.400
3.388
3.379
3.366
3.357
3.334
3.325
2.800
2.787
2.777
2.734
2.724
2.714
1.629
-0.000
CN
N
O
N
3i
2.01
2.02
1.02
2.00
1.01
1.00
2.00
2.00
9.0 8.5 8.0 7.5 7.0 6.5 6.0 5.5 5.0 4.5 4.0 3.5 3.0 2.5 2.0 1.5 1.0 0.5 0.0
f1 (ppm)

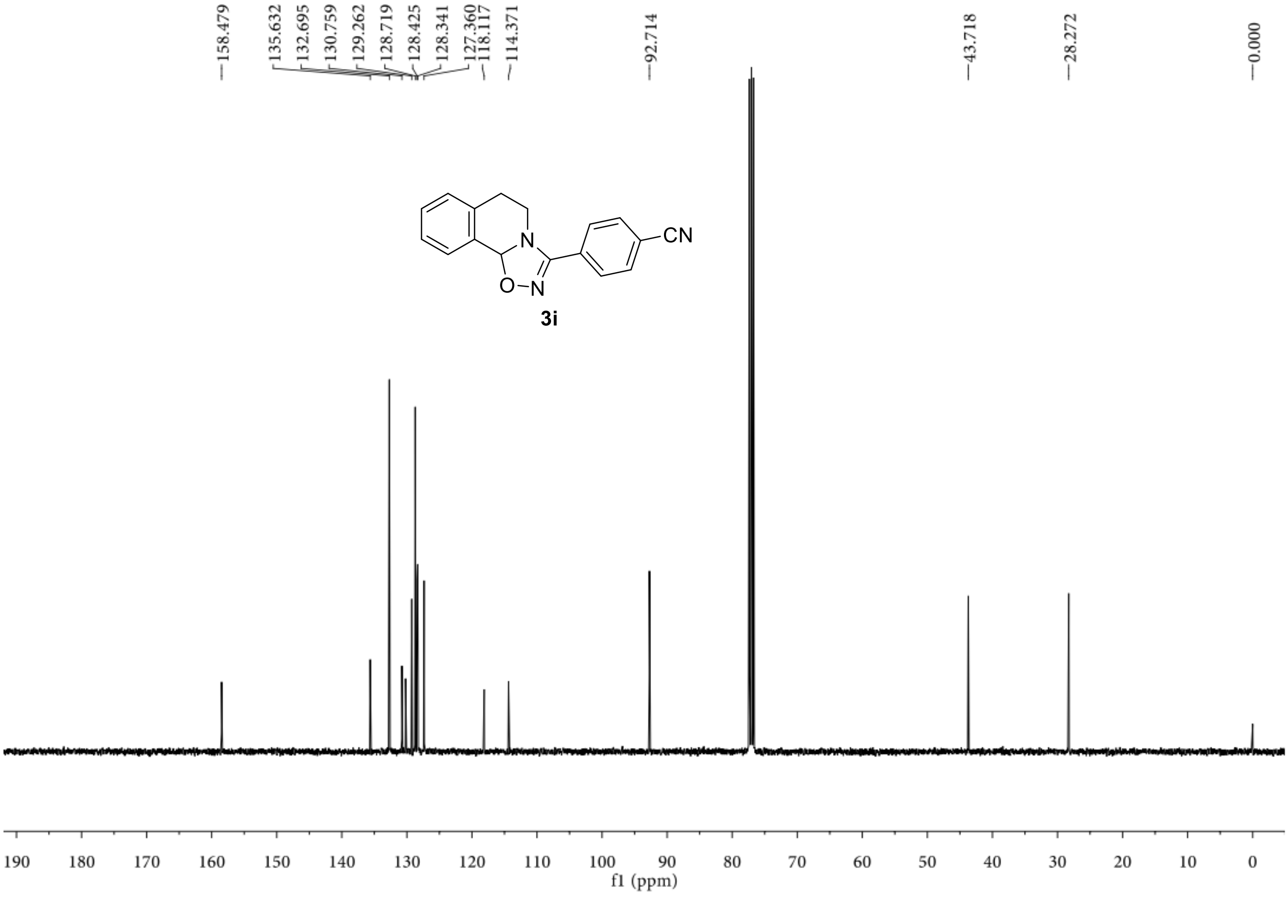
158.479
135.632
132.695
130.759
129.262
128.719
128.425
128.341
127.360
118.117
114.371
92.714
43.718
28.272
0.000
CN
N
O
N
3i
190 180 170 160 150 140 130 120 110 100 90 80 70 60 50 40 30 20 10 0
f1 (ppm)

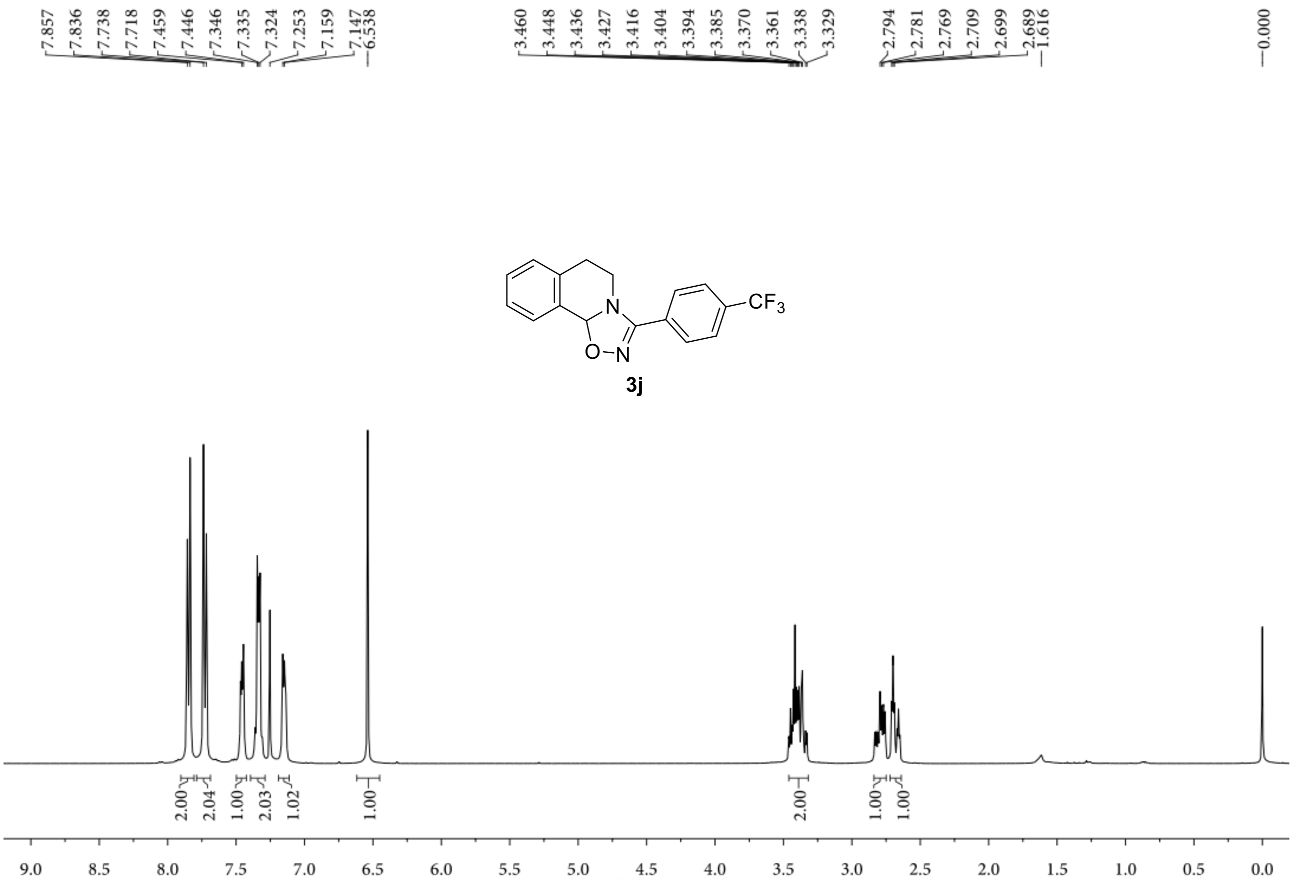
7.857
7.836
7.738
7.718
7.459
7.446
7.346
7.335
7.324
7.253
7.159
7.147
6.538
3.460
3.448
3.436
3.427
3.416
3.404
3.394
3.385
3.370
3.361
3.338
3.329
2.794
2.781
2.769
2.709
2.699
2.689
1.616
0.000
CF_3
3j
2.00
2.04
1.00
2.03
1.02
1.00
2.00
1.00
1.00
f1 (ppm)

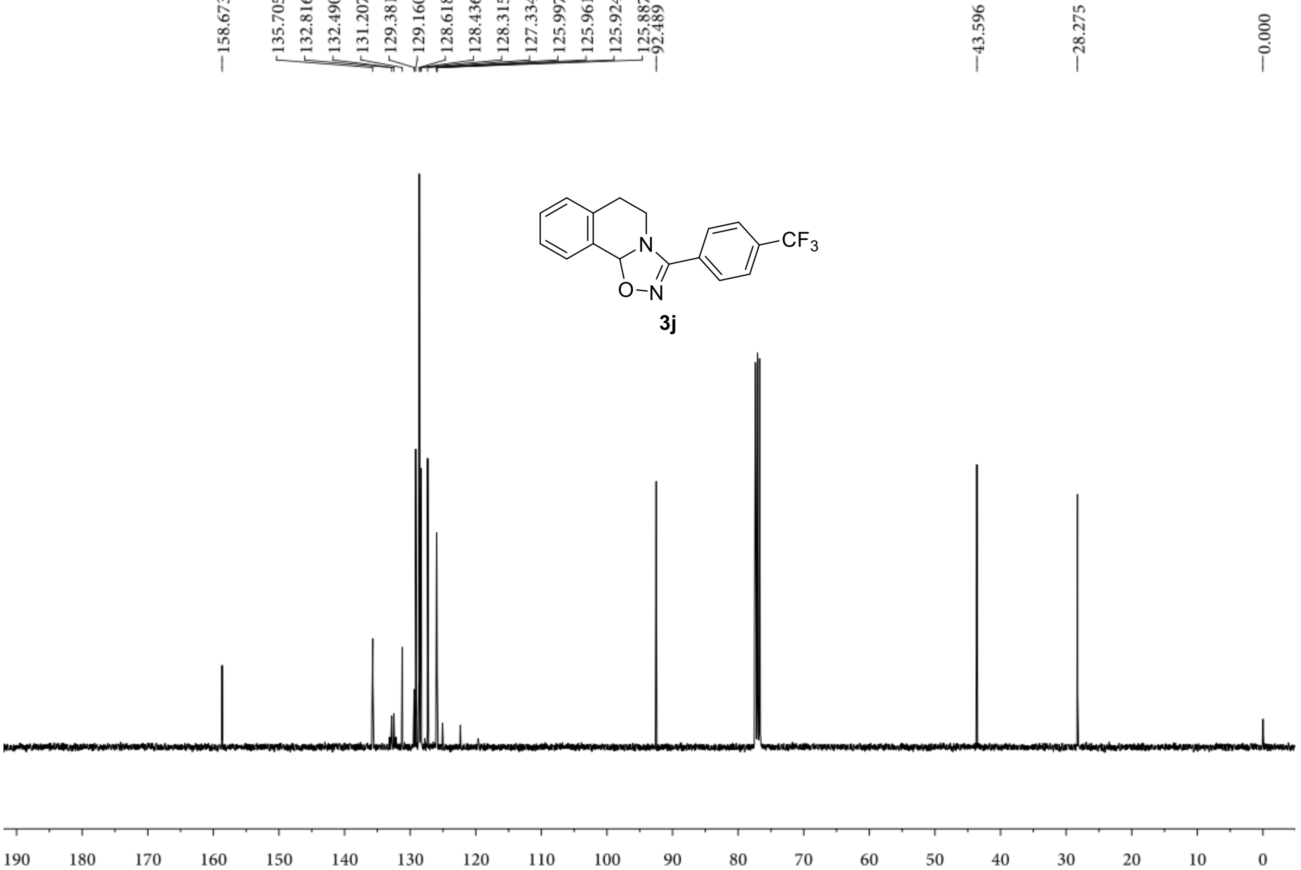
158.673
135.705
132.816
132.490
131.207
129.381
129.160
128.618
128.436
128.315
127.334
125.997
125.961
125.924
125.887
92.489
43.596
28.275
0.000
CF_3
3j
f1 (ppm)

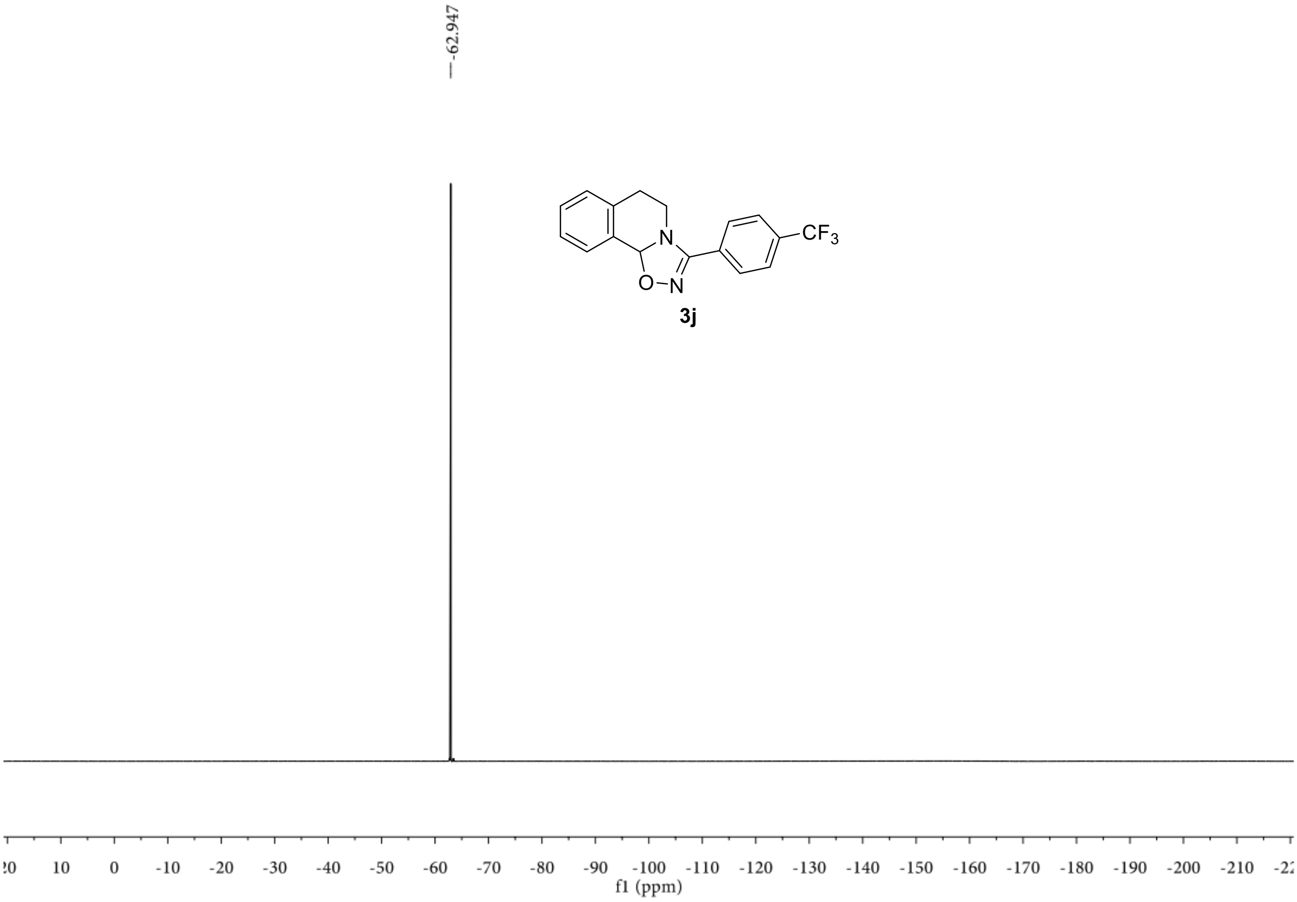
-62.947
CF3
O–N
N
3j
20 10 0 -10 -20 -30 -40 -50 -60 -70 -80 -90 -100 -110 -120 -130 -140 -150 -160 -170 -180 -190 -200 -210
f1 (ppm)

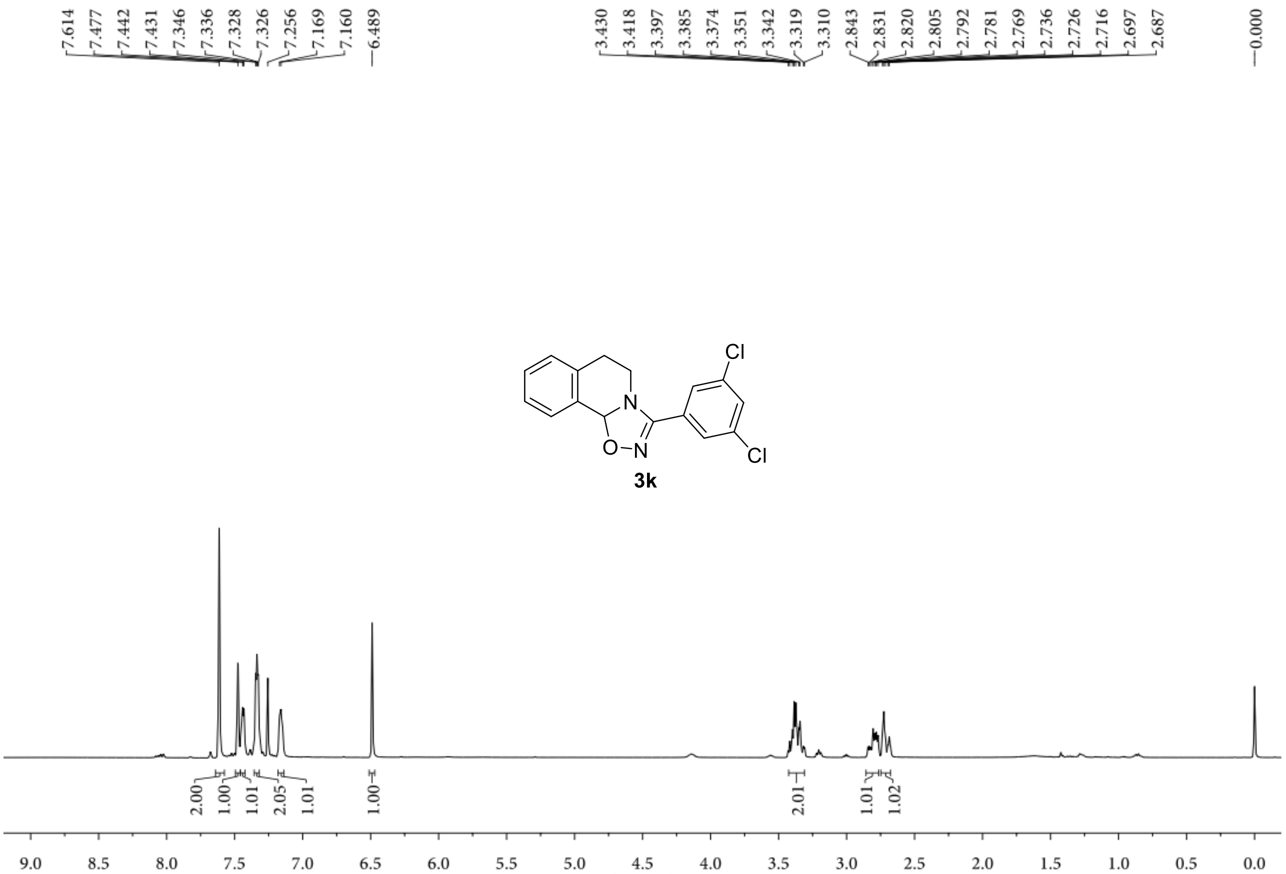
7.614
7.477
7.442
7.431
7.346
7.336
7.328
7.326
7.256
7.169
7.160
6.489
3.430
3.418
3.397
3.385
3.374
3.351
3.342
3.319
3.310
2.843
2.831
2.820
2.805
2.792
2.781
2.769
2.736
2.726
2.716
2.697
2.687
0.000
Cl
N
O
N
Cl
3k
2.00
1.00
1.01
2.05
1.01
1.00
2.01
1.01
1.02
f1 (ppm)

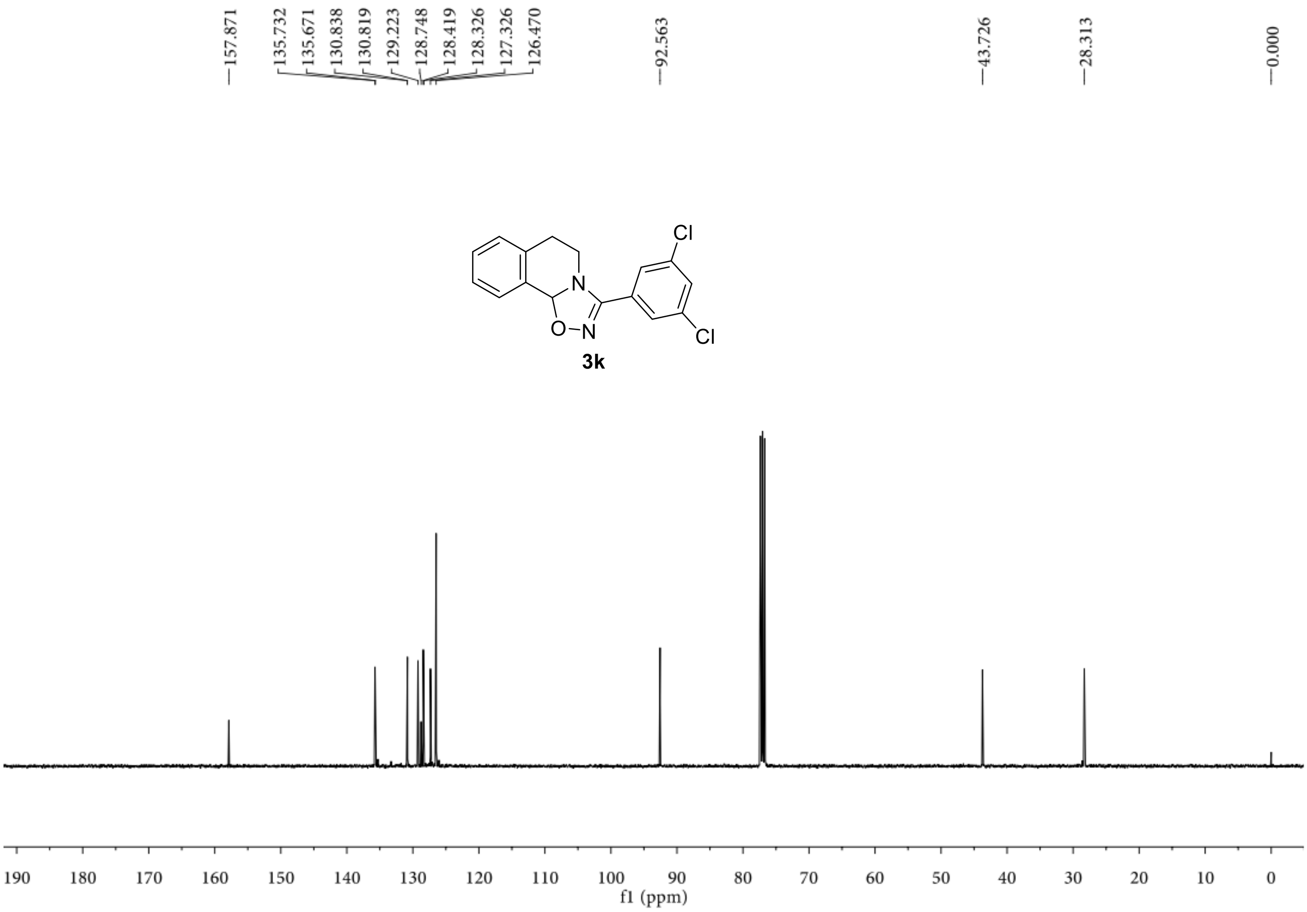
157.871
135.732
135.671
130.838
130.819
129.223
128.748
128.419
128.326
127.326
126.470
92.563
43.726
28.313
0.000
Cl
N
O
N
Cl
3k
f1 (ppm)

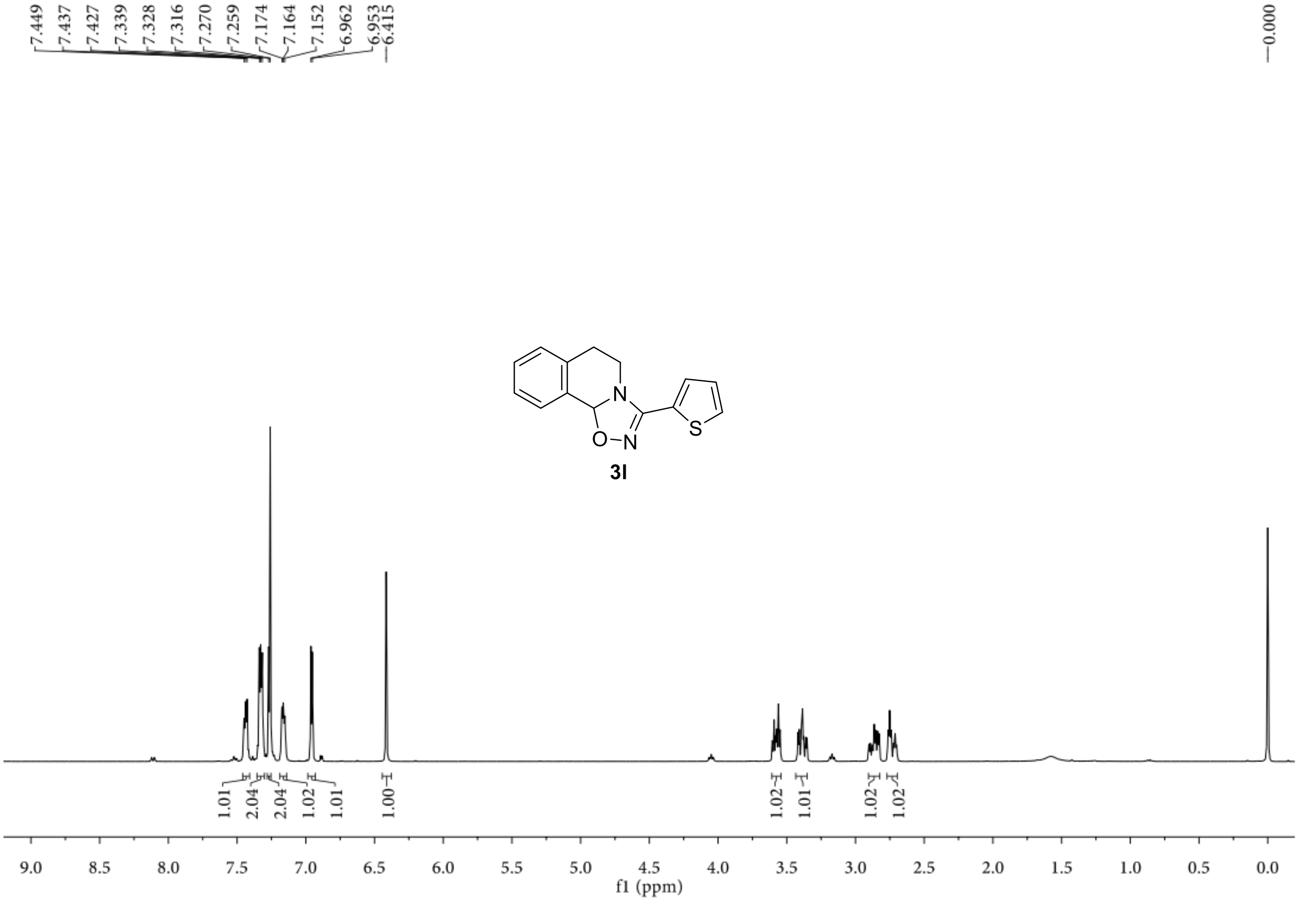
7.449
7.437
7.427
7.339
7.328
7.316
7.270
7.259
7.174
7.164
7.152
6.962
6.953
6.415
0.000
1.01
2.04
2.04
1.02
1.01
1.00
1.02
1.01
1.02
1.02
N
O
N
S
3l
f1 (ppm)

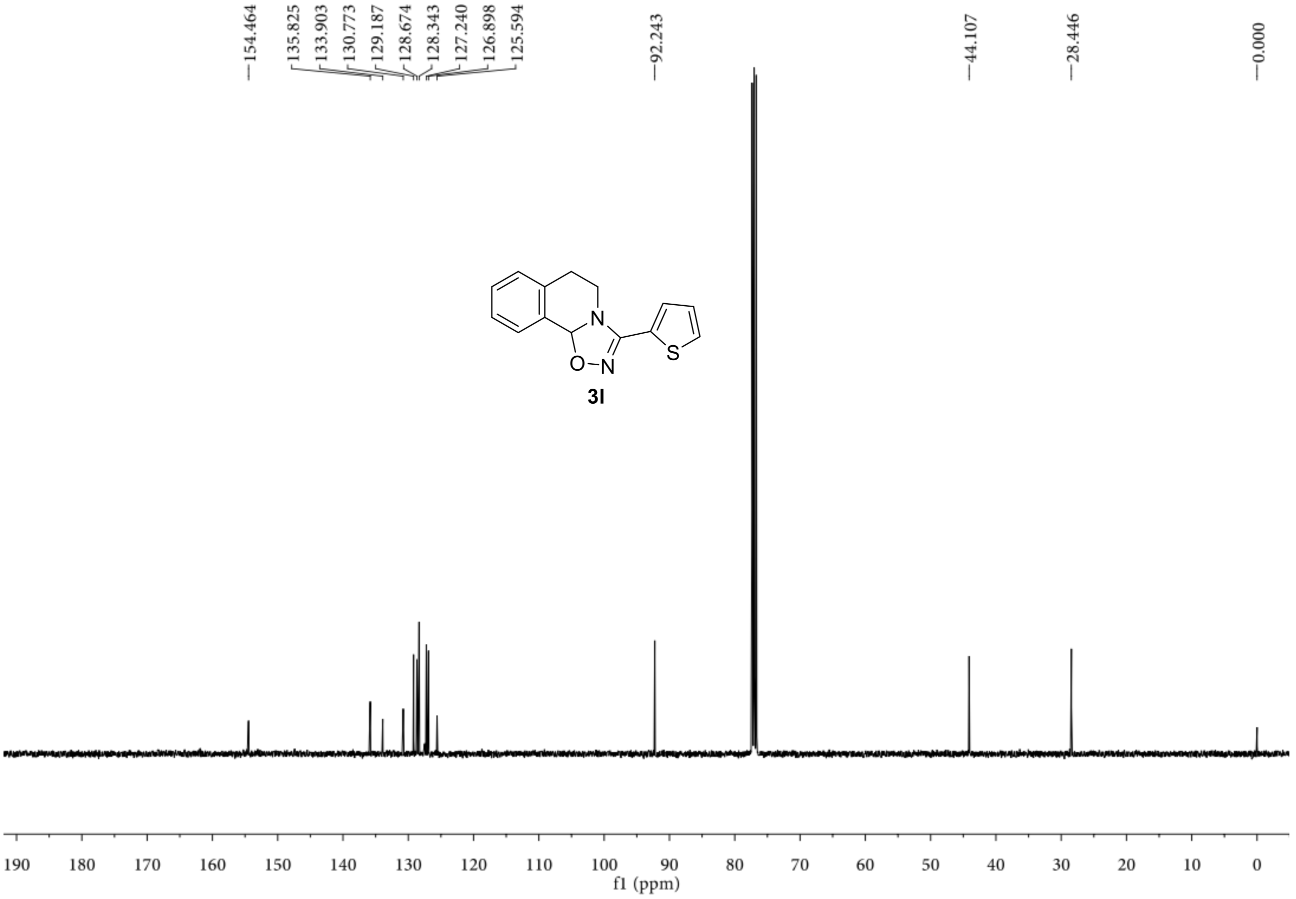
154.464
135.825
133.903
130.773
129.187
128.674
128.343
127.240
126.898
125.594
92.243
44.107
28.446
0.000
N
O
N
S
3l
f1 (ppm)

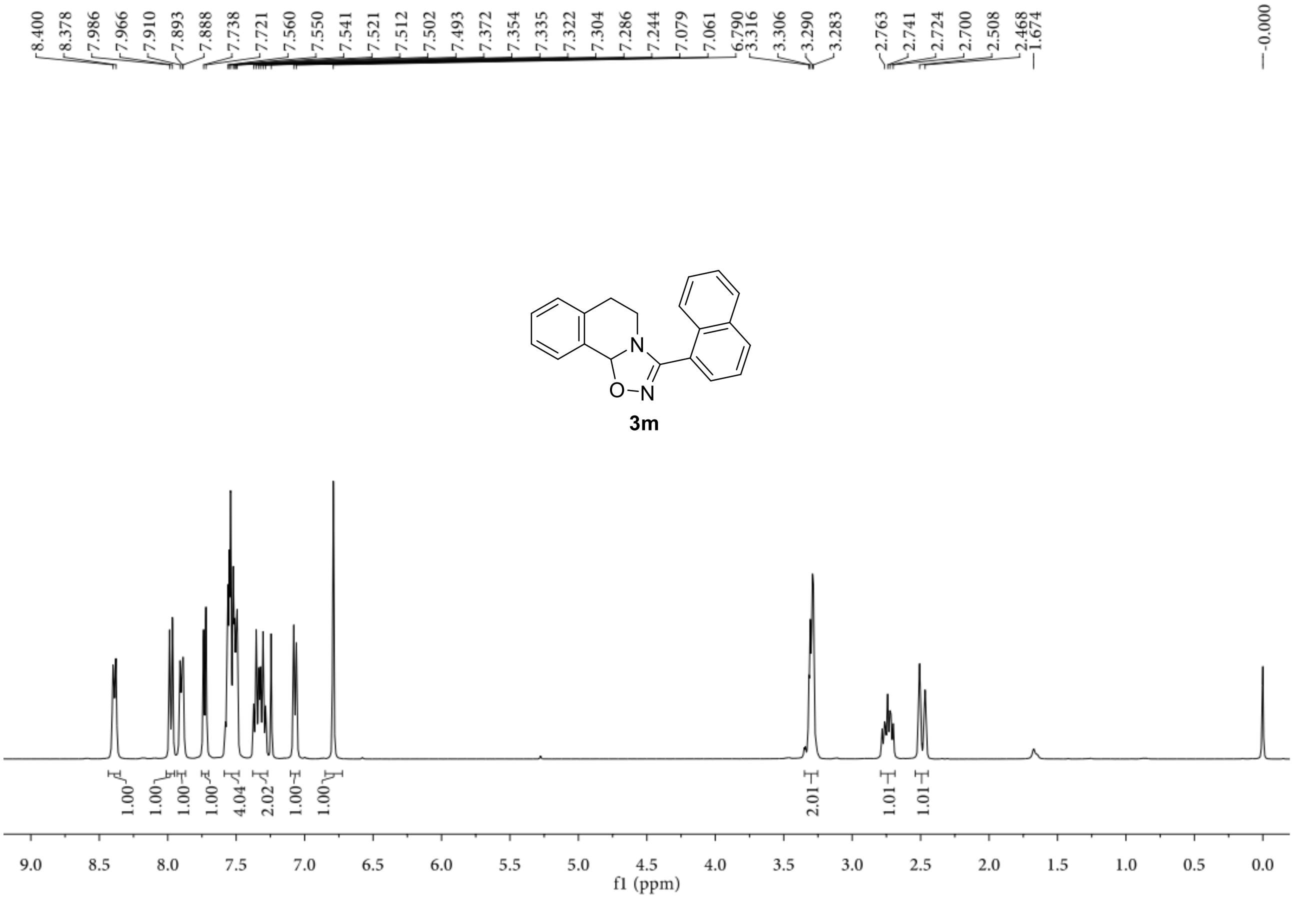
8.400
8.378
7.986
7.966
7.910
7.893
7.888
7.738
7.721
7.560
7.550
7.541
7.521
7.512
7.502
7.493
7.372
7.354
7.335
7.322
7.304
7.286
7.244
7.079
7.061
6.790
3.316
3.306
3.290
3.283
2.763
2.741
2.724
2.700
2.508
2.468
1.674
-0.000
O
N
N
3m
1.00
1.00
1.00
1.00
4.04
2.02
1.00
1.00
2.01
1.01
1.01
9.0
8.5
8.0
7.5
7.0
6.5
6.0
5.5
5.0
4.5
4.0
3.5
3.0
2.5
2.0
1.5
1.0
0.5
0.0
f1 (ppm)

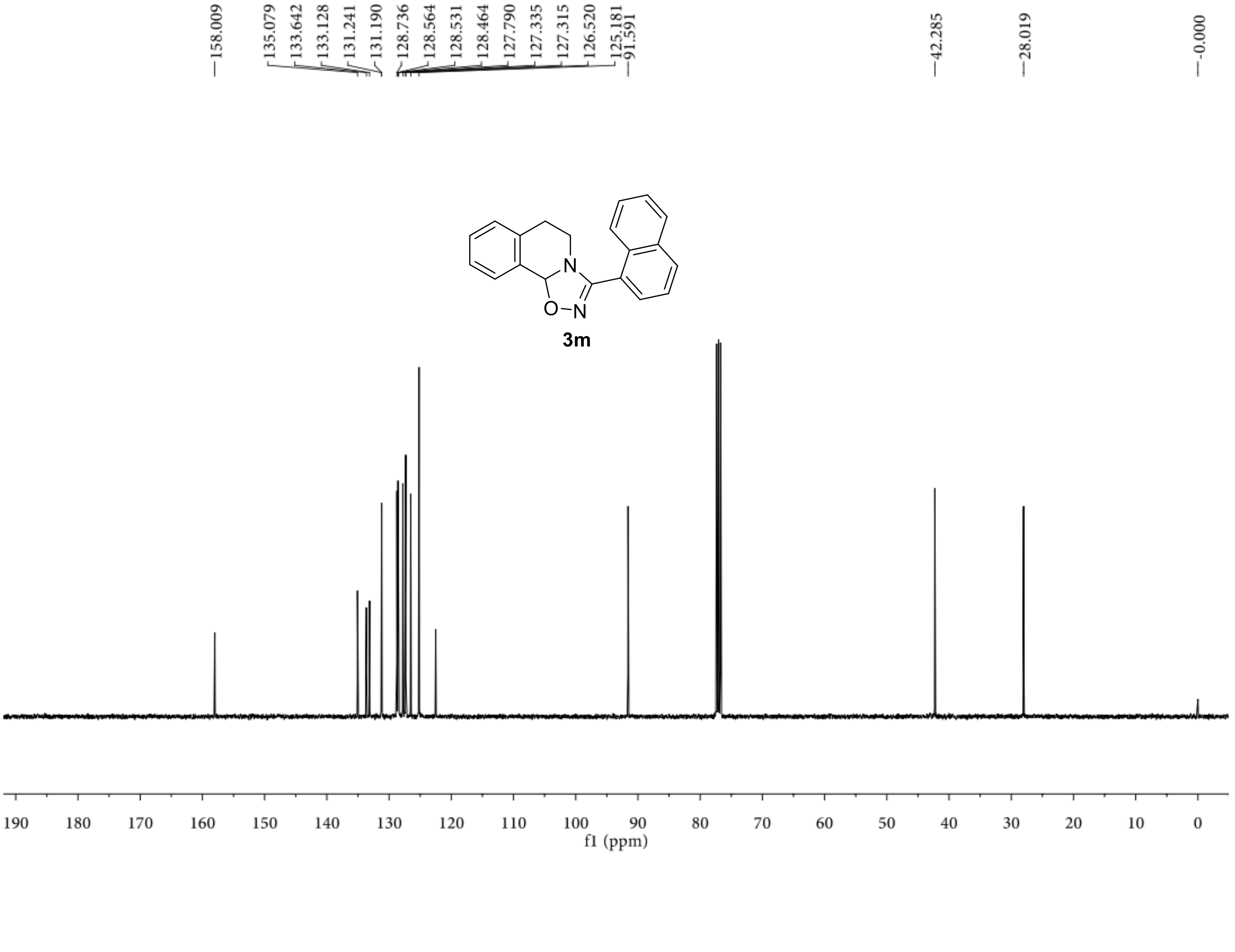
158.009
135.079
133.642
133.128
131.241
131.190
128.736
128.564
128.531
128.464
127.790
127.335
127.315
126.520
125.181
91.591
42.285
28.019
-0.000
O
N
N
3m
190
180
170
160
150
140
130
120
110
100
90
80
70
60
50
40
30
20
10
0
f1 (ppm)

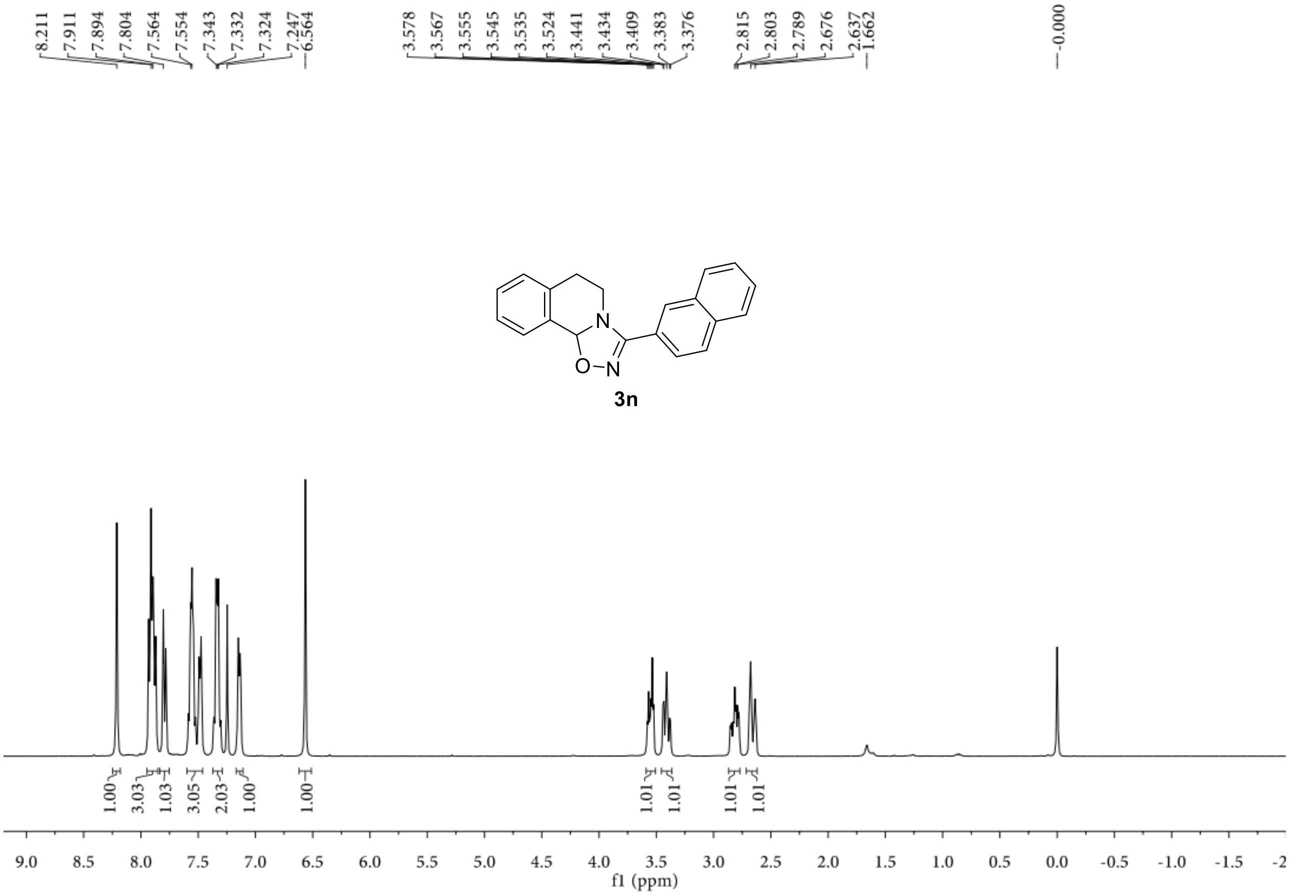
8.211
7.911
7.894
7.804
7.564
7.554
7.343
7.332
7.324
7.247
6.564
3.578
3.567
3.555
3.545
3.535
3.524
3.441
3.434
3.409
3.383
3.376
2.815
2.803
2.789
2.676
2.637
1.662
-0.000
N
O
N
3n
1.00
3.03
1.03
3.05
2.03
1.00
1.00
1.01
1.01
1.01
1.01
f1 (ppm)

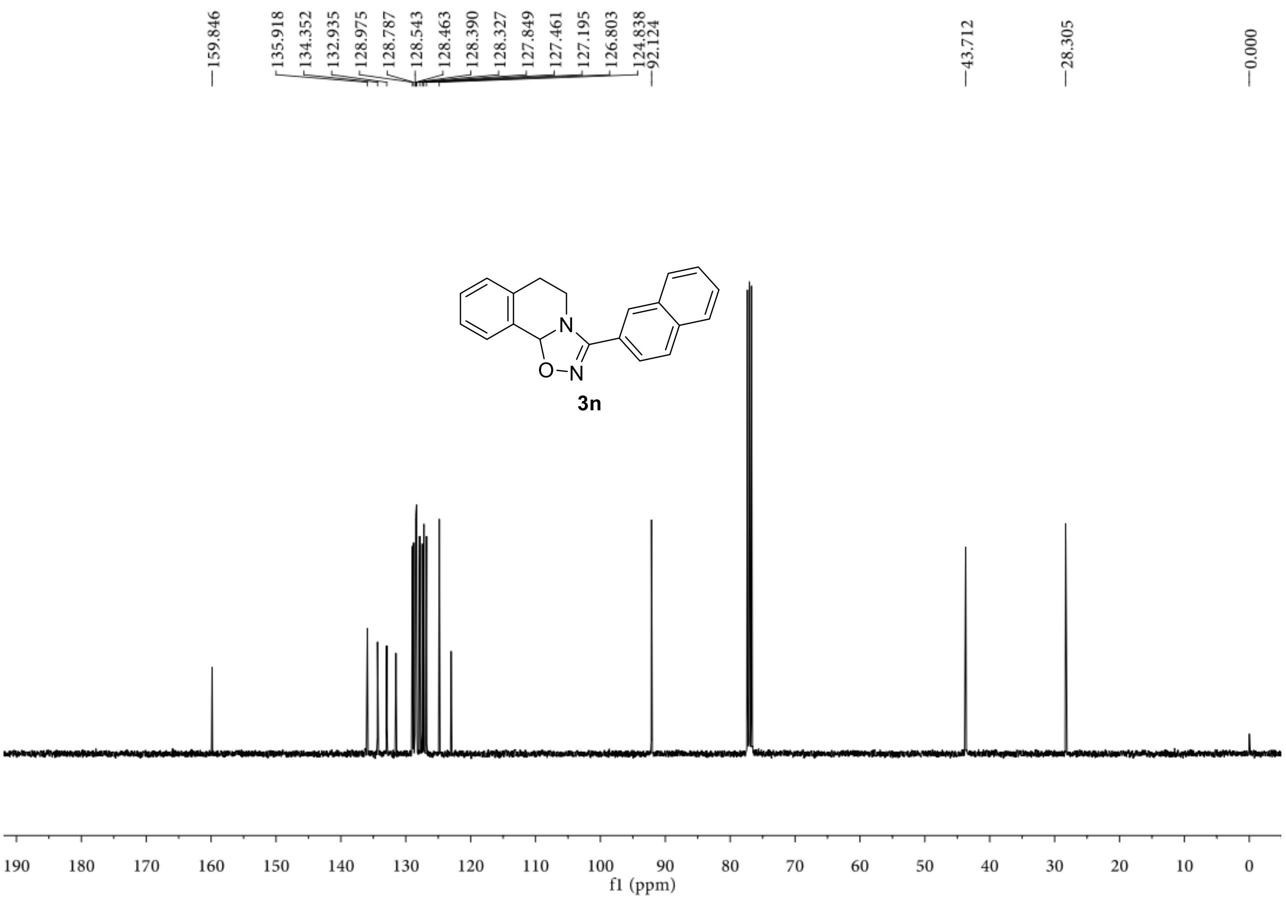
159.846
135.918
134.352
132.935
128.975
128.787
128.543
128.463
128.390
128.327
127.849
127.461
127.195
126.803
124.838
92.124
43.712
28.305
0.000
N
O
N
3n
f1 (ppm)

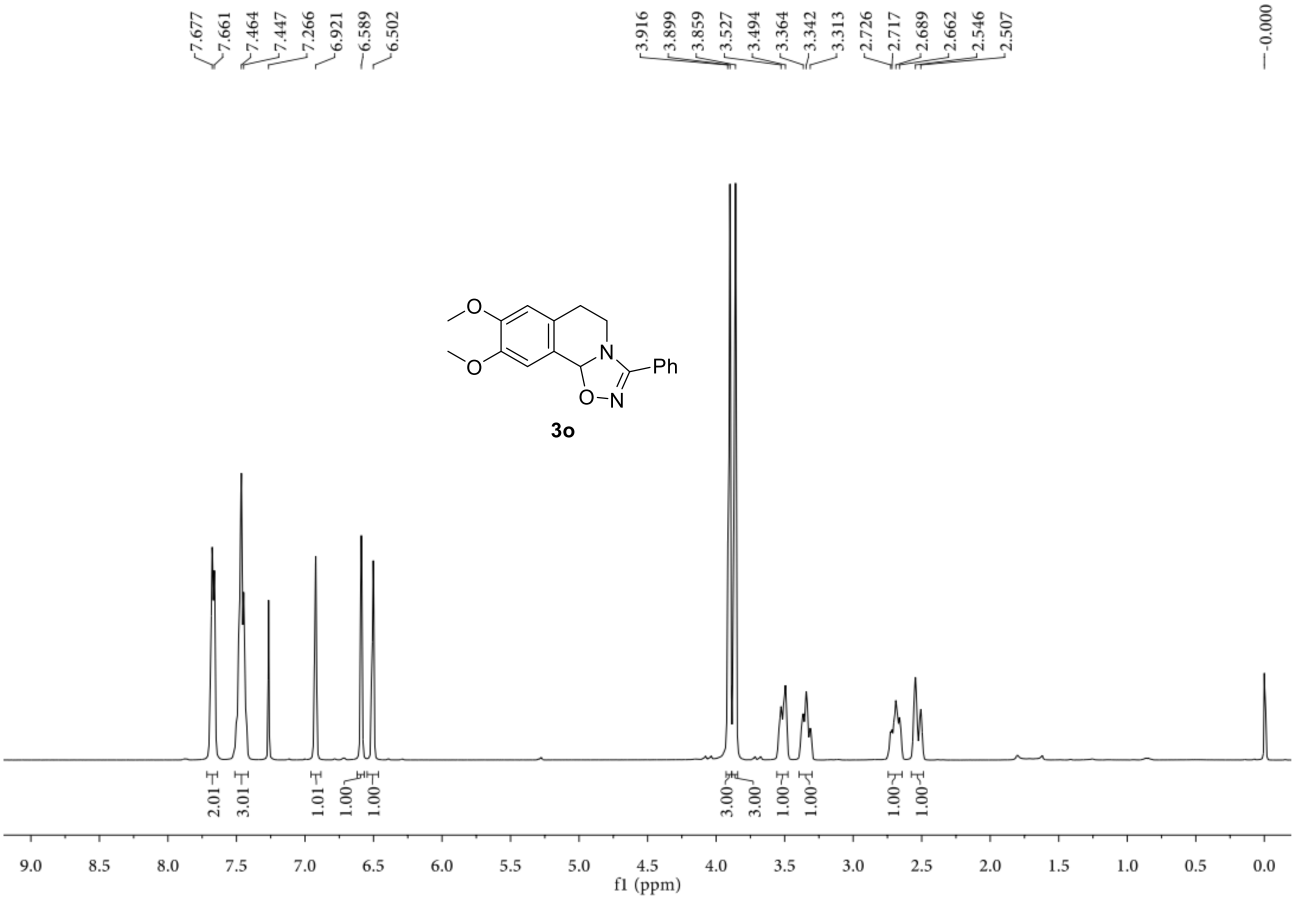
7.677
7.661
7.464
7.447
7.266
6.921
6.589
6.502
3.916
3.899
3.859
3.527
3.494
3.364
3.342
3.313
2.726
2.717
2.689
2.662
2.546
2.507
-0.000
O
O
N
Ph
O
N
3o
2.01
3.01
1.01
1.00
1.00
3.00
3.00
1.00
1.00
1.00
1.00
9.0
8.5
8.0
7.5
7.0
6.5
6.0
5.5
5.0
4.5
4.0
3.5
3.0
2.5
2.0
1.5
1.0
0.5
0.0
f1 (ppm)

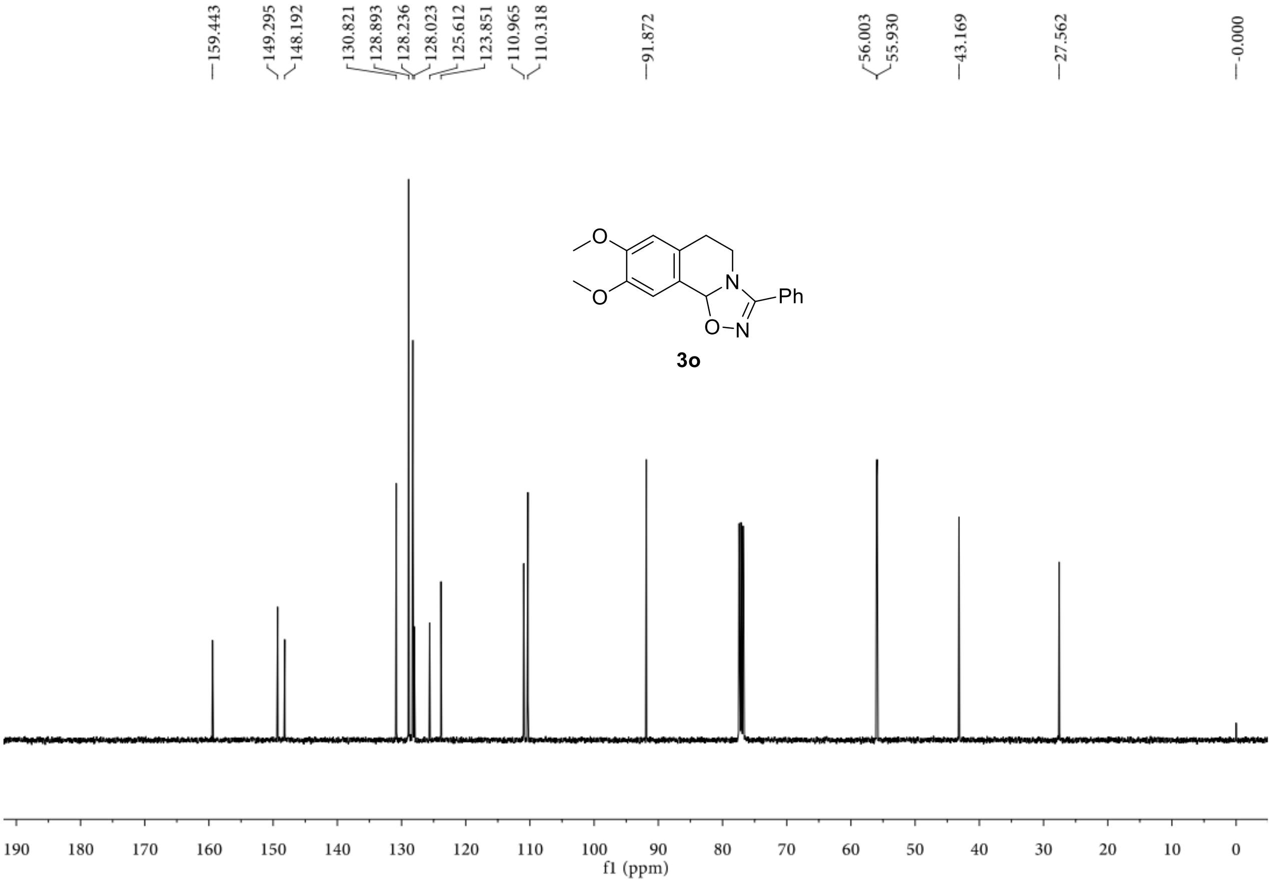
159.443
149.295
148.192
130.821
128.893
128.236
128.023
125.612
123.851
110.965
110.318
91.872
56.003
55.930
43.169
27.562
-0.000
O
O
N
Ph
O
N
3o
190
180
170
160
150
140
130
120
110
100
90
80
70
60
50
40
30
20
10
0
f1 (ppm)

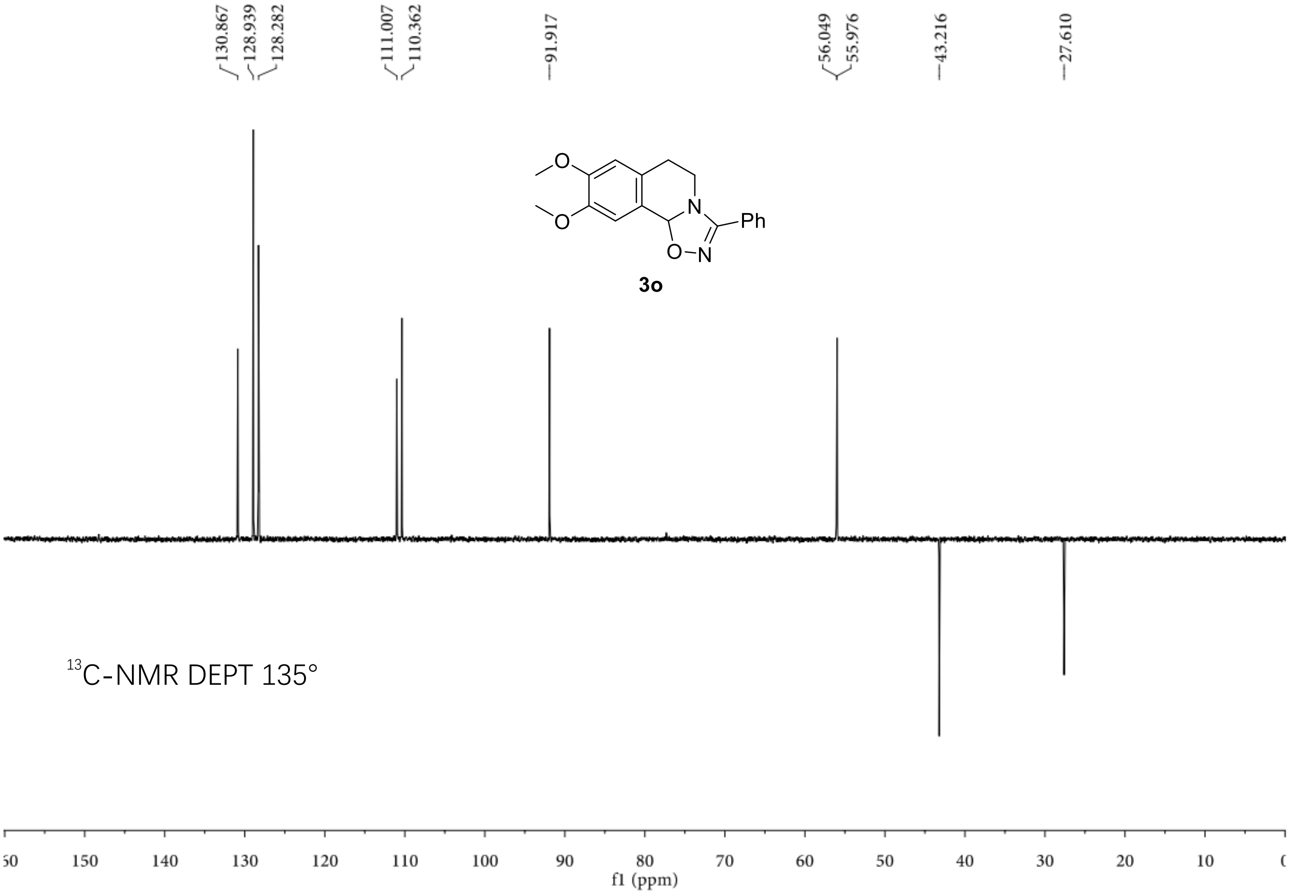
130.867
128.939
128.282
111.007
110.362
91.917
56.049
55.976
43.216
27.610
O
O
N
Ph
O
N
3o
13C-NMR DEPT 135°
150
140
130
120
110
100
90
80
70
60
50
40
30
20
10
f1 (ppm)

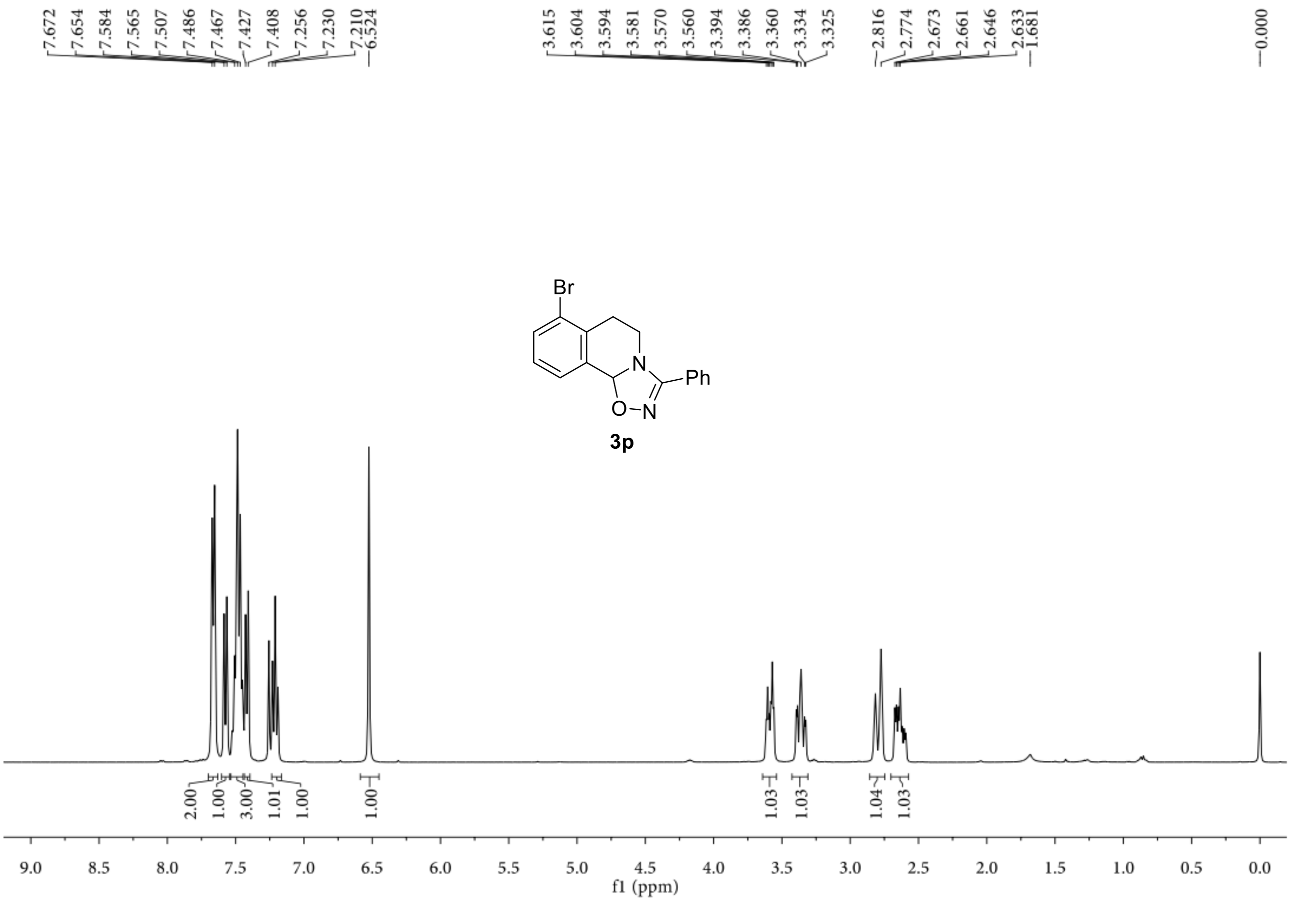
7.672
7.654
7.584
7.565
7.507
7.486
7.467
7.427
7.408
7.256
7.230
7.210
6.524
3.615
3.604
3.594
3.581
3.570
3.560
3.394
3.386
3.360
3.334
3.325
2.816
2.774
2.673
2.661
2.646
2.633
1.681
0.000
Br
N
Ph
O
N
3p
2.00
1.00
3.00
1.01
1.00
1.00
1.03
1.03
1.04
1.03
9.0 8.5 8.0 7.5 7.0 6.5 6.0 5.5 5.0 4.5 4.0 3.5 3.0 2.5 2.0 1.5 1.0 0.5 0.0
f1 (ppm)

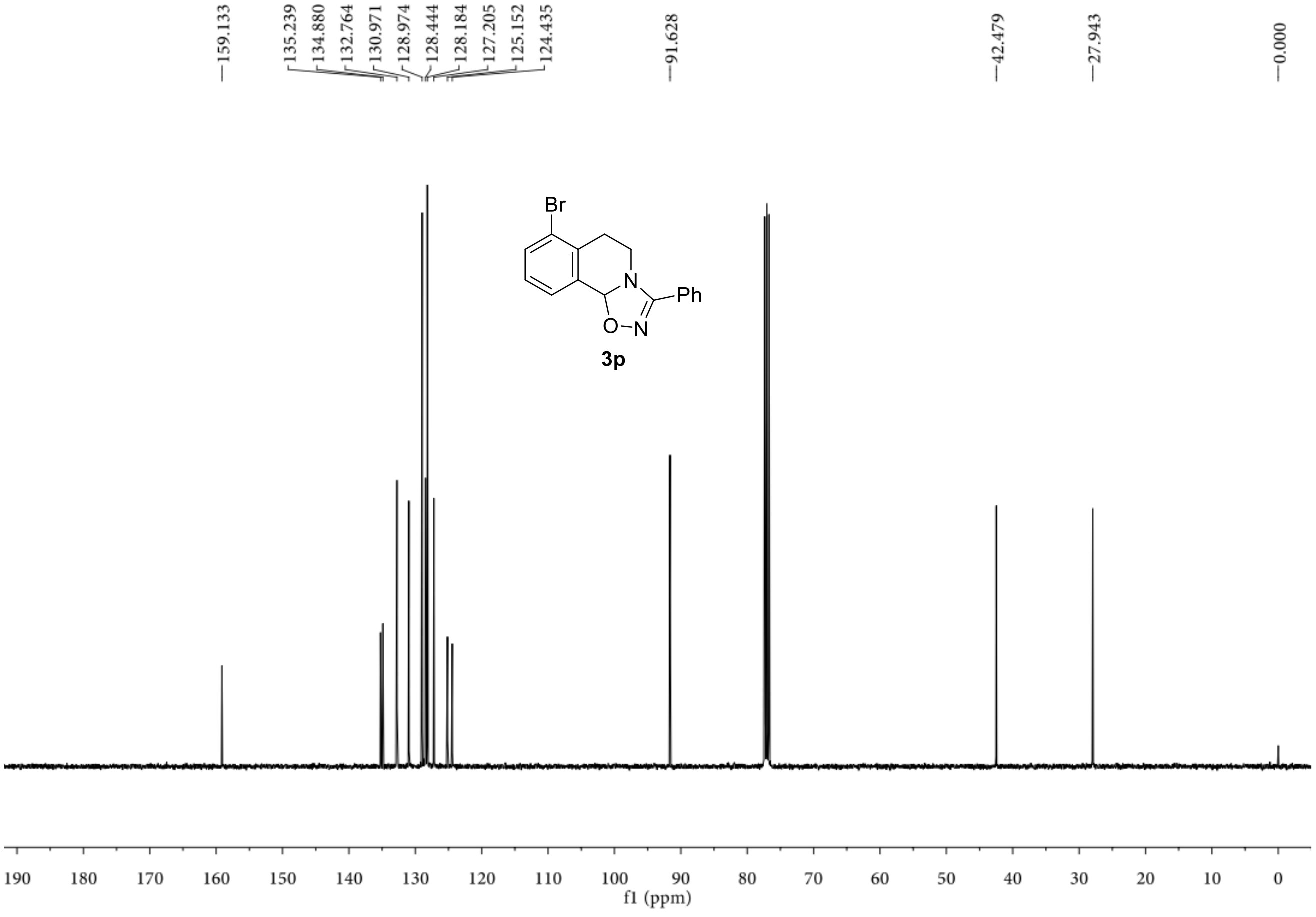
159.133
135.239
134.880
132.764
130.971
128.974
128.444
128.184
127.205
125.152
124.435
91.628
42.479
27.943
0.000
Br
N
Ph
O
N
3p
190 180 170 160 150 140 130 120 110 100 90 80 70 60 50 40 30 20 10 0
f1 (ppm)

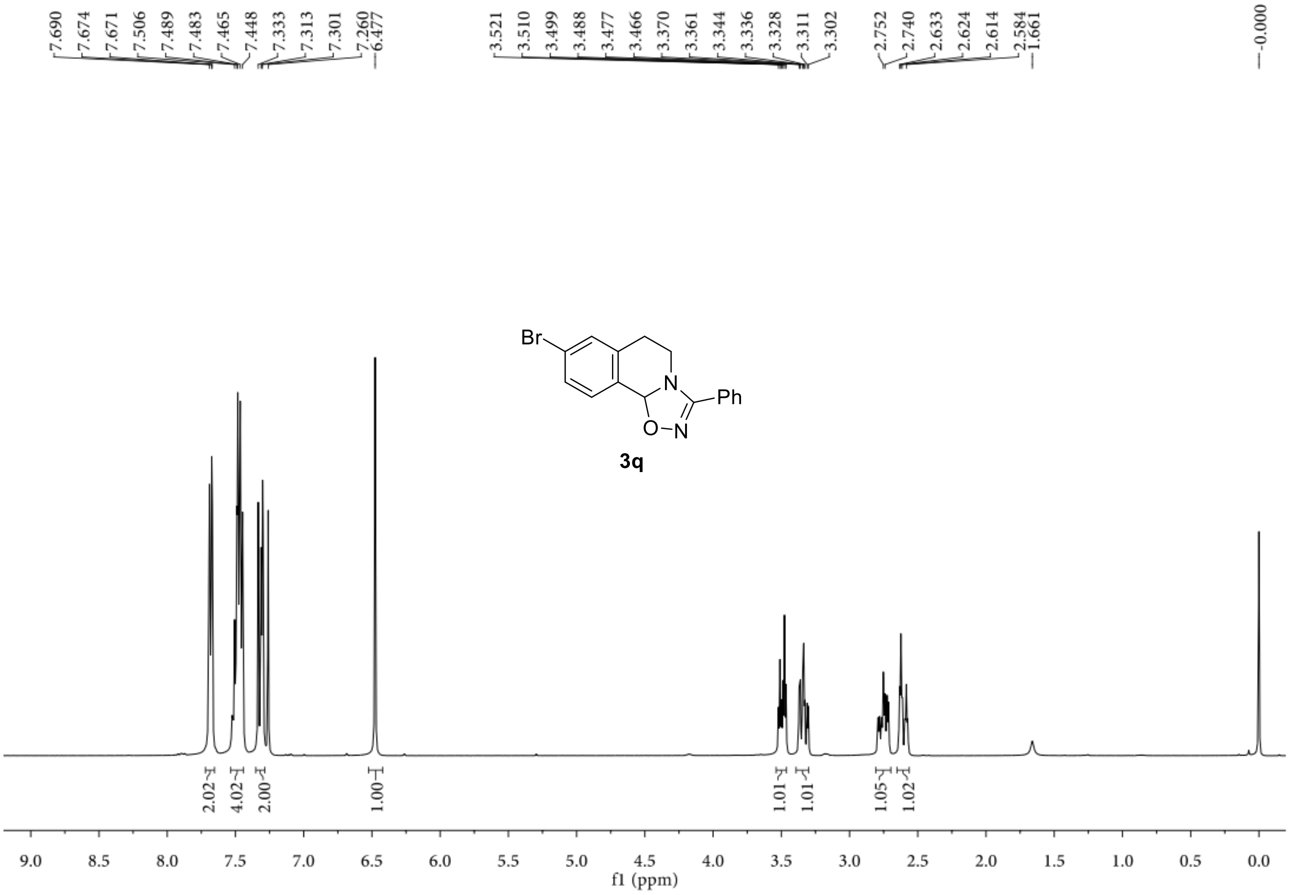
7.690
7.674
7.671
7.506
7.489
7.483
7.465
7.448
7.333
7.313
7.301
7.260
6.477
3.521
3.510
3.499
3.488
3.477
3.466
3.370
3.361
3.344
3.336
3.328
3.311
3.302
2.752
2.740
2.633
2.624
2.614
2.584
1.661
-0.000
Br
N
Ph
O
N
3q
2.02
4.02
2.00
1.00
1.01
1.01
1.05
1.02
9.0 8.5 8.0 7.5 7.0 6.5 6.0 5.5 5.0 4.5 4.0 3.5 3.0 2.5 2.0 1.5 1.0 0.5 0.0
f1 (ppm)

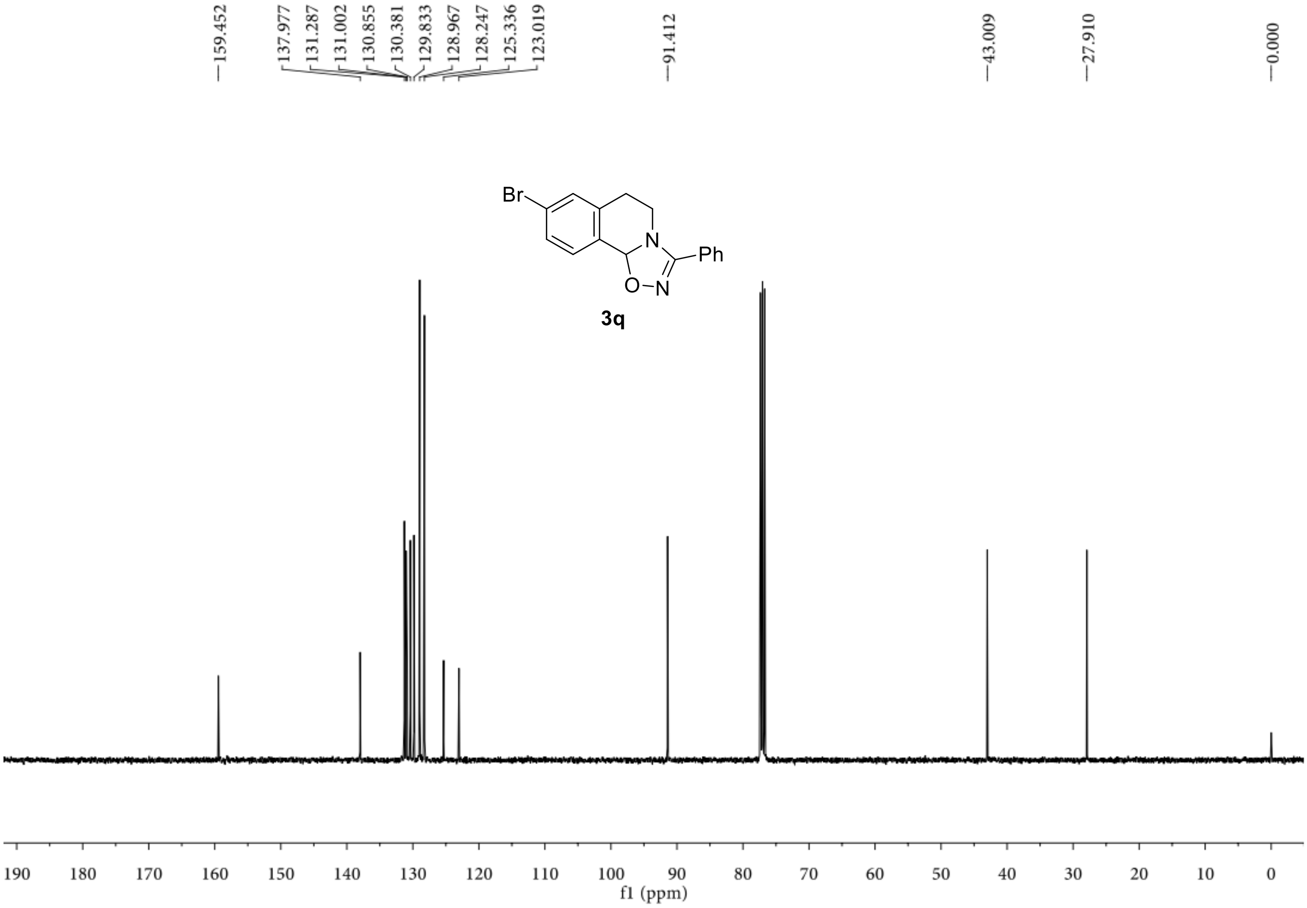
159.452
137.977
131.287
131.002
130.855
130.381
129.833
128.967
128.247
125.336
123.019
91.412
43.009
27.910
0.000
Br
N
Ph
O
N
3q
190 180 170 160 150 140 130 120 110 100 90 80 70 60 50 40 30 20 10 0
f1 (ppm)

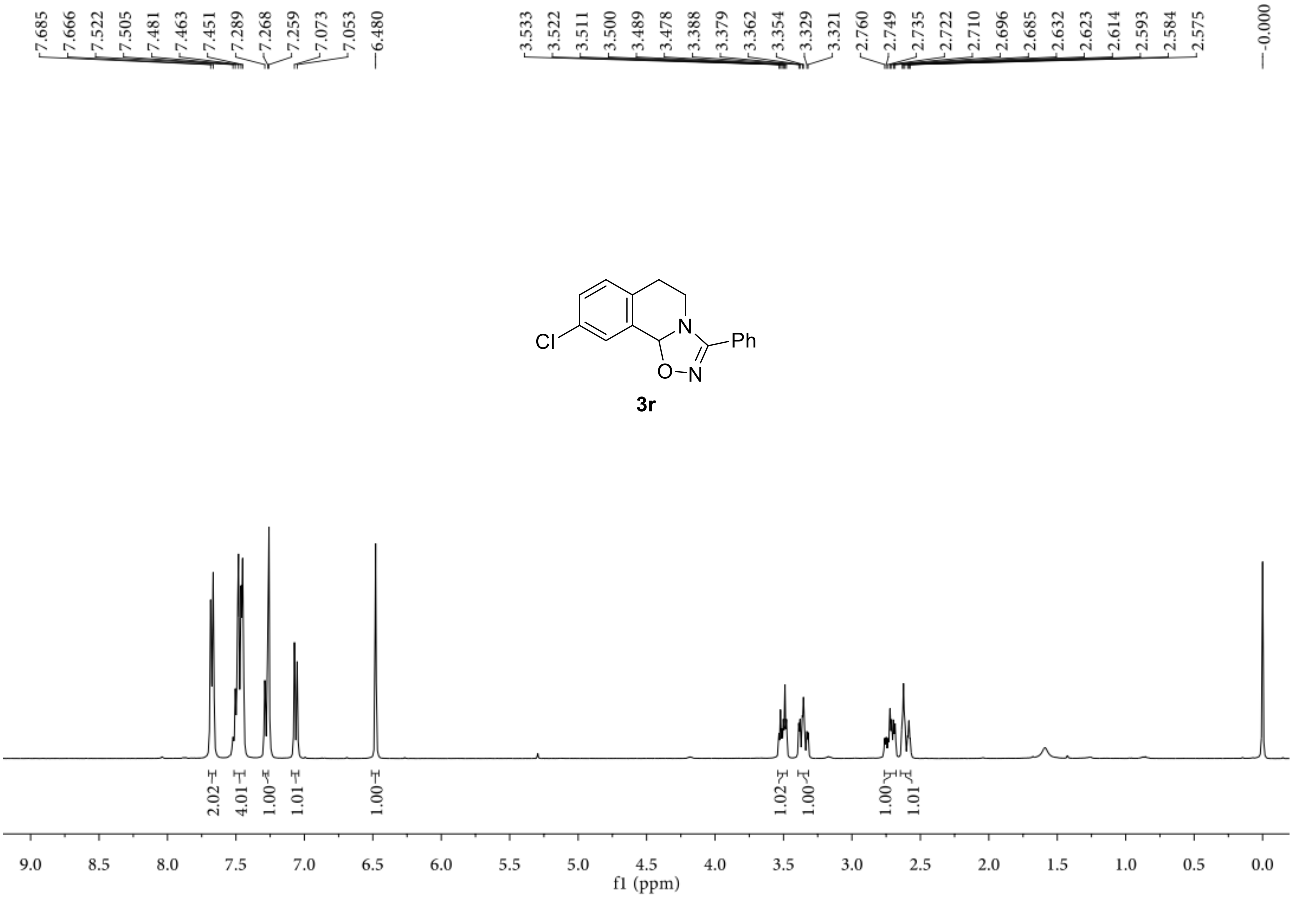
7.685
7.666
7.522
7.505
7.481
7.463
7.451
7.289
7.268
7.259
7.073
7.053
6.480
3.533
3.522
3.511
3.500
3.489
3.478
3.388
3.379
3.362
3.354
3.329
3.321
2.760
2.749
2.735
2.722
2.710
2.696
2.685
2.632
2.623
2.614
2.593
2.584
2.575
-0.000
Cl
N
Ph
O
N
3r
2.02
4.01
1.00
1.01
1.00
1.02
1.00
1.00
1.01
9.0
8.5
8.0
7.5
7.0
6.5
6.0
5.5
5.0
4.5
4.0
3.5
3.0
2.5
2.0
1.5
1.0
0.5
0.0
f1 (ppm)

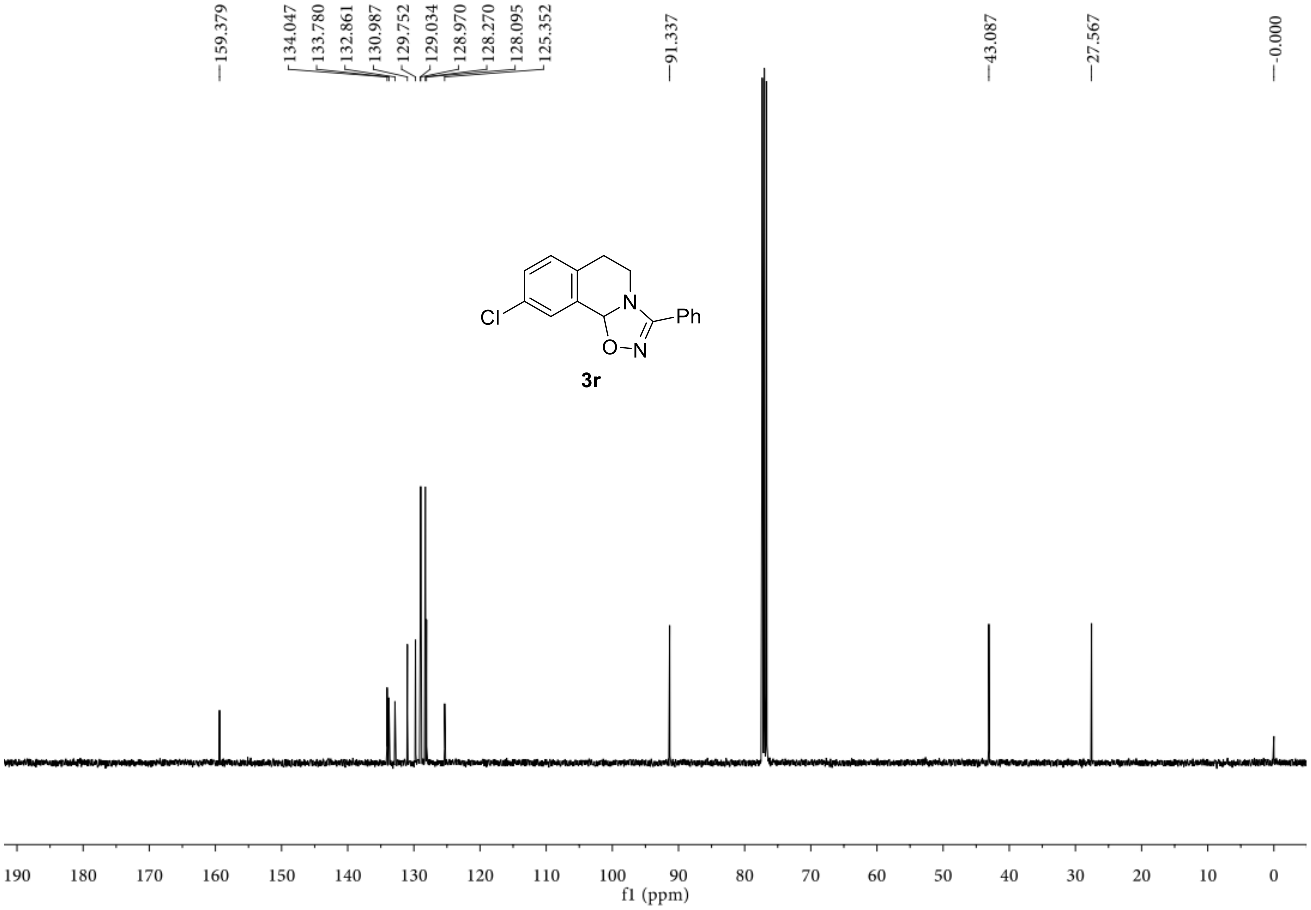
159.379
134.047
133.780
132.861
130.987
129.752
129.034
128.970
128.270
128.095
125.352
91.337
43.087
27.567
-0.000
Cl
N
Ph
O
N
3r
190
180
170
160
150
140
130
120
110
100
90
80
70
60
50
40
30
20
10
0
f1 (ppm)

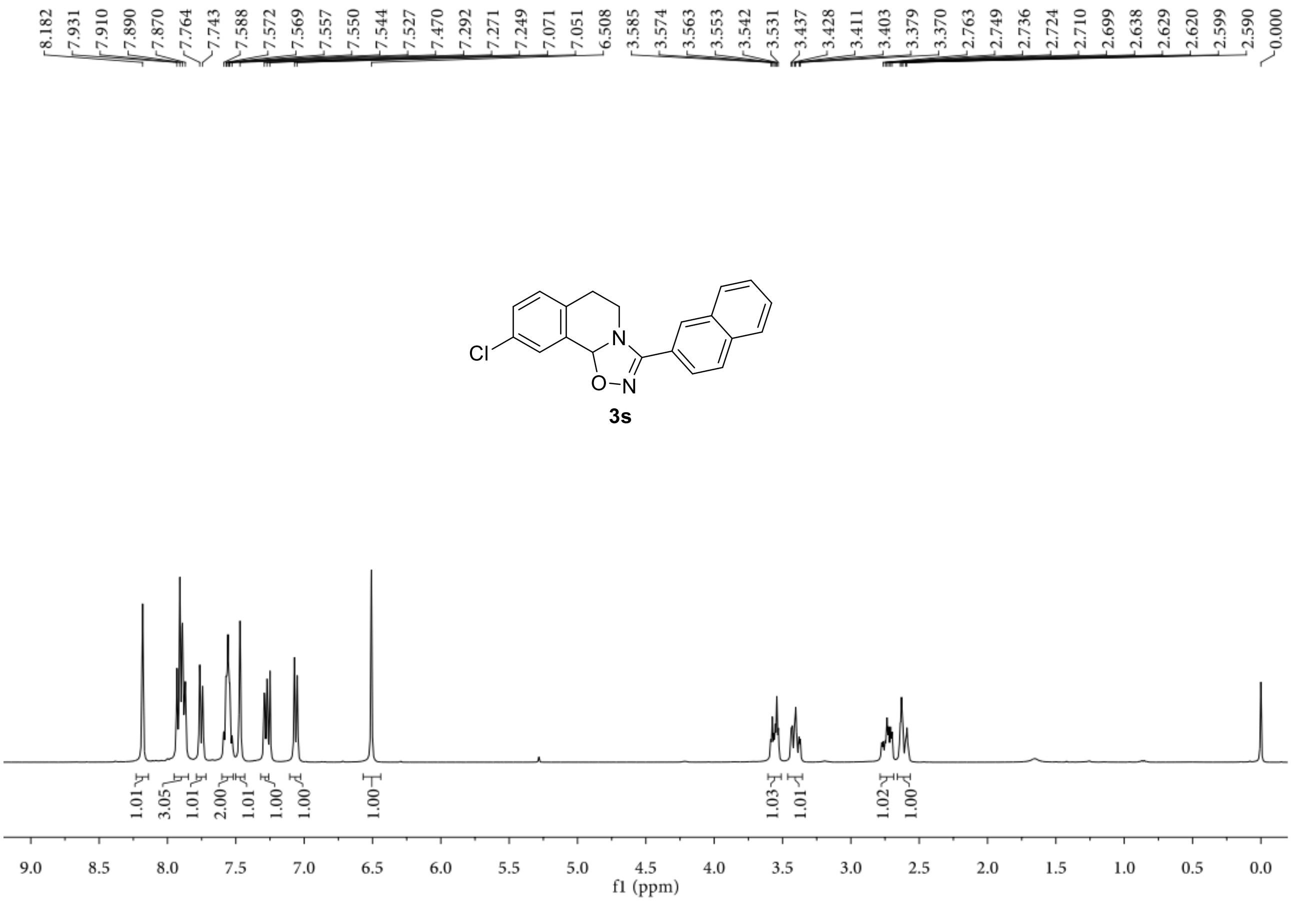
8.182 7.931 7.910 7.890 7.870 7.764 7.743 7.588 7.572 7.569 7.557 7.550 7.544 7.527 7.470 7.292 7.271 7.249 7.071 7.051 6.508 3.585 3.574 3.563 3.553 3.542 3.531 3.437 3.428 3.411 3.403 3.379 3.370 2.763 2.749 2.736 2.724 2.710 2.699 2.638 2.629 2.620 2.599 2.590 0.000
Cl
N
O
N
3s
1.01 3.05 1.01 2.00 1.01 1.00 1.00 1.00 1.03 1.01 1.02 1.00
9.0 8.5 8.0 7.5 7.0 6.5 6.0 5.5 5.0 4.5 4.0 3.5 3.0 2.5 2.0 1.5 1.0 0.5 0.0
f1 (ppm)

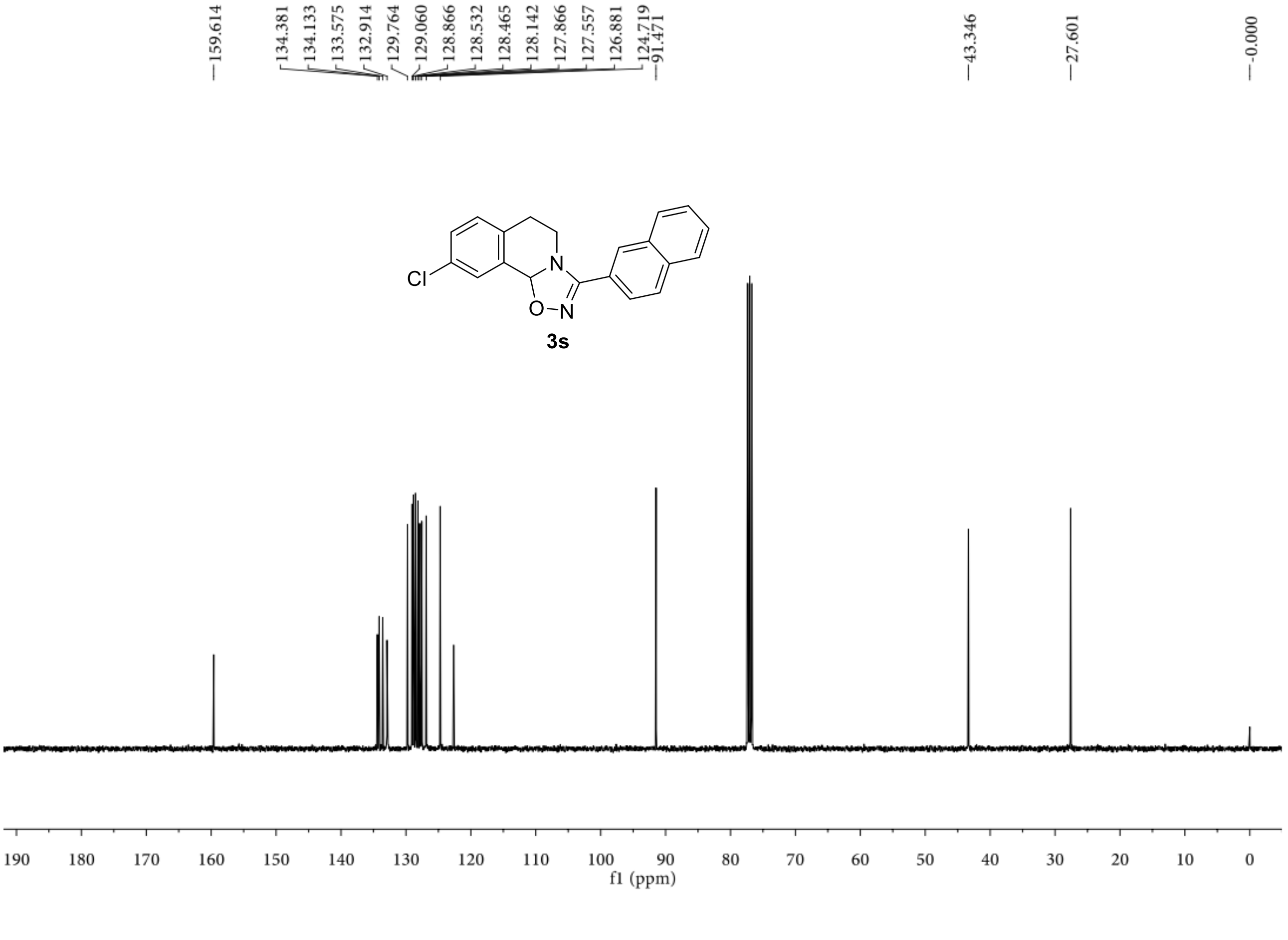
159.614 134.381 134.133 133.575 132.914 129.764 129.060 128.866 128.532 128.465 128.142 127.866 127.557 126.881 124.719 91.471 43.346 27.601 -0.000
Cl
N
O
N
3s
190 180 170 160 150 140 130 120 110 100 90 80 70 60 50 40 30 20 10 0
f1 (ppm)

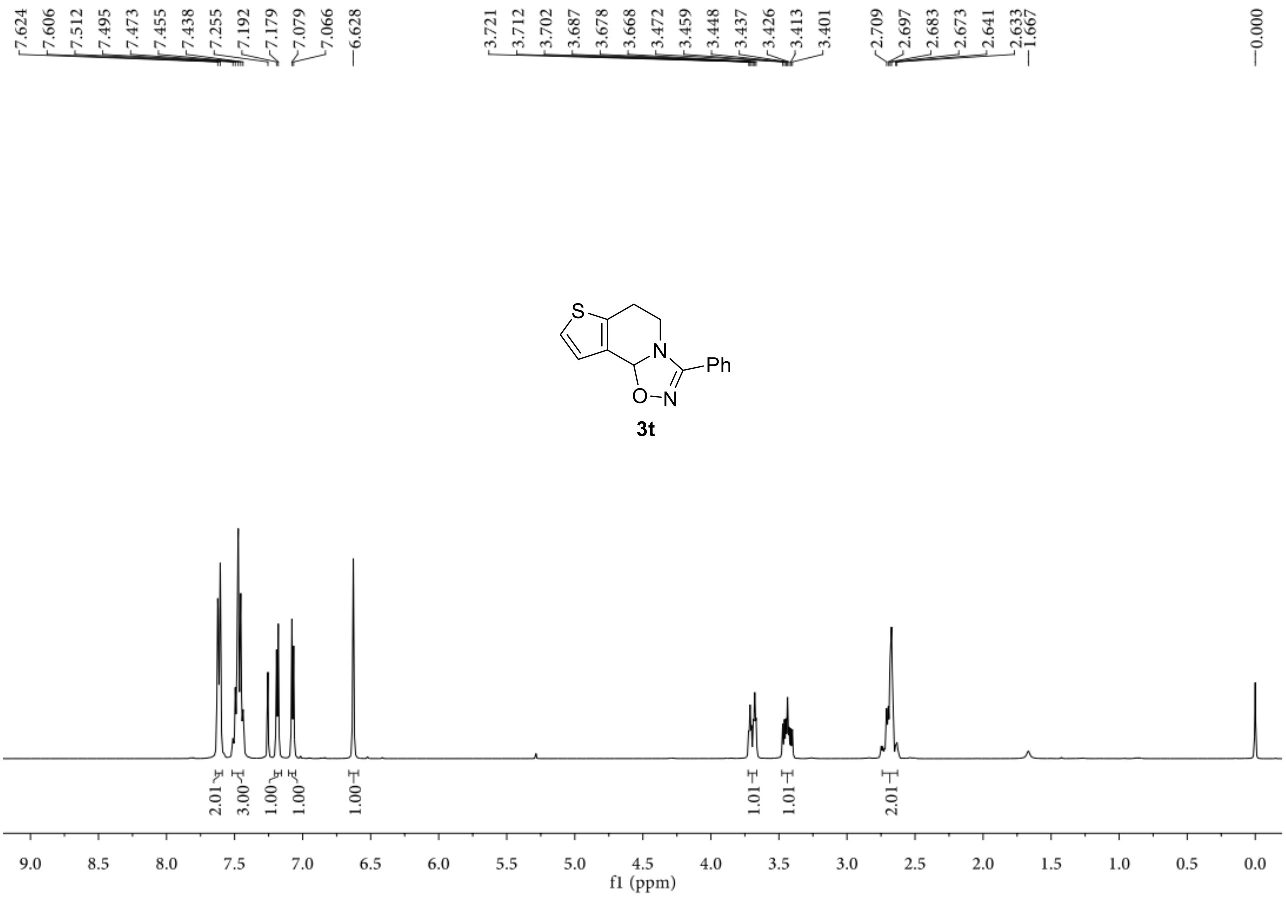

3t

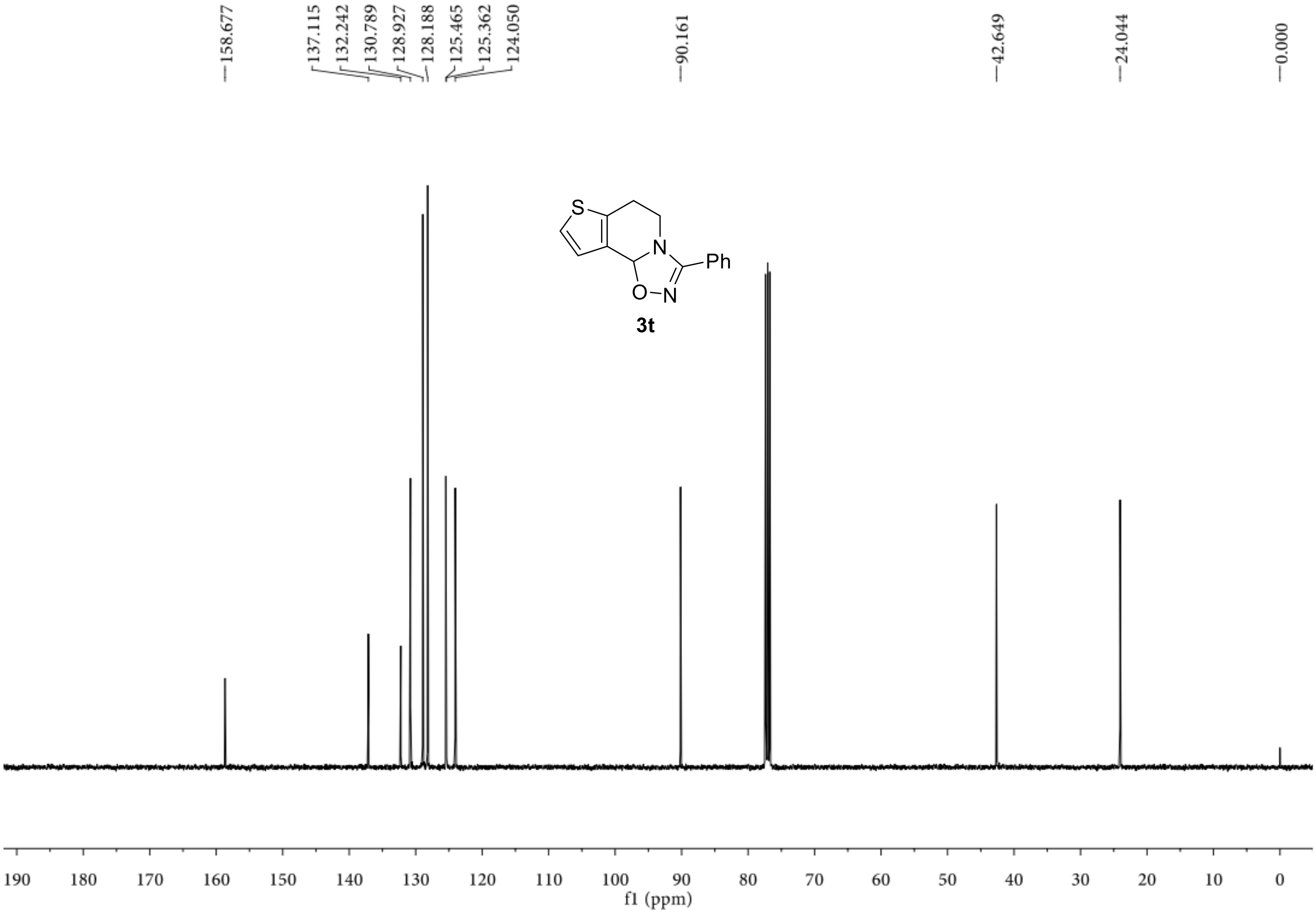

3t

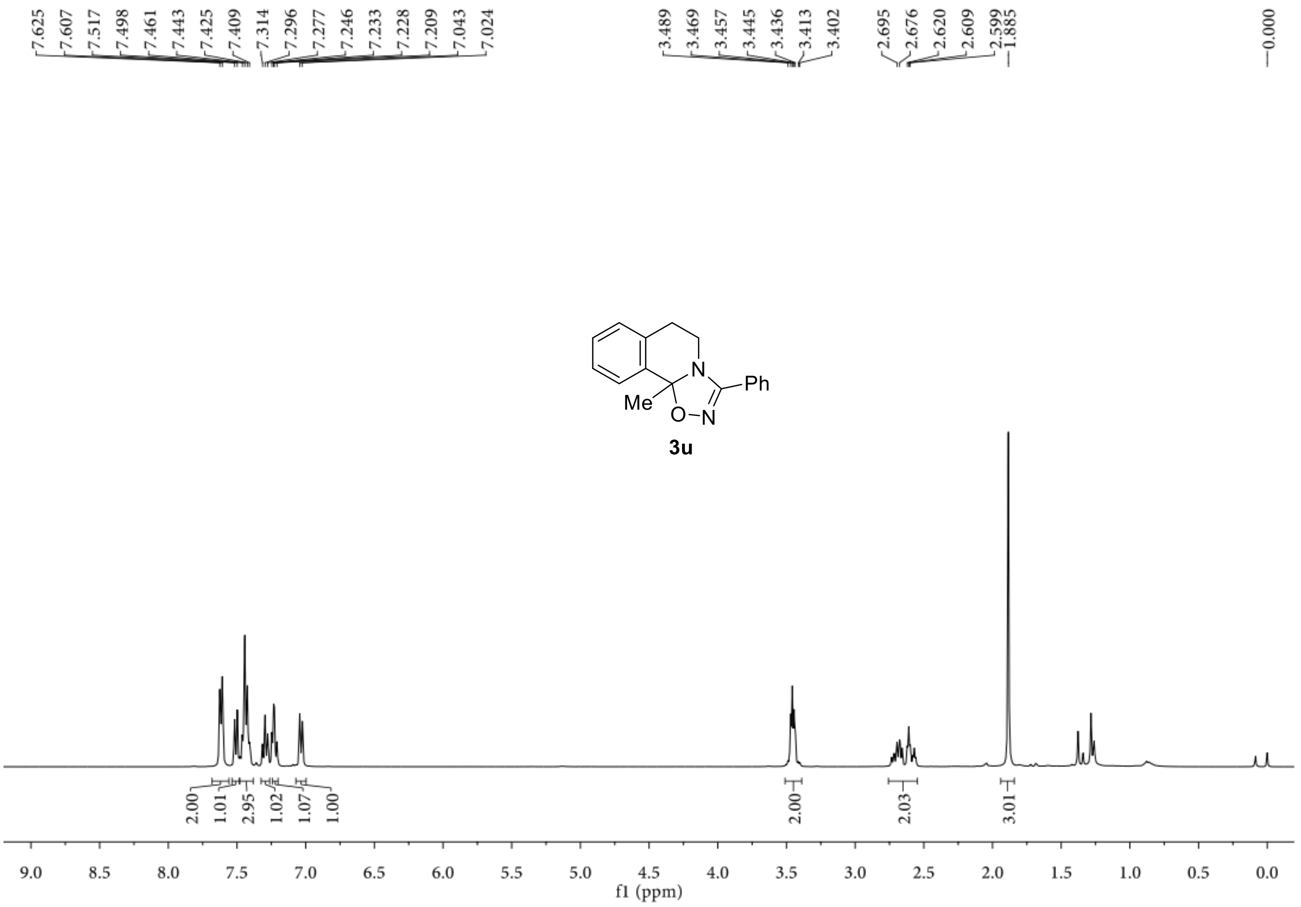
7.625 7.607 7.517 7.498 7.461 7.443 7.425 7.409 7.314 7.296 7.277 7.246 7.233 7.228 7.209 7.043 7.024
3.489 3.469 3.457 3.445 3.436 3.413 3.402
2.695 2.676 2.620 2.609 2.599 1.885
0.000
Me
O
N
N
Ph
3u
2.00 1.01 2.95 1.02 1.07 1.00
2.00
2.03
3.01
f1 (ppm)

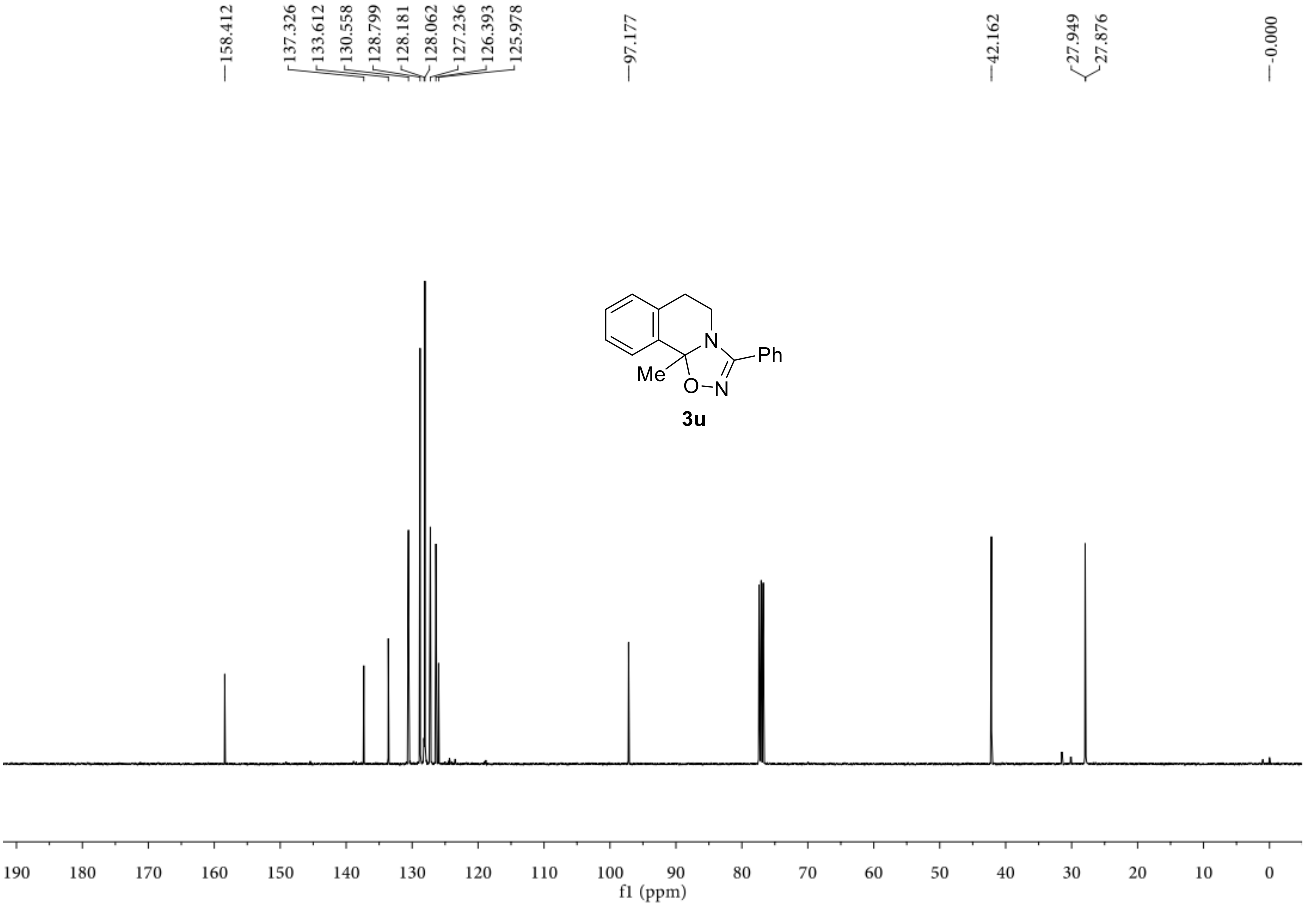
158.412
137.326 133.612 130.558 128.799 128.181 128.062 127.236 126.393 125.978
97.177
42.162
27.949 27.876
-0.000
Me
O
N
N
Ph
3u
f1 (ppm)

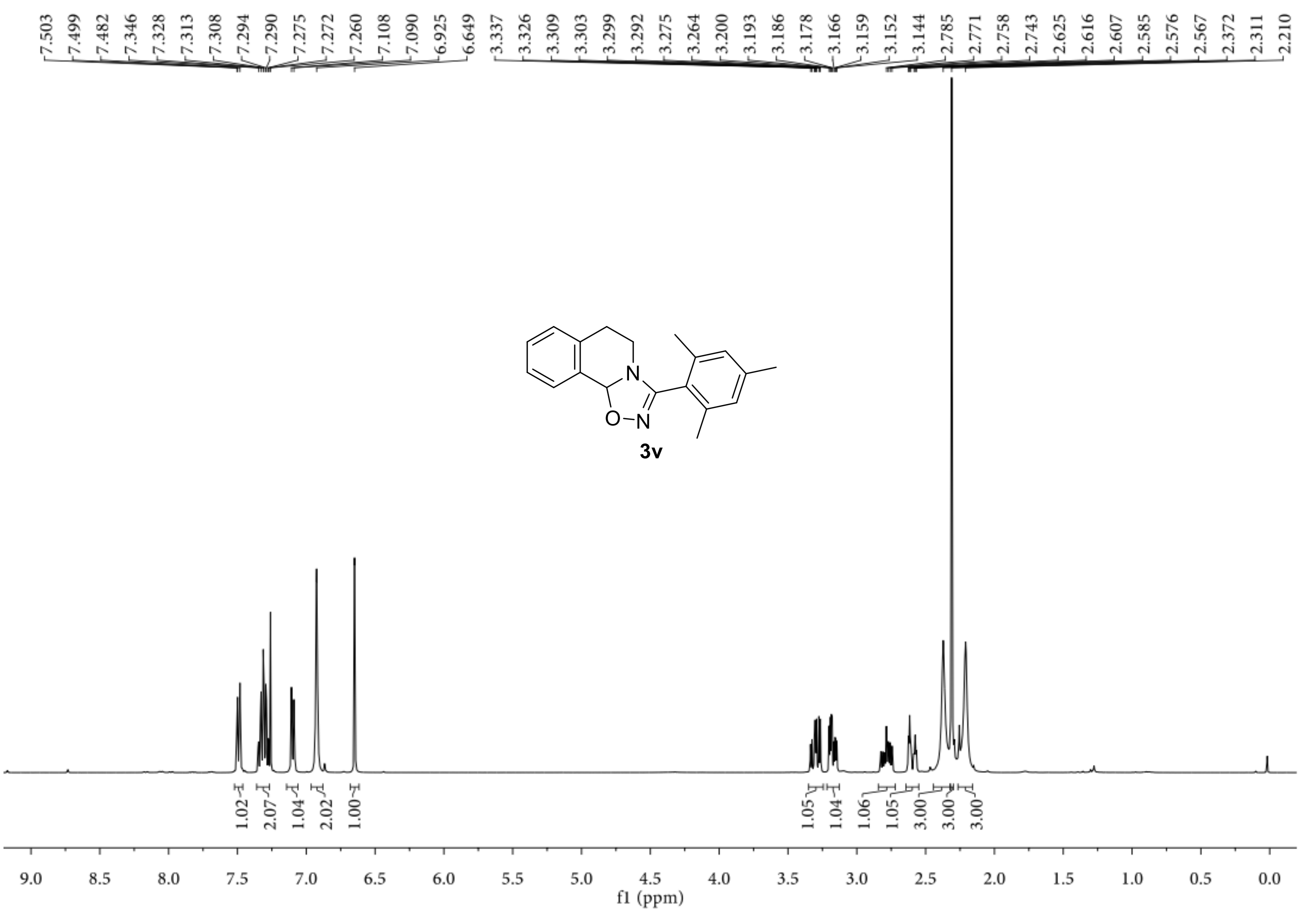
7.503
7.499
7.482
7.346
7.328
7.313
7.308
7.294
7.290
7.275
7.272
7.260
7.108
7.090
6.925
6.649
3.337
3.326
3.309
3.303
3.299
3.292
3.275
3.264
3.200
3.193
3.186
3.178
3.166
3.159
3.152
3.144
2.785
2.771
2.758
2.743
2.625
2.616
2.607
2.585
2.576
2.567
2.372
2.311
2.210
3v
1.02
2.07
1.04
2.02
1.00
1.05
1.04
1.06
1.05
3.00
3.00
3.00
f1 (ppm)

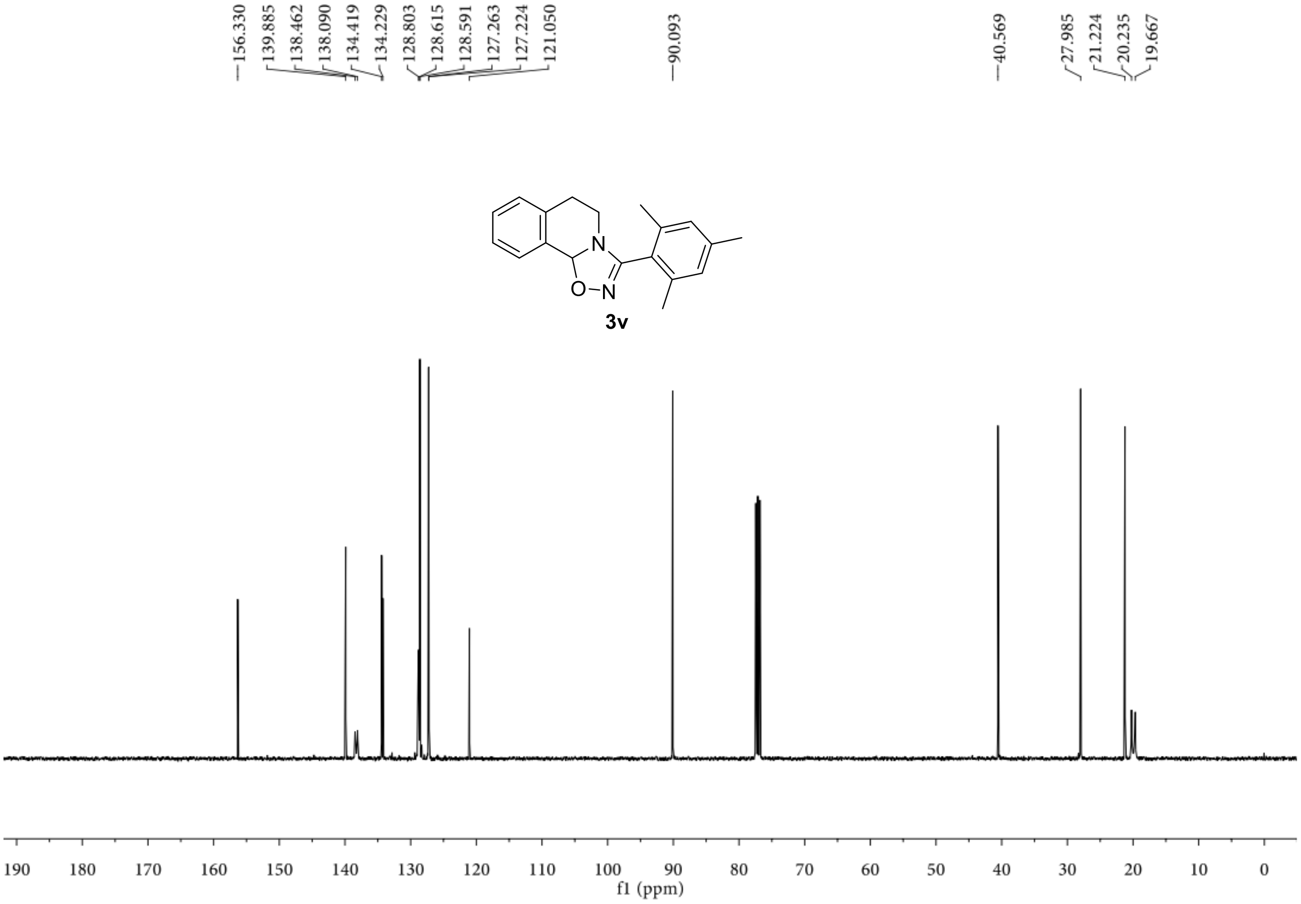
156.330
139.885
138.462
138.090
134.419
134.229
128.803
128.615
128.591
127.263
127.224
121.050
90.093
40.569
27.985
21.224
20.235
19.667
3v
f1 (ppm)

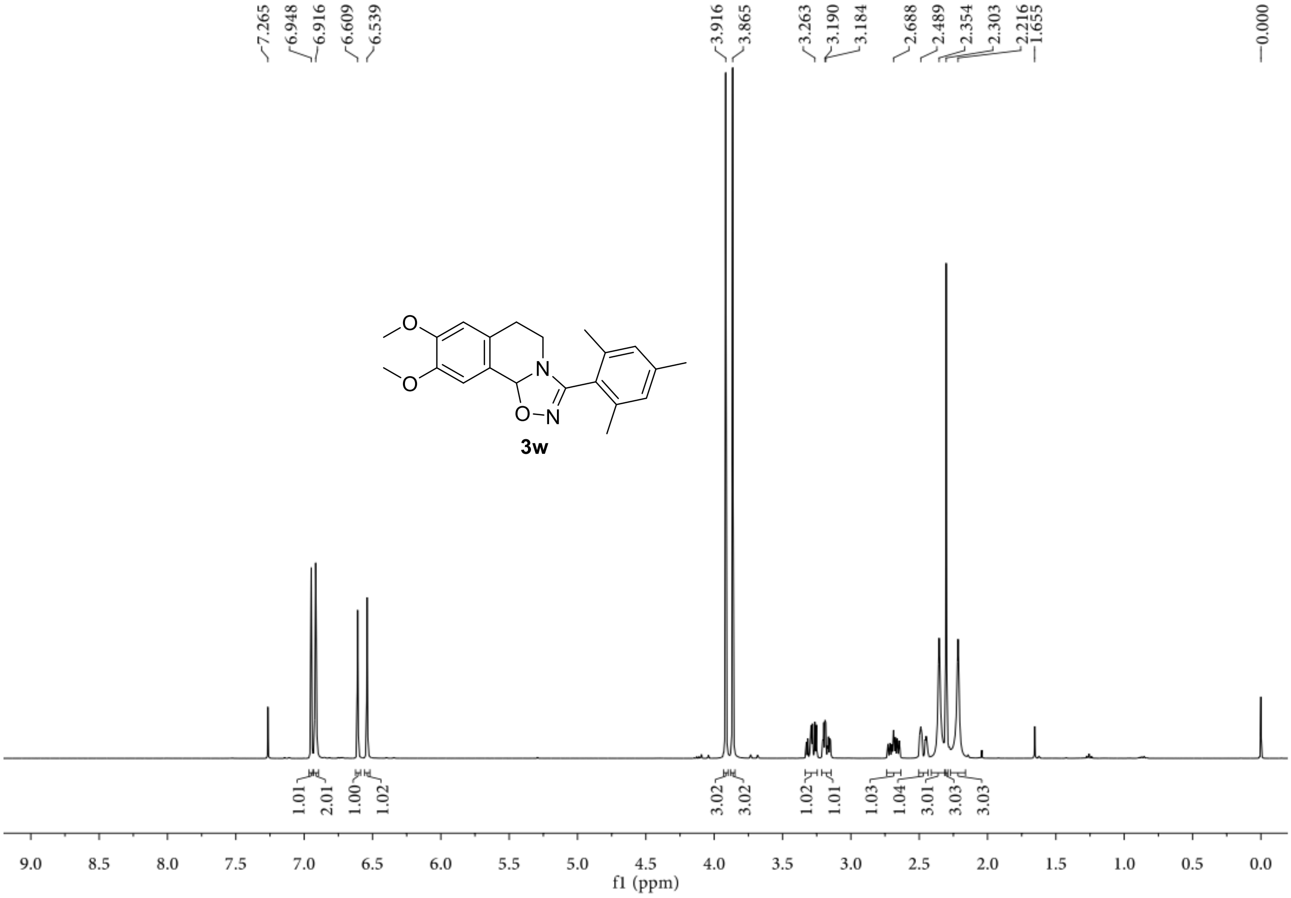
7.265
6.948
6.916
6.609
6.539
3.916
3.865
3.263
3.190
3.184
2.688
2.489
2.354
2.303
2.216
1.655
0.000
O
N
O–N
3w
1.01
2.01
1.00
1.02
3.02
3.02
1.02
1.01
1.03
1.04
3.01
3.03
3.03
9.0 8.5 8.0 7.5 7.0 6.5 6.0 5.5 5.0 4.5 4.0 3.5 3.0 2.5 2.0 1.5 1.0 0.5 0.0
f1 (ppm)

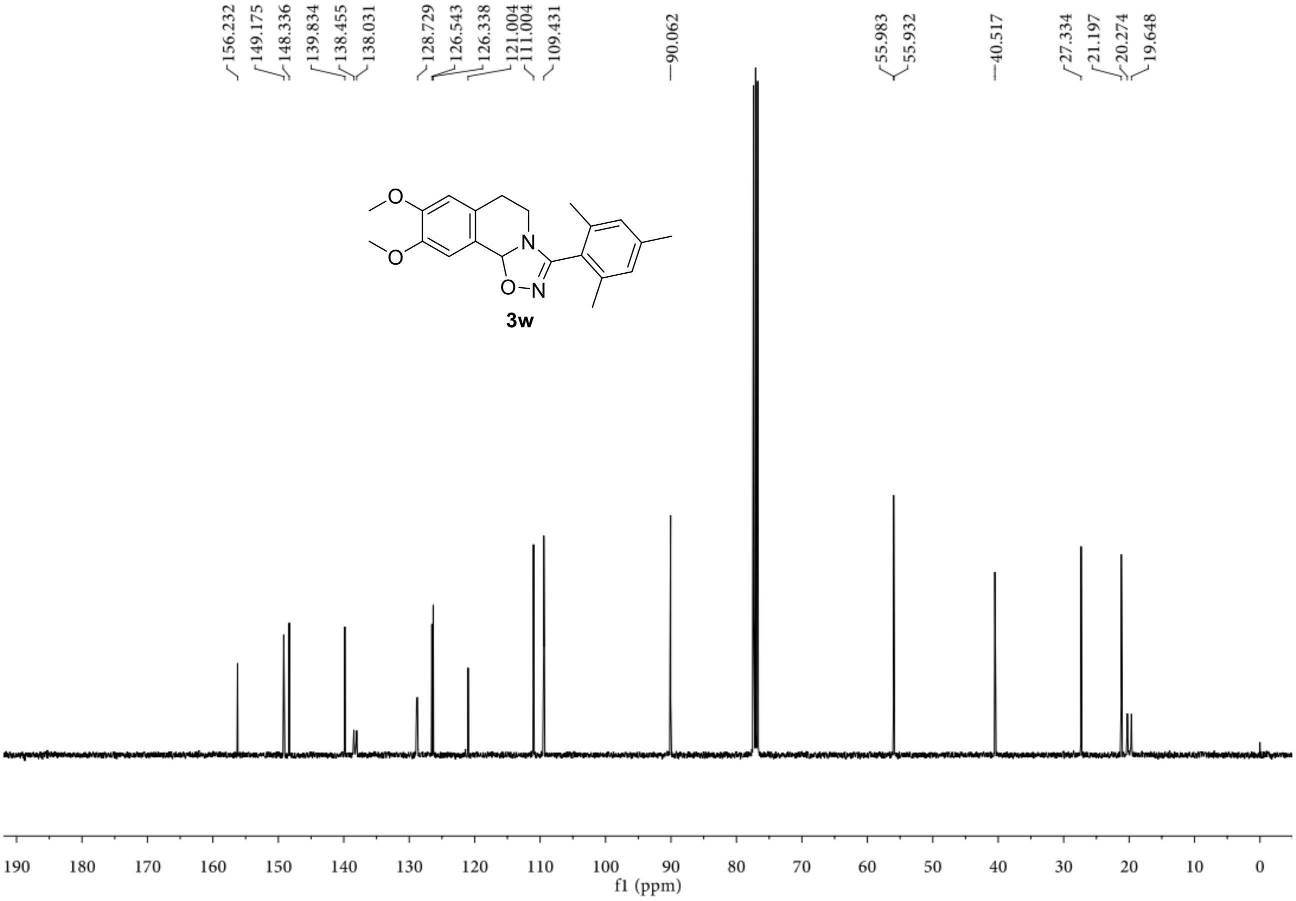
156.232
149.175
148.336
139.834
138.455
138.031
128.729
126.543
126.338
121.004
111.004
109.431
90.062
55.983
55.932
40.517
27.334
21.197
20.274
19.648
O
N
O–N
3w
190 180 170 160 150 140 130 120 110 100 90 80 70 60 50 40 30 20 10 0
f1 (ppm)

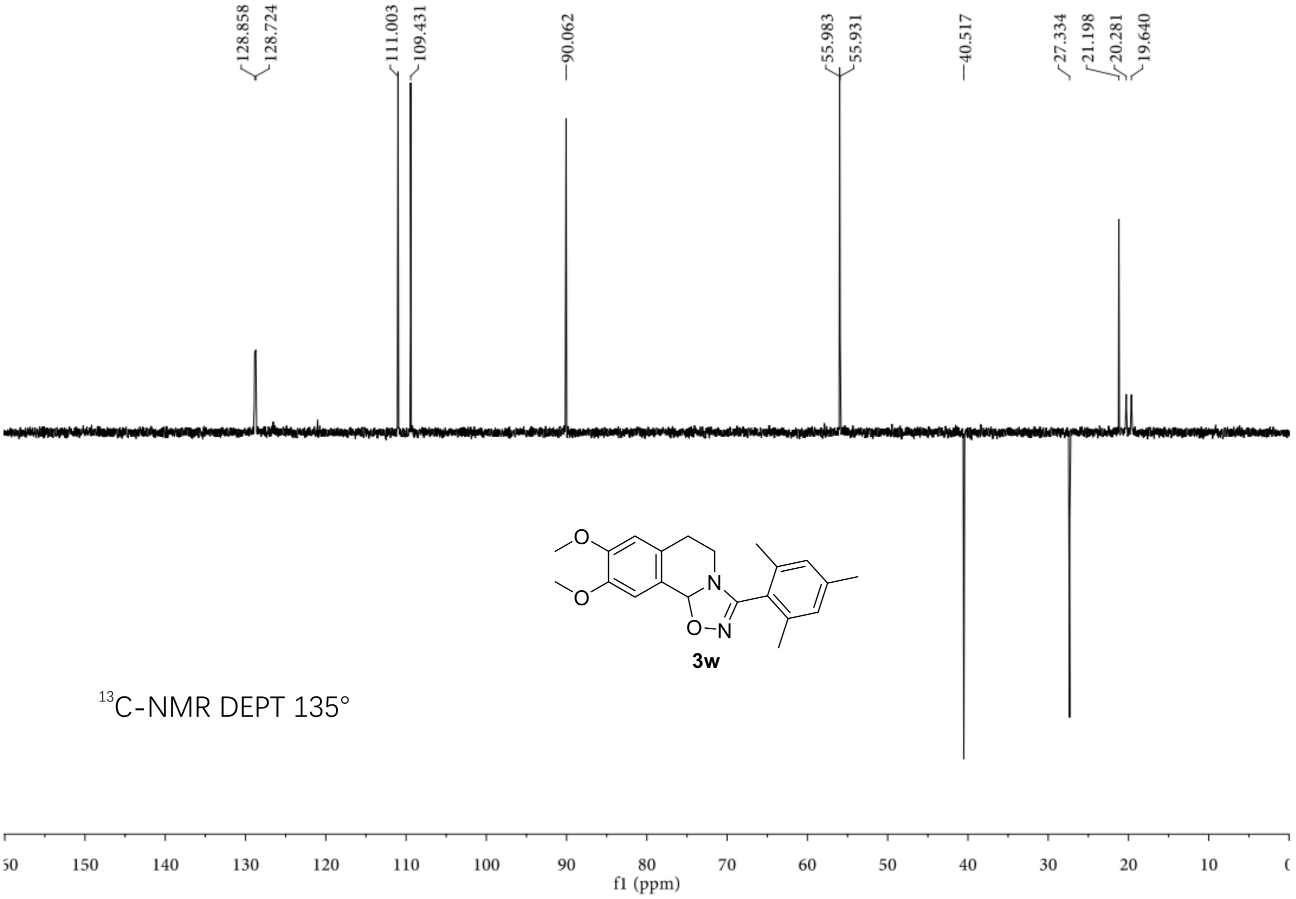
128.858
128.724
111.003
109.431
90.062
55.983
55.931
40.517
27.334
21.198
20.281
19.640
3w
^{13}C-NMR DEPT 135°
150
140
130
120
110
100
90
80
70
60
50
40
30
20
10
f1 (ppm)
